# HIV Protein Tat Induces Macrophage Dysfunction and Atherosclerosis Development in Low-Density Lipoprotein Receptor-Deficient Mice

**DOI:** 10.1007/s10557-021-07141-x

**Published:** 2021-01-18

**Authors:** Zhaojie Meng, Rebecca Hernandez, Jingwei Liu, Taesik Gwag, Weiwei Lu, Tzung K Hsiai, Marcus Kaul, Tong Zhou, Changcheng Zhou

**Affiliations:** 1 Division of Biomedical Sciences, School of Medicine, University of California, Riverside, CA; 2 Department of Pharmacology and Nutritional Sciences, College of Medicine, University of Kentucky, Lexington, KY; 3 Departments of Medicine and Bioengineering, David Geffen School of Medicine, Henry Samueli School of Engineering and Applied Science, University of California, Los Angeles, CA; 4 Department of Physiology and Cell Biology, Reno School of Medicine, University of Nevada, Reno, NV

**Keywords:** HIV infection, atherosclerosis, inflammation, IκB kinase β, macrophages, nuclear factor-κB

## Abstract

**Purpose:**

HIV infection is consistently associated with an increased risk of atherosclerotic cardiovascular disease, but the underlying mechanisms remain elusive. HIV protein Tat, a transcriptional activator of HIV, has been shown to activate NF-κB signaling and promote inflammation in vitro. However, the atherogenic effects of HIV Tat have not been investigated in vivo. Macrophages are one of the major cell types involved in the initiation and progression of atherosclerosis. We and others have previously revealed the important role of IκB kinase β (IKKβ), a central inflammatory coordinator through activating NF-κB, in the regulation of macrophage functions and atherogenesis. This study investigated the impact of HIV Tat exposure on macrophage functions and atherogenesis.

**Methods:**

To investigate the effects of Tat on macrophage IKKβ activation and atherosclerosis development in vivo, myeloid-specific IKKβ-deficient LDLR-deficient (IKKβ^ΔMye^LDLR^−/−^) mice and their control littermates (IKKβ^F/F^LDLR^−/−^) were exposed to recombinant HIV protein Tat.

**Results:**

Exposure to Tat significantly increased atherosclerotic lesion size and plaque vulnerability in IKKβ^F/F^LDLR^−/−^ but not IKKβ^ΔMye^LDLR^−/−^ mice. Deficiency of myeloid IKKβ attenuated Tat-elicited macrophage inflammatory responses and atherosclerotic lesional inflammation in IKKβ^ΔMye^LDLR^−/−^ mice. Further, RNAseq analysis demonstrated that HIV protein Tat affects the expression of many atherosclerosis-related genes in vitro in an IKKβ-dependent manner.

**Conclusions:**

Our findings reveal atherogenic effects of HIV protein Tat in vivo and demonstrate a pivotal role of myeloid IKKβ in Tat-driven atherogenesis.

## Introduction

The increase in long-term survival of HIV infection as a result of the introduction of effective antiretroviral therapy (ART) over the past decades has generated a new awareness of the impact of HIV infection on the development of other chronic diseases such as cardiovascular disease (CVD). Over the past two decades, the global burdens of HIV-associated CVD have tripled, and the morbidity and mortality from CVD pose serious challenges to HIV patients [1, 2]. It has been reported that HIV infection increases the risk of CVD by 61% as compared with healthy, HIV-naïve individuals [3]. A recent study of over 400,000 adults confirmed that HIV patients continue to be exposed to a higher risk of atherosclerotic CVD as compared to their counterparts without HIV [4]. A meta-analysis of many clinical studies revealed HIV patients with or without ART have a higher prevalence of atherosclerotic CVD as compared with the control population [3]. Several clinical studies also concluded that HIV infection is sufficient to aggravate atherosclerosis in the absence of ART or detectable viremia, or manifest immunodeficiency [5, 6].

Although extensive clinical studies have demonstrated exacerbated atherosclerosis development in HIV-infected patients, the cellular and molecular mechanisms of HIV-mediated atherogenesis remain poorly understood. The HIV encoded protein transactivator of transcription (Tat) is an early viral protein that stimulates transcription and is required for HIV replication [7, 8]. Tat proteins can be secreted into the extracellular microenvironment by HIV-infected T-cells and monocyte/macrophages [9, 10]. Tat can be detected in the serum of HIV patients, even with ART [9, 11]. Several studies have implicated the pro-atherogenic effects of Tat. For example, Tat has been shown to markedly decrease endothelium-dependent vasorelaxation and endothelial nitric oxide synthase production in porcine coronary arteries [8]. Aortic endothelial dysfunction and increased arterial stiffness, a surrogate marker of vascular risk [12], have been observed in transgenic mice expressing HIV viral proteins, including Tat [13]. Tat has also been shown to increase the adhesion of monocytes and T-cells to the endothelium in vitro and in vivo [14], and to promote cytokine production in monocytes/macrophages [15–17]. However, it has not been studied whether Tat can directly affect the development of atherosclerosis in appropriate animal models.

In addition to traditional risk factors, HIV-accelerated persistent immune activation and inflammation have been implicated as main factors promoting atherosclerosis [18, 19]. Inflammatory responses are the driving force of atherosclerosis development [20, 21], and many inflammatory pathways that contribute to atherogenesis are regulated by the transcription factor NF-κB, a master regulator of the innate and adaptive immune responses [22, 23, 21]. Canonical or classical NF-κB activation is regulated by IκB kinase (IKK) β, the predominant catalytic subunit of the IKK complex that mediates phosphorylation and degradation of the inhibitors of NF-κB (IκBs) [24, 21, 25]. We and others have previously revealed the important functions of IKKβ in regulating atherosclerosis and metabolic disorders [21, 26–31]. For example, deficiency of myeloid IKKβ has been shown to reduce macrophage inflammatory responses and to decrease diet-induced atherosclerosis in LDLR^−/−^ mice [26]. Deletion of IKKβ in smooth muscle cells protected LDLR^−/−^ mice from diet-induced vascular inflammation and atherosclerosis [21]. Overexpression of IKKβ in the liver can aggravate atherosclerosis development in APOE*3-Leiden mice [31]. More recently, we also found that IKKβ signaling in adipocytes can also affect the evolution of atherosclerosis plaque vulnerability in obese LDLR^−/−^ mice [29]. These studies demonstrated the important and complex functions of IKKβ signaling in atherogenesis.

Macrophages are the major inflammatory cells involved in the progression of atherosclerosis, and macrophage accumulation within the vascular wall is a hallmark of atherosclerosis [32, 33]. In addition to T cells, HIV also infects macrophages, which can remain chronically infected or activated even with ART [2, 34]. Transcriptome analysis also revealed elevated proinflammatory gene expression in monocytes and macrophages of HIV patients [34, 35]. HIV Tat has been demonstrated to activate NF-κB and induce the production of proinflammatory cytokines in monocytes and macrophages [16, 36]. However, it is not clear whether Tat can affect macrophage functions to regulate atherogenesis. In the present study, we used a myeloid-specific IKKβ-deficient LDLR^−/−^ mouse model to investigate the impact of HIV protein Tat on macrophage functions and atherosclerosis development. We demonstrate, for the first time to our knowledge, that exposure to Tat increased atherosclerosis in LDLR^−/−^ mice. Deficiency of myeloid IKKβ protected mice from Tat-induced atherosclerosis, most likely due to amelioration of Tat-elicited macrophage dysfunction.

## Materials and Methods

### Recombinant HIV protein Tat preparation

Recombinant HIV protein Tat was prepared as previously described [37, 38]. Tat is encoded by a gene consisting of two exons. The first exon contributes to the initial 72 amino acids and the second exon forms the remaining 14 to 32 amino acids. The functions of transactivation, neurotoxicity, and immune activation are all present within the first 72 amino acids formed by the first exon. Therefore, Tat_1–72_ was employed in the current study and prepared as described previously [37, 38]. Recombinant Tat_1–72_ was expressed as a fusion protein using an *Escherichia coli* vector after the induction of isopropyl β-D-1-thiogalactopyranoside (IPTG) (#2364, NIH AIDS Reagent Program, Germantown, MD). The control “placebo” solution was generated under the same condition as that of Tat proteins except for the induction of IPTG. Tat_1–72_ was purified by glutathione S-transferase-affinity chromatography (Glutathione Sepharose 4B, GE healthcare, Piscataway, NJ) and cleaved from the fusion protein by thrombin protease (GE healthcare, Piscataway, NJ). The Tat protein was >98% pure as determined by sodium dodecyl sulfate-polyacrylamide gel electrophoresis (SDS-PAGE) followed by Coomassie blue staining (Supplemental Figure 1A). The purified product was further confirmed by Western blot analysis using Anti-HIV Tat Monoclonal antibodies (#1974, NIH AIDS Reagent Program, Germantown, MD) (Supplemental Figure 1B). The endotoxin was removed using an EndoTrap HD (Bio Vendor, Asheville, NC) and determined below the detection limit (<0.05 EU/μg) by E-TOXATE Kit (MilliporeSigma, Burlington, MA).

### Animals

C57BL/6J wild-type (WT) mice were purchased from The Jackson Laboratory (Bar Harbor, ME). Myeloid-specific IKKβ knockout (IKKβ^ΔMye^) mice on C57BL/6J background were generated by crossing mice carrying loxP-flanked IKKβ alleles (IKKβ^F/F^) with LysM-Cre transgenic mice [39], as previously described [26]. To increase susceptibility to atherosclerotic lesion development, the IKKβ^ΔMye^ mice were crossed to LDLR^−/−^ mice (Jackson Laboratories) to generate IKKβ^ΔMye^LDLR^−/−^ and IKKβ^F/F^LDLR^−/−^ mice [26]. All experimental mice had IKKβ^F/F^LDLR^−/−^ double-mutant background, and IKKβ^ΔMye^LDLR^−/−^ mice carried heterozygous knock-in for LysM-Cre. All experimental mice used in this study were male, partially due to the known crosstalk between NF-κB and estrogen signaling [40, 41]. However, the authors are aware of the fact that studying a single sex has limitations since sex differences have been widely reported in mouse atherosclerosis studies [42].

For the Tat treatment, 8-week-old male C57BL/6J mice on chow diet were intravenously injected with HIV protein Tat (250 or 1000 ng/mice), PBS (Control), or Placebo solution (with all the same components except for the Tat proteins) every other day for 2 weeks. For the atherosclerosis study, 6-week-old experimental male IKKβ^F/F^LDLR^−/−^ and IKKβ^ΔMye^LDLR^−/−^ littermates on a low-fat and low-cholesterol semi-synthetic AIN76a diet [43, 44] were intravenously injected with HIV protein Tat 1000 ng/mice twice/week for 12 weeks until euthanasia at 18 weeks of age. Body composition was measured by EchoMRI (Echo Medical System), and intraperitoneal glucose tolerance test (GTT) was performed as previously described [21, 45]. On the day of euthanasia, mice were fasted for 6 hours following the dark cycle (feeding cycle), and blood and tissues were then collected as described previously [29, 26, 46]. Plasma total cholesterol and triglyceride concentrations were determined enzymatically by colorimetric methods as described previously [47, 48]. All animals were housed in a pathogen-free environment with a 12 hour light-dark cycle under an approved protocol.

### Atherosclerotic lesion analysis

The atherosclerotic lesions were quantified as previously described [45, 26]. To quantify the lesion areas at the aortic root, Optimal Cutting Temperature (OCT)-compound-embedded hearts were sectioned at a 12 μm thickness keeping all the three valves of the aortic root in the same plane, and stained with Oil red O as described before [45, 26]. To quantify atherosclerotic lesions at the brachiocephalic artery (BCA), the OCT-embedded brachiocephalic arteries were sectioned from distal to proximal at a thickness of 10 μm. Atherosclerotic lesions lumenal to the internal elastic lamina were quantified in three equidistant oil red O-stained sections 200, 400 and 600 μm proximal from the branching point of the brachiocephalic artery into the carotid and subclavian arteries [45, 26].

### Atherosclerotic plaque morphological histomorphometric analysis

Atherosclerotic plaques at the aortic root were sectioned, as described previously [26, 45]. Plaque morphological histomorphometric characters were analyzed by hematoxylin and eosin staining [46, 29]. Plaque composition of lipid-rich cores, collagen, SMCs, and macrophage contents were analyzed by Oil Red O staining, trichrome staining, immunofluorescence staining for α-smooth muscle actin (αSMA) and CD68, respectively. Plaque stability was evaluated by comparing the ratios of the plaque components mentioned above with the entire plaques. The histological plaque stability score was also calculated, as described previously, following the following formula: (plaque stability score) = (SMC area + collagen area) / (macrophage area + lipid area) [49, 29].

### Macrophage isolation and function assays

Macrophages were isolated as previously described [26, 46]. Bone marrow-derived macrophages (BMMs) were isolated from the femurs of mice and cultured in DMEM medium supplemented with 10 ng/mL recombinant mouse macrophage colony-stimulating factor (Invitrogen, Waltham, MA) for 7 days before the experiment. Peritoneal macrophages (PMs) were harvested from each genotype by peritoneal lavage with PBS after 12-week intravenous injection of HIV protein Tat (1000 ng/mice twice/week). For adhesion assays, calcein acetoxymethyl-labeled PMs were incubated with primary porcine endothelial cells. The attached cells were fixed and counted under the microscope [26, 46]. Migration assays were performed using transwells with 8.0 μm pore polycarbonate membrane inserts (Corning Inc., Corning, NY). Macrophages in serum-free media were seeded on the transwell filters (top chambers), and the lower chambers were filled with the complete media including 10% serum as a chemoattractant [45]. After 16 hours, the cells were removed from the upper surface of the insert using Q-Tips. The membranes were fixed with 1% glutaraldehyde (MilliporeSigma, St. Louis, MO), stained with hematoxylin (Leica, Wetzlar, Germany), and mounted on the slides using glycerol gelatin (MilliporeSigma, St. Louis, MO). Hematoxylin-stained cells were counted under the microscope.

### RNA Isolation and Quantitative Real-Time PCR Analysis (QPCR)

Total RNA was isolated from mouse tissues or cells using TRIzol Reagent (Thermo Fisher Scientific, Waltham, MA), and QPCR was performed using gene-specific primers and the SYBR Green PCR kit (Bio-Rad Laboratories, Hercules, CA) as previously described [45]. The sequences of primer sets used in this study are listed in Supplemental Table 1.

### RNA sequencing and data analysis

PMs were isolated from IKKβ^F/F^LDLR^−/−^ and IKKβ^ΔMye^LDLR^−/−^ mice 4 days after the peritoneal injection of 1 mL 3% thioglycollate. Cells were attached to cell culture plates for 4 hours and were then treated with PBS (Control) or HIV protein Tat (100 nM) for 12 hours. Total RNA was extracted, and RNA integrity was confirmed using a dual Agilent 2100 Bioanalyzer (Agilent Technologies Inc., Santa Clara, CA). The creation of cDNA libraries and sequencing were performed using the Illumina standard operation pipeline as previously described [50, 51, 45]. For data analysis, we applied the *Salmon* tool [52] to quantify the mRNA expression from the raw sequencing data, using the *Ensembl* [53] mouse gene annotation (GRCm38). Transcript per million reads (*TPM*) was used as the unit of mouse gene expression level. We then used the *edgeR* algorithm [54] to compare the groupwise transcriptomic pattern. We also applied the *TMM* algorithm implemented in the *edgeR* package to perform reads count normalization and effective library size estimation. Group-wise differential expression was estimated by the likelihood ratio test included in the *edgeR* package. The genes with a false discovery rate (*FDR*) < 1% and fold change (*FC*) >3 were deemed differentially expressed. All RNAseq datasets have been deposited in the Gene Expression Omnibus (GSE157833). We further performed gene ontology analysis upon the differentially expressed genes using the definition from the Gene Ontology (GO) [55] and Kyoto Encyclopedia of Genes and Genomes (KEGG) [56] projects. For each GO Biological Process term or KEGG pathway, we computed a geneset score, using the Functional Analysis of Individual Microarray Expression (*FAIME*) algorithm [57]. Briefly, FAIME computes geneset scores using rank-weighted gene expression data of individual samples, which converts each sample’s genome-wide gene expression profile into molecular mechanisms [57]. A higher geneset score indicates higher overall expression of a given GO term or KEGG pathway.

### Western blotting

Western blotting was performed as previously described [47, 48]. Briefly, the cells or tissues were homogenized with Bullet Blender (Next Advance, Averill Park, NY) in 0.5 mL of ice-cold lysis buffer (Cell Signaling Technology, Danvers, MA) containing protease inhibitor cocktails (Roche, Basel, Switzerland). After homogenization, lysates were centrifuged at 16,000 × g for 15 min at 4 °C to collect the supernatant. Protein concentrations were measured using a BCA protein assay kit (Thermo Fisher Scientific, Waltham, MA). Proteins were resolved on SDS-PAGE, and then electro-transferred onto nitrocellulose membrane. The membrane was blocked in phosphate buffered saline solution with 0.05% Tween 20 (PBST, pH 7.4) containing 5 % non-fat dry milk (Bio-Rad Laboratories, Hercules, CA) for 1 hour, and then incubated with anti-IKKβ antibodies (1:500, Cell signaling Technology, Danvers, MA) in PBST containing 5% BSA at 4°C overnight. After the incubation, the membrane was washed four times with PBST, and incubated with HRP-conjugated secondary antibodies (1:2000; MilliporeSigma, St. Louis, MO) in PBST with 5 % non-fat dry milk for 1 hour at room temperature. After subsequent washing in PBST 3 times, the membrane was washed once in PBS and developed using Pierce ECL Western Blotting Substrate (Thermo Fisher Scientific, Waltham, MA) followed by exposure to CL-XPosure films (Thermo Fisher Scientific, Waltham, MA) for visualization of protein bands.

### Immunohistochemistry

Immunohistochemistry was performed on 12-μm sections of aortic roots freshly embedded in OCT. The slides were fixed in ice-cold acetone for 15 min and permeabilized with PBS + 0.1% Triton X-100 (PBST) for 15 min and blocked with PBST containing 5% BSA (MilliporeSigma, St. Louis, MO) for 1 hour at room temperature. The sections were then incubated with antibodies against CD68 antibody (1:100; Bio-Rad Laboratories, Hercules, CA), interleukin 6 (IL-6, 1:100; Bio-Rad Laboratories, Hercules, CA), αSMA antibody (1:100; Abcam, Cambridge, United Kingdom), or tumor necrosis factor α (TNFα; 1:100; Abcam, Cambridge, United Kingdom) at 4°C overnight. The slides were rinsed with PBS and incubated with corresponding secondary antibodies (1:500; Life Technologies, Carlsbad, CA). The nuclei were stained by mounting the slides with 4’, 6-diamidino-2-phenylindole (DAPI) medium (Vector Laboratories, Burlingame, CA). Images were acquired under a Nikon fluorescence microscope (Nikon, Melville, NY). For collagen staining, Masson’s Trichrome staining was performed following a previously published procedure [58].

### Statistical Analysis

All data except the high-throughput sequencing data are presented as the mean ± SEM. Individual pairwise comparisons were analyzed by two-sample, two-tailed Student’s t-test using GraphPad Prism unless otherwise noted. A p<0.05 was regarded as significant. Two-way ANOVA was performed when multiple comparisons were made followed by a Bonferroni multiple comparisons test. Sample numbers (n) are provided in the respective figure legends.

## Results

### HIV protein Tat elicits inflammatory responses in macrophages of wild type mice

**Figure 1.**
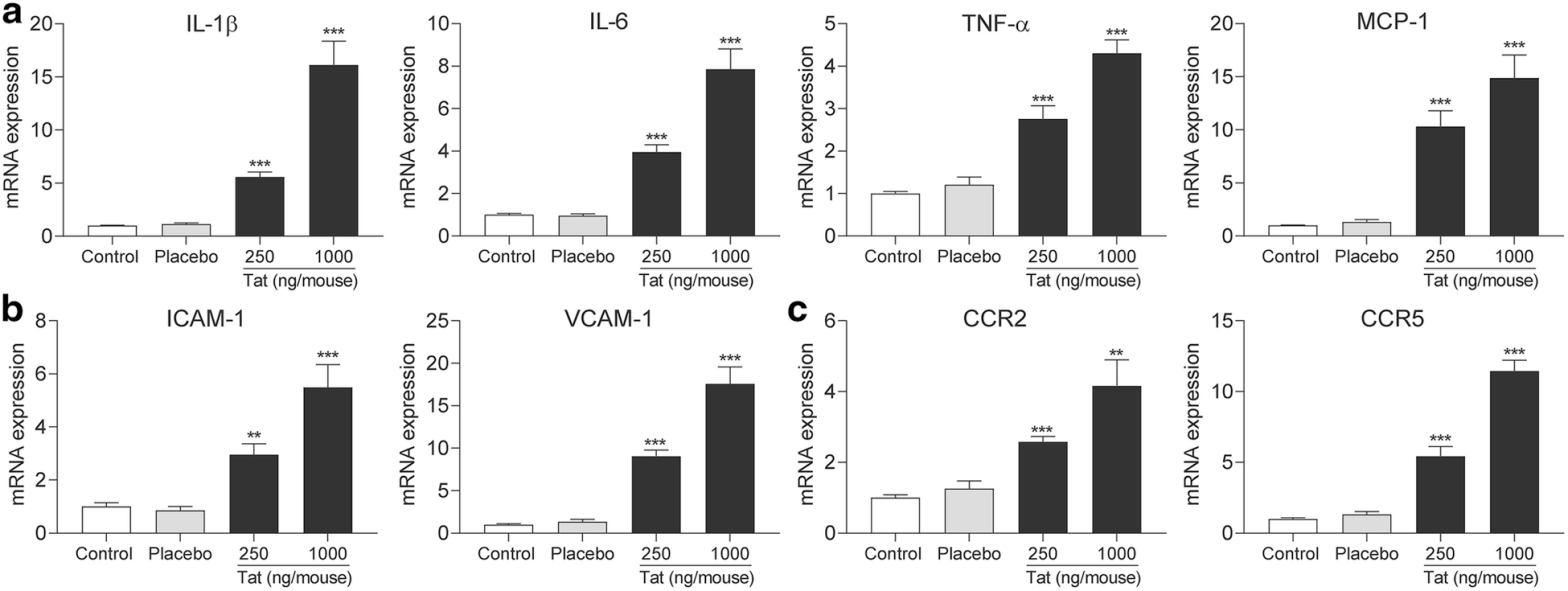
HIV protein Tat induces inflammatory responses in macrophages of wild-type mice. Eight-week-old male wild-type mice were treated with vehicle control (PBS), placebo solution, 250, or 1000 ng recombinant Tat proteins by intravenous injection every other day for 2 weeks. Total RNA was extracted from freshly isolated peritoneal macrophages, and the expressions of inflammatory cytokines (A), adhesion molecules (B), and chemokines (C) were analyzed by QPCR (n=5, **P<0.01 and ***P<0.001).

Atherosclerosis is an inflammatory disease, and macrophages are one of the major cell types contributing to the atherosclerotic initiation and progression [32, 59]. To investigate the impact of recombinant HIV Tat proteins on macrophage inflammatory responses in vivo, WT mice were intravenously injected with PBS control, placebo solution, or Tat proteins at doses of 250 and 1000 ng every other day for 2 weeks. Peritoneal macrophages were then isolated and the mRNA levels of proinflammatory cytokines were measured. Consistent with previous in vitro studies [17, 16, 15], the expression levels of several key inflammatory cytokines, including IL-1β, IL-6, TNFα and monocyte chemotactic protein (MCP)-1, were significantly increased by Tat protein treatment in a dose-dependent manner (Figure 1A). Furthermore, exposure to HIV Tat proteins also increased the expression of adhesion molecules (Figure 1B) and chemokines (Figure 1C) in the macrophages of WT mice. There is no significant difference on the expression of those genes between placebo and PBS treatment. These results suggest that exposure to recombinant HIV protein Tat is sufficient to induce macrophage inflammatory responses in vivo.

### Deficiency of myeloid IKKβ does not affect the impact of HIV Tat exposure on metabolic phenotypes and plasma lipid profiles of LDLR^−/−^ mice

To study the functions of macrophage IKKβ in atherosclerosis, we previously generated LDLR^−/−^ mice with myeloid-specific IKKβ deficiency (IKKβ^ΔMye^LDLR^−/−^) mice by crossing IKKβ^ΔMye^ (LysM-Cre/IKKβ^F/F^) mice with LDLR^−/−^ mice [26]. As expected, the mRNA and protein levels of IKKβ were significantly decreased in PMs and BMMs but not in other major tissues of IKKβ^ΔMye^LDLR^−/−^ mice as compared with IKKβ^F/F^LDLR^−/−^ littermates (Supplementary Figure 2).

To determine whether IKKβ mediates the atherogenic effects of Tat proteins in vivo, 6-week-old male IKKβ^ΔMye^LDLR^−/−^ and IKKβ^F/F^LDLR^−/−^ littermates were treated intravenously with recombinant Tat proteins (1000 ng/mouse) twice a week for 12 weeks. All mice used in this study had IKKβ^F/F^LDLR^−/−^ double-mutant background, and IKKβ^ΔMye^LDLR^−/−^ mice also carried heterozygous knock-in for LysM-Cre. The mice were also fed a low-fat AIN76 diet containing 4.2% fat and 0.02% cholesterol, which has been successfully used in many studies to induce atherosclerosis in LDLR^−/−^ or ApoE^−/−^ mice without eliciting obesity and associated metabolic disorders [43, 60, 45].

Exposure to Tat proteins did not affect body weight and composition, including fat mass and lean mass of IKKβ^F/F^LDLR^−/−^ and IKKβ^ΔMye^LDLR^−/−^ mice (Supplementary Figure 3A). IKKβ^F/F^LDLR^−/−^ and IKKβ^ΔMye^LDLR^−/−^ mice also had similar fasting blood glucose levels, and glucose tolerance tests demonstrated that myeloid IKKβ-deficiency or Tat treatment did not alter glucose tolerance in IKKβ^ΔMye^LDLR^−/−^ mice (Supplementary Figure 3B). Next, we measured the plasma lipid levels and found that Tat treatment did not affect plasma cholesterol levels in IKKβ^F/F^LDLR^−/−^ and IKKβ^ΔMye^LDLR^−/−^ mice. While Tat treatment elevated plasma triglyceride levels in both IKKβ^F/F^LDLR^−/−^ and IKKβ^ΔMye^LDLR^−/−^ mice, deficiency of IKKβ did not affect the impact of Tat protein treatment on triglyceride levels (Supplementary Figure 3C).

### HIV protein Tat increases atherosclerosis in IKKβ^F/F^LDLR^−/−^ but not IKKβ^ΔMye^LDLR^−/−^ mice

**Figure 2.**
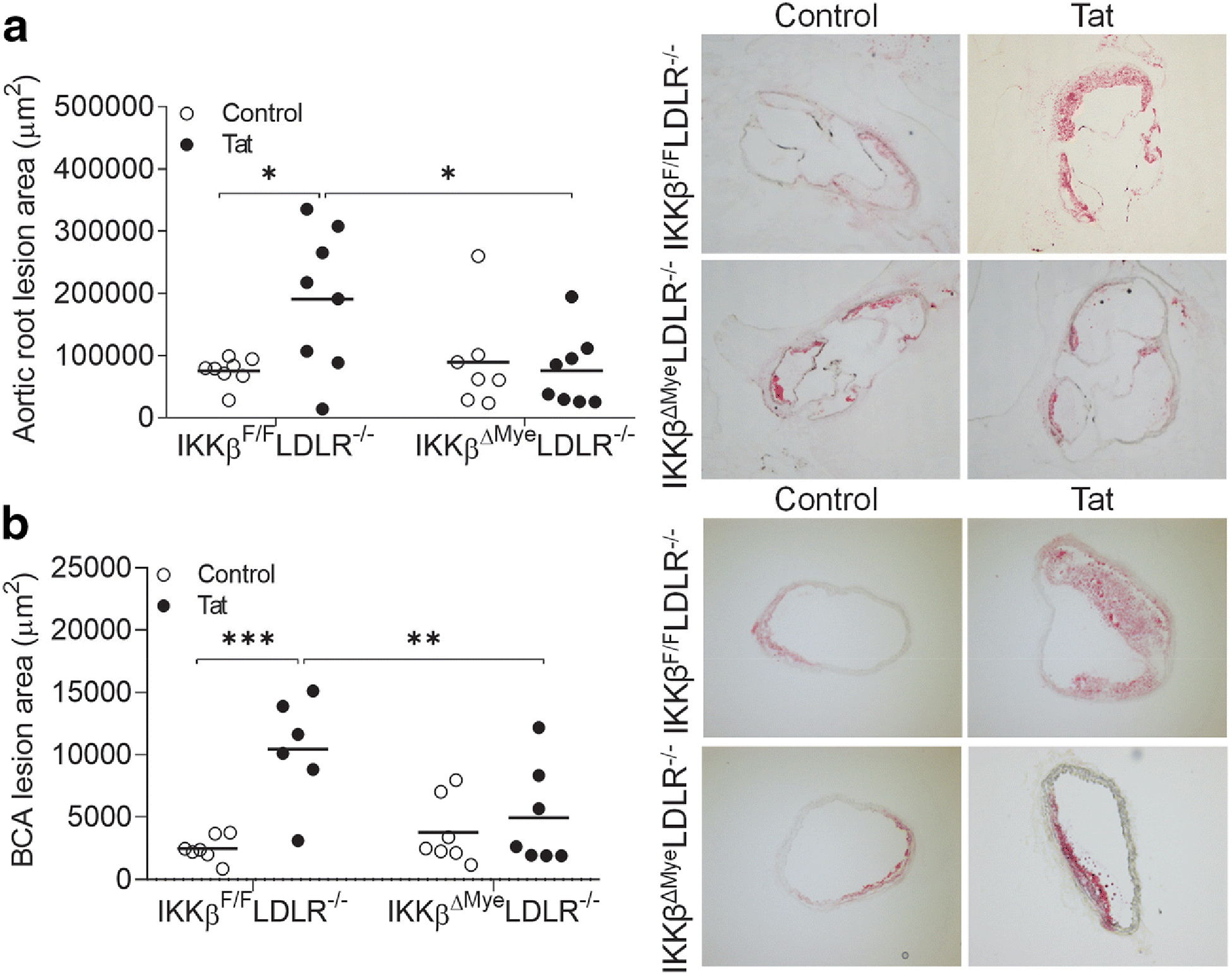
HIV protein Tat increases atherosclerosis in IKKβ^F/F^LDLR^−/−^ but not IKKβ^ΔMye^LDLR^−/−^ mice Six-week-old male IKKβ^F/F^LDLR^−/−^ and IKKβ^ΔMye^LDLR^−/−^ littermates were fed a semi-synthetic AIN76a diet containing 0.02% cholesterol and treated with vehicle control or 1000 ng of recombinant Tat proteins intravenously twice a week for 12 weeks. Quantitative analysis of the atherosclerotic lesion area at the aortic root (A) and brachiocephalic artery (BCA) (B) of IKKβ^F/F^LDLR^−/−^ and IKKβ^ΔMye^LDLR^−/−^ mice (n=6–8, *P<0.05, **P<0.01 and ***P<0.001). Representative images of Oil-red-O-stained sections from each genotype are displayed on the right side of the quantification data.

Although Tat treatment had similar effects on metabolic phenotypes and plasma lipid levels in IKKβ^F/F^LDLR^−/−^ and IKKβ^ΔMye^LDLR^−/−^ mice, quantification of cross-sectional lesion areas at the aortic root revealed that exposure to Tat proteins significantly increased the lesion sizes by 153.6% (Figure 2A, 190753.8 ± 39993.2μm^2^ vs. 75225.8 ± 7736.4μm^2^) in IKKβ^F/F^LDLR^−/−^ mice but not in IKKβ^ΔMye^LDLR^−/−^ mice (Figure 2A, 75521.4 ± 20915.9μm^2^ vs. 89259.0 ± 30370.9μm^2^). Consistently, Tat treatment also increased the lesional areas at the brachiocephalic artery (BCA) of IKKβ^F/F^LDLR^−/−^ mice by 320.2% (Figure 2B, 10448.8 ± 1746.0μm^2^ vs. 2486.6 ± 377.6μm^2^) but deficiency of myeloid IKKβ abolished the impact of Tat proteins on atherosclerosis development in the BCA of IKKβ^ΔMye^LDLR^−/−^ mice (Figure 2B, 4940.9 ± 1520.5μm^2^ vs. 3777.7 ± 997.4μm^2^). These results demonstrated that exposure to HIV protein Tat leads to increased atherosclerosis in LDLR^−/−^ mice, and that myeloid IKKβ signaling may contribute to Tat’s atherogenic effects in vivo.

### HIV protein Tat induces atherosclerotic lesional inflammation and plaque vulnerability in LDLR^−/−^ mice

**Figure 3.**
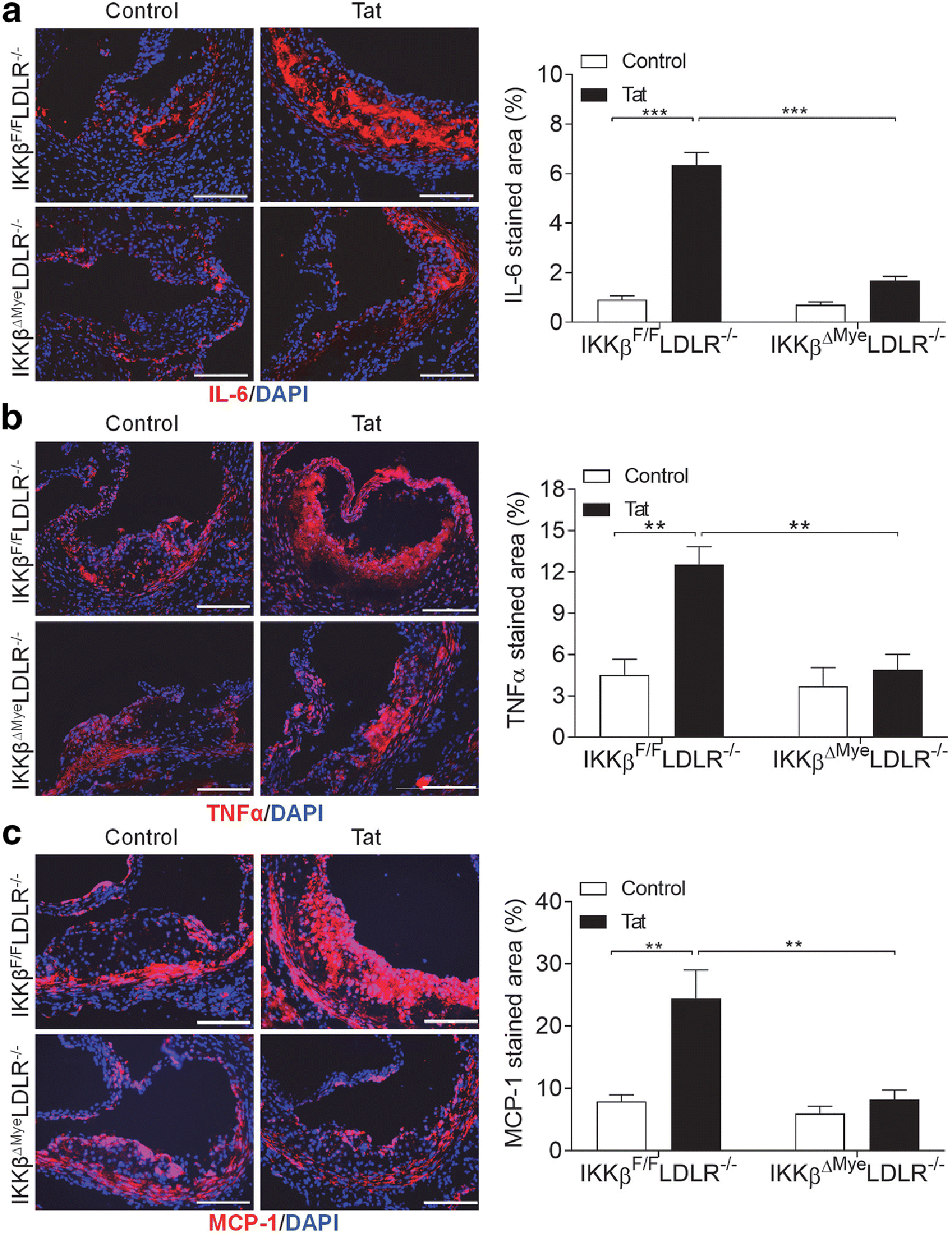
Deficiency of myeloid IKKβ reduces HIV Tat-induced atherosclerotic lesional inflammation in LDLR^−/−^ mice. Six-week-old male IKKβ^F/F^LDLR^−/−^ and IKKβ^ΔMye^LDLR^−/−^ littermates were treated with vehicle control or 1000 ng of recombinant Tat proteins intravenously twice a week for 12 weeks. Representative images (left) and quantification (right) of IL-6 (A), TNFα (B) and MCP-1 (C) immunofluorescence staining at aortic roots of IKKβ^F/F^LDLR^−/−^ and IKKβ^ΔMye^LDLR^−/−^ mice (n=3, **P<0.01 and ***P<0.001. Scale bar, 100 μm). DAPI indicates 4’, 6-diamidino-2-phenylindole.

We have previously demonstrated the important role of IKKβ in regulating atherosclerosis lesional inflammation [26, 21]. Immunofluorescence staining showed that the expression levels of several key proinflammatory proteins including IL-6, TNFα and MCP-1 were significantly increased in both atherosclerotic lesions and vessel walls by Tat treatment at the aortic root of IKKβ^F/F^LDLR^−/−^ but not IKKβ^ΔMye^LDLR^−/−^ mice (Figure 3).

**Figure 4.**
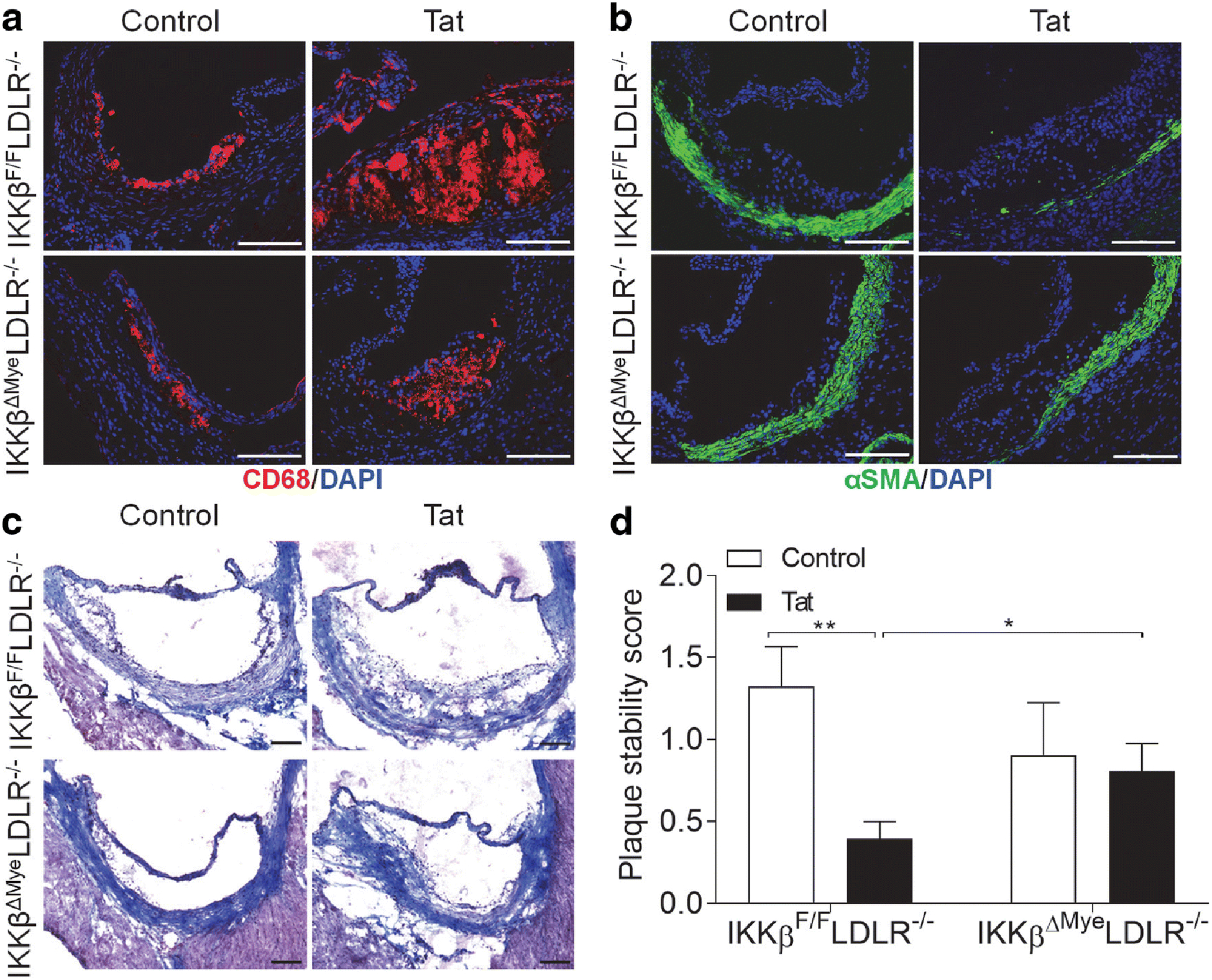
Deficiency myeloid IKKβ decreases HIV Tat-induced atherosclerotic plaque vulnerability in LDLR^−/−^ mice. Six-week-old male IKKβ^F/F^LDLR^−/−^ and IKKβ^ΔMye^LDLR^−/−^ littermates were treated with vehicle control or 1000 ng of recombinant Tat proteins intravenously twice a week for 12 weeks. Representative images of immunofluorescence staining of CD68 (A) and αSMA (B) and trichrome staining (C) at the aortic roots of IKKβ^F/F^LDLR^−/−^ and IKKβ^ΔMye^LDLR^−/−^ mice (Scale bars, 100 μm). Analysis of plaque stability scores (D) at aortic roots of IKKβ^F/F^LDLR^−/−^ and IKKβ^ΔMye^LDLR^−/−^ mice (n=5, *P<0.05 and **P<0.01). DAPI indicates 4’, 6-diamidino-2-phenylindole.

Enhanced inflammation may also lead to increased atherosclerotic plaque vulnerability, which was characterized by the combination of increased lipid-rich necrotic core size, decreased thickness of the fibrous cap, decreased plaque collagen and SMC content, and increased macrophage contents [29, 61, 49]. As expected, immunostaining for macrophage and SMC markers showed that macrophage content was increased (Figure 4A), but the SMC contents were decreased (Figure 4B) in the atherosclerotic lesions of IKKβ^F/F^LDLR^−/−^ mice after Tat treatment. Further, trichrome staining showed that exposure to Tat proteins also led to increased collagen contents in the atherosclerotic lesion of IKKβ^F/F^LDLR^−/−^ mice (Figure 4C). However, deficiency of myeloid IKKβ abolished the impact of Tat treatment on macrophages, SMC, and collagen contents in atherosclerotic lesions of IKKβ^ΔMye^LDLR^−/−^ mice (Figure 4, A–C). The contents of macrophages, SMCs, and collagens in the atherosclerotic lesions were quantified, and the histological plaque stability scores were calculated and confirmed that exposure to Tat protein led to significantly decreased plaque stability scores in IKKβ^F/F^LDLR^−/−^ mice but not in IKKβ^ΔMye^LDLR^−/−^ mice (Figure 4D). Collectively, HIV Tat exposure led to increased atherosclerotic lesional inflammation and plaque vulnerability in IKKβ^F/F^LDLR^−/−^ but not IKKβ^ΔMye^LDLR^−/−^ mice.

### Deficiency of IKKβ ameliorates Tat-elicited macrophage dysfunction

**Figure 5.**
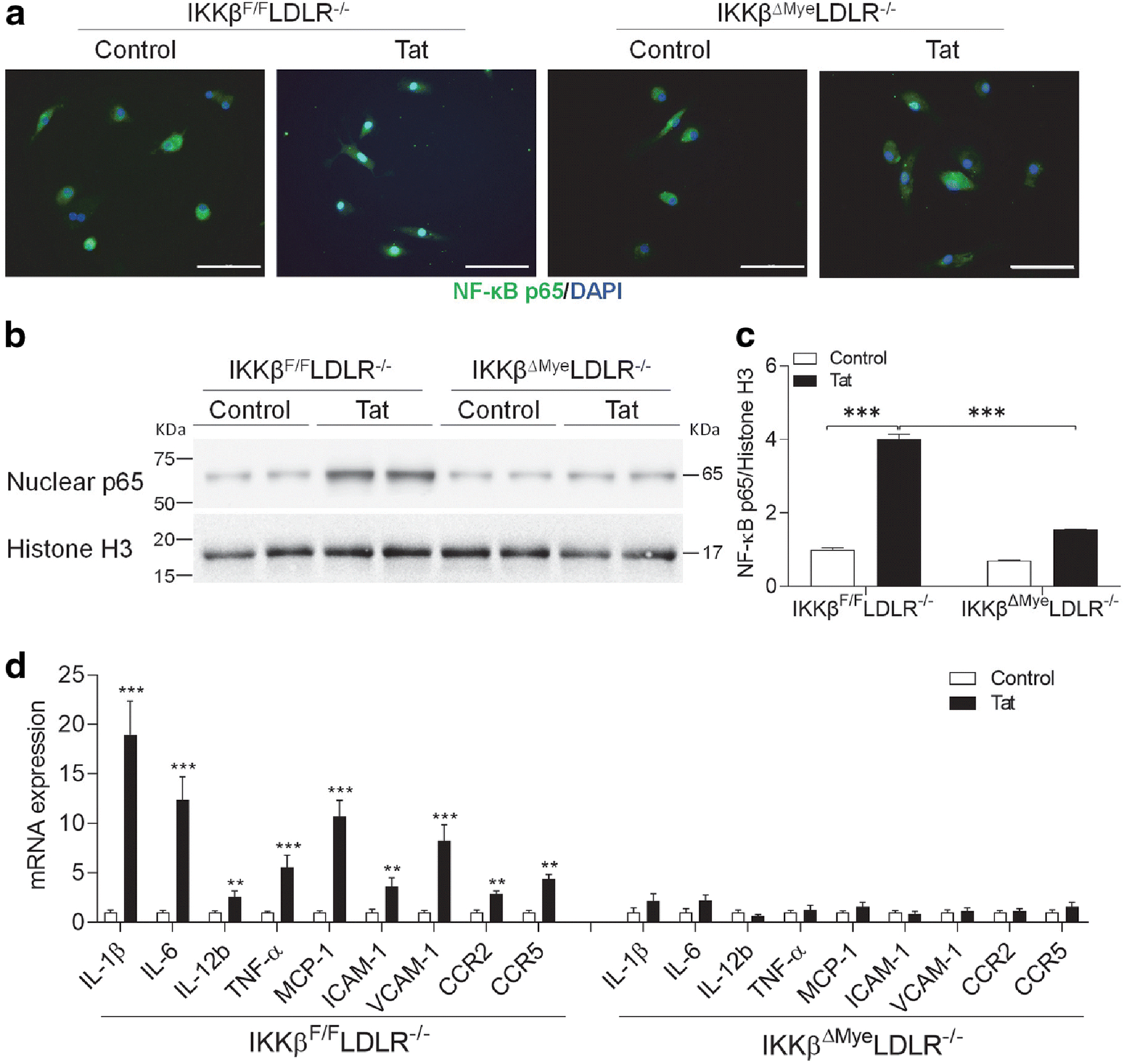
Ablation of IKKβ suppresses Tat-stimulated NF-κB activation and inflammatory responses in macrophages. (A) Bone marrow-derived macrophages were isolated from IKKβ^F/F^LDLR^−/−^ and IKKβ^ΔMye^LDLR^−/−^ mice and treated with 200 nM Tat or vehicle for 30 minutes. Cells were then fixed and stained with anti-NF-κB p65 primary antibody, followed by fluorescein-labeled secondary antibody. The nuclei were visualized with 4’, 6-diamidino-2-phenylindole (DAPI). A representative figure from 3 independent experiments with similar results is shown (Scale bar, 100 μM). (B) Nuclear proteins were extracted, and NF-κB p65 levels was analyzed by immunoblotting. (C) QPCR analysis of fresh isolated peritoneal macrophages of IKKβ^F/F^LDLR^−/−^ and IKKβ^ΔMye^LDLR^−/−^ mice treated with vehicle control or 1000 ng of recombinant Tat proteins for 12 weeks (n=6, **P<0.01 and ***P<0.001).

IKKβ is required for the activation of canonical or classical NF-κB, which regulates many inflammatory signaling pathways that contribute to atherosclerosis initiation and progression. To determine the role of IKKβ in Tat regulated macrophage inflammatory responses, BMMs isolated from IKKβ^ΔMye^LDLR^−/−^ and IKKβ^F/F^LDLR^−/−^ mice were treated with Tat proteins. Tat-induced NF-κB subunit p65 translocation from cytoplasm to nucleus was inhibited in BMMs of IKKβ^ΔMye^LDLR^−/−^ mice (Figure 5A), which was confirmed by western blot analysis of nucleic NF-κB subunit p65 (Figure 5B). These results confirmed the important role of IKKβ in mediating HIV protein Tat-induced NF-κB activation in macrophages.

Atherosclerosis is an inflammatory disease and macrophages are the major inflammatory cells contributing to atherosclerotic lesion formation and progression[32, 62]. Gene profiling of freshly isolated PMs from control and Tat-treated IKKβ^F/F^LDLR^−/−^ and IKKβ^ΔMye^LDLR^−/−^ revealed that exposure to Tat induced the expression of key proinflammatory genes including IL-1β, IL-6, IL12b, TNFα, MCP-1 in macrophage IKKβ^F/F^LDLR^−/−^ mice. In addition, several chemokines and adhesion molecules including CCR2, CCR5, vascular cell adhesion molecule (VCAM)-1 and intercellular adhesion molecule (ICAM)-1 were also upregulated by Tat treatment in macrophages from IKKβ^F/F^LDLR^−/−^ mice. Consistently, deficiency of IKKβ in macrophages was able to abolish this induction (Figure 5C).

**Figure 6.**
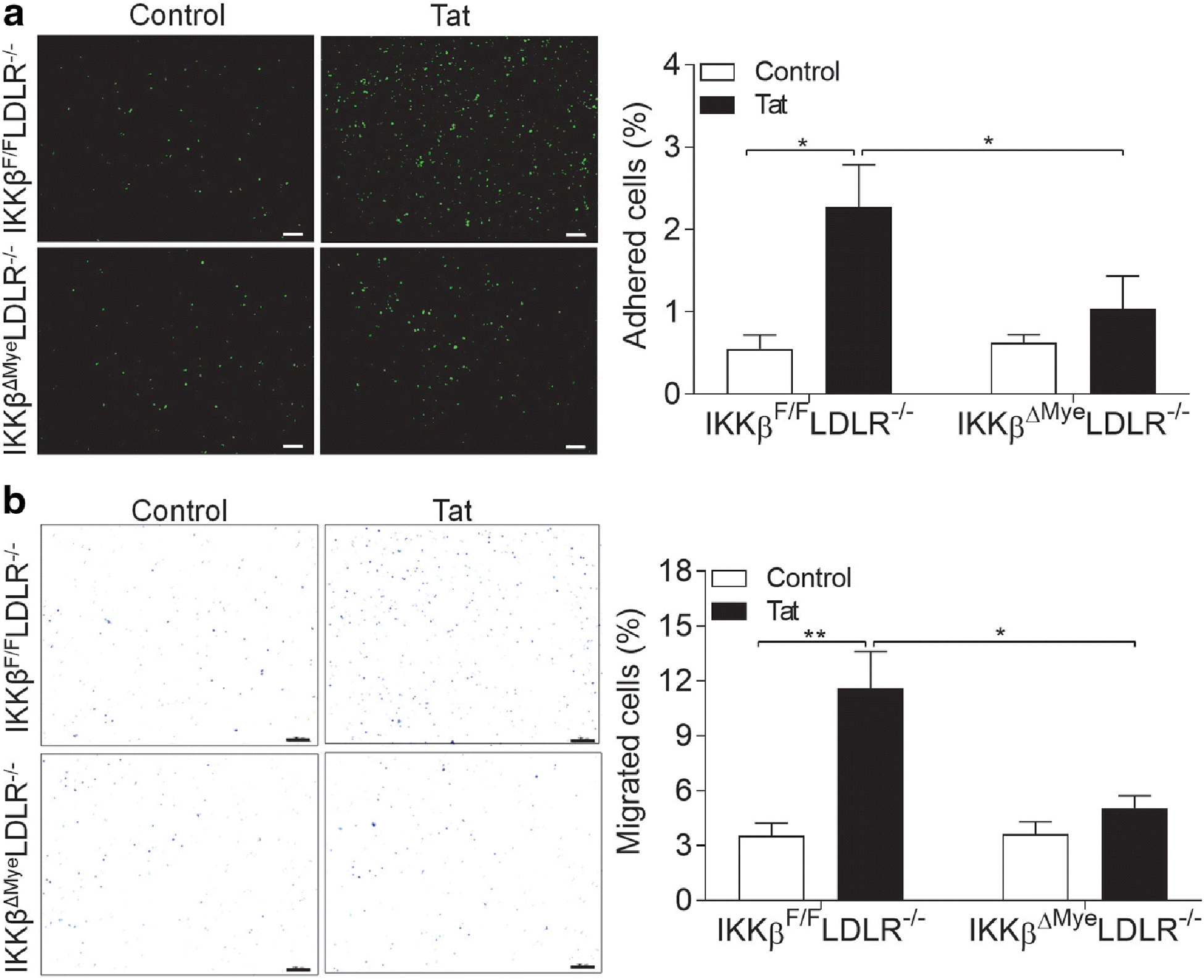
Deficiency of IKKβ in macrophages ameliorates Tat-elicited macrophage adhesion and migration. Peritoneal macrophages were freshly isolated from IKKβ^F/F^LDLR^−/−^ and IKKβ^ΔMye^LDLR^−/−^ mice treated with vehicle control or 1000 ng of recombinant Tat proteins for 12 weeks. (A) Macrophages were labeled with calcein acetoxymethyl and cultured with primary porcine endothelial cell monolayer for 30 minutes. Adhered cells were then counted under a fluorescence microscope. (B) Macrophages were seeded on the transwell filters. Cells that infiltrated and migrated to the underside of transwell were stained with hematoxylin and counted under the microscope. Quantification data is presented in the right panels (n=5, *P<0.05 and **P<0.01. Scale bar, 100 μm).

One of the earliest events in atherogenesis is the entry of monocytes, the precursors of macrophages, into the artery wall. Since HIV protein Tat can affect several NF-κB-regulated chemokines and adhesion molecules in macrophages of IKKβ^F/F^LDLR^−/−^ mice (Figure 5C), we next isolated PMs from control and Tat-treated IKKβ^F/F^LDLR^−/−^ and IKKβ^ΔMye^LDLR^−/−^ mice to investigate adhesion properties of those macrophages. Incubation of freshly isolated PMs with primary endothelial cells (ECs) showed that Tat treatment increased adhesion of control but not IKKβ-deficient macrophages to ECs (Figure 6A). We also examined the effects of Tat exposure on macrophage migration using a transwell assay. As shown in Figure 6B, treatment with Tat stimulated the migration of macrophages isolated from IKKβ^F/F^LDLR^−/−^ mice and this induction was abolished in macrophages from IKKβ^ΔMye^LDLR^−/−^ mice. Therefore, deficiency of IKKβ decreased HIV protein Tat-elicited macrophage adhesion and migration.

### HIV protein Tat affects the expression of many atherosclerosis-related genes in macrophages in vitro

**Figure 7.**
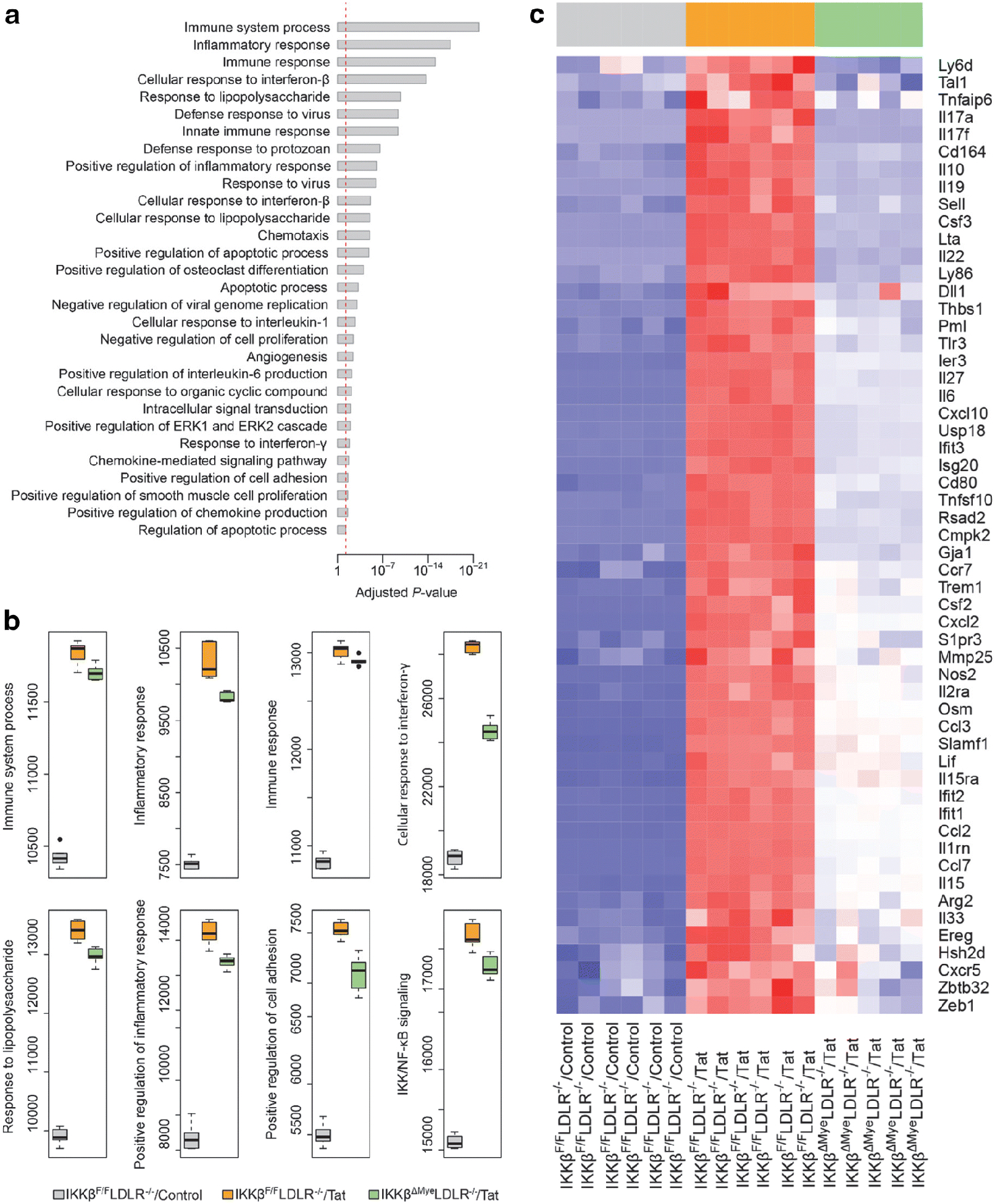
HIV protein Tat affects the expression of many atherosclerosis-related genes in microphages in vitro. Peritoneal macrophages were isolated from IKKβ^F/F^LDLR^−/−^ and IKKβ^ΔMye^LDLR^−/−^ mice. Cells were treated with 100 nM Tat or vehicle control for 12 hours and total RNA was isolated for RNAseq analysis (n = 5–6). (A) The Gene Ontology (GO) Biological Process terms significantly associated with the DEGs in control macrophages after HIV Tat treatment. The *P*-values were computed by *Fisher*’s exact test. The vertical dash line indicates the significance level of *α*=0.01. The y-axis displays the GO Biological Process terms while the x-axis displays the *P*-values. (B) Geneset scores of the prioritized GO terms. The geneset score was calculated using the FAIME algorithm. (C) Heatmap representation of DEGs involved in the biological processes of “immune system process”, “inflammatory response”, “immune response”, “cellular response to interferon β”, “response to lipopolysaccharide”, “positive regulation of inflammatory response”, “positive regulation of cell adhesion” and “IKK/NF-κB signaling” shown in panel A and B. Each row shows one individual gene and each column a biological replicate of mouse. Red represents relatively increased gene expression while blue denotes downregulation.

**Figure 8.**
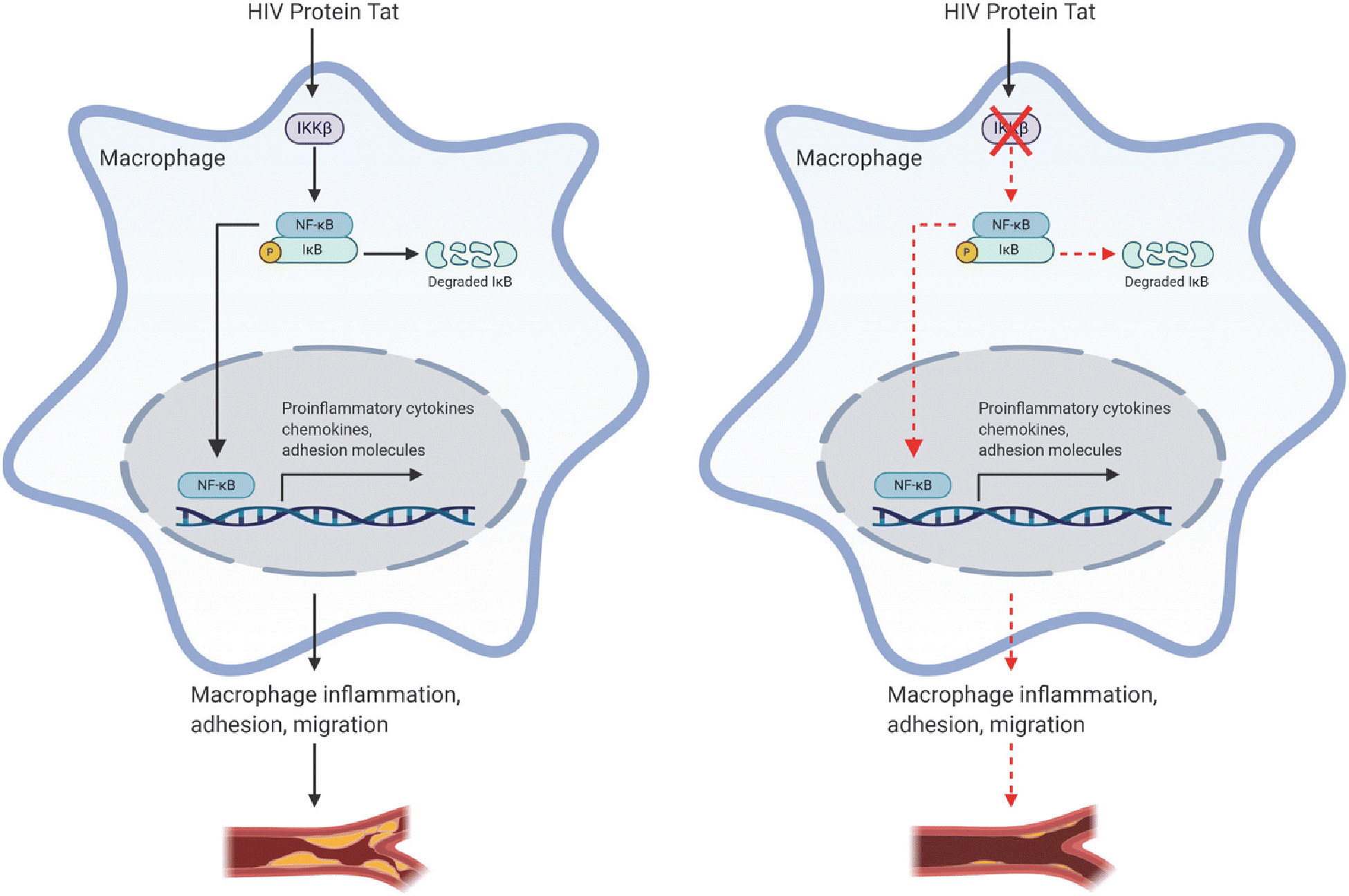
Schematic representation of the role of IKKβ in mediating HIV protein Tat-elicited macrophage dysfunction and atherosclerosis. Exposure to Tat protein activates IKKβ/NF-κB signaling in macrophages, leading to increased macrophage dysfunction and atherosclerosis development in atherogenic LDLR^−/−^ mice. Deficiency of myeloid IKKβ inhibits Tat-induced atherosclerosis in LDLR^−/−^ mice, likely due to reduced macrophage inflammation and dysfunction.

To further understand the role of IKKβ signaling in mediating HIV protein Tat-regulated macrophage transcriptome related to atherogenesis, PMs were isolated from IKKβ^F/F^LDLR^−/−^ and IKKβ^ΔMye^LDLR^−/−^ mice and then treated with control and Tat proteins for RNA-Seq analysis. GO analysis showed that Tat treatment significantly induced more than 2600 differentially expressed genes (DEGs) in macrophages of IKKβ^F/F^LDLR^−/−^ mice with a false discovery rate (*FDR*) of <1% and a fold change (*FC*) >3 as a cut-off threshold (Supplemental Table 2). In addition to the genes we checked by QPCR (e.g. IL-1β, IL-6, IL-12b, MCP-1, VCAM-1), many other known NF-κB target genes, including NLRP3, IL-1α, IL-12α, IL-17α, IL-23α, CCL5, and IFNγ, were also upregulated by Tat treatment in macrophages (Supplemental Table 2). As expected, those DEGs were enriched in several biological processes that may contribute to atherogenesis including immune system process, inflammatory response, and positive regulation of cell adhesion (Figure 7A). FAIME analysis demonstrated higher geneset scores of those GO terms in the Tat-treated macrophages from IKKβ^F/F^LDLR^−/−^ mice compared with the controls (Figure 7B). As expected, IKKβ deletion resulted in reduced geneset scores in the macrophages of IKKβ^ΔMye^LDLR^−/−^ treated with Tat proteins (Figure 7B). For example, IKKβ ablation suppressed the Tat-induced geneset scores of IKK/NF-κB signaling, inflammatory response, and positive regulation of cell adhesion in the IKKβ-deficient macrophages (Figure 7B). In addition to GO analysis, Kyoto Encyclopedia of Genes and Genomes (KEGG) pathway analysis was also performed (Supplementary Figure 4). We found that Tat treatment can affect several other pathways including cytokine-cytokine receptor interaction, TNF signaling pathway, HIF-1 signaling pathway and Jak-STAT signaling pathway that may also be associated with HIV Tat-induced atherosclerosis (Supplementary Figure 4A). Similar, *FAIME* analysis showed that deficiency of IKKβ could reduce the geneset scores of those signaling pathways elevated by Tat treatment in control macrophages (Supplementary Figure 4B). Consistent with the results of GO analysis (Figure 7C), many of those DEGs associated with these pathways were upregulated by Tat treatment but reduced by IKKβ deficiency (Supplementary Figure 4C). Collectively, these results suggest that IKKβ signaling may mediate the impact of HIV protein Tat on multiple processes such as inflammatory response and regulation of cell adhesion that contribute to atherosclerosis initiation and development (Figure 8).

## Discussion

HIV infection has been associated with exacerbated atherosclerotic CVD in patients, but the underlying mechanisms remain poorly understood. The HIV protein Tat, a trans-activator required for efficient HIV replication [7], has been shown to activate NF-κB signaling and induce inflammatory responses in vitro [16, 36]. However, the atherogenic effects of HIV Tat had not been investigated in vivo. In the present study, we used myeloid-specific IKKβ-deficient LDLR^−/−^ mice and their control littermates to investigate the effects of HIV protein Tat on macrophage function and atherosclerosis development. We found that exposure to Tat protein led to significantly increased atherosclerotic lesion size in the aortic root and brachiocephalic artery of IKKβ^F/F^LDLR^−/−^ but not IKKβ^ΔMye^LDLR^−/−^ mice. To our knowledge, our study is the first to demonstrate the impact of HIV protein Tat on the development of atherosclerosis in an appropriate small animal model.

Atherosclerosis is a chronic inflammatory disease, and IKKβ-mediated NF-κB activation has been implicated in the pathogenesis of atherosclerosis in humans [21]. For example, activated NF-κB has been identified in atherosclerotic plaques and was enhanced in unstable coronary plaques in humans [63, 64]. Monaco et al. also found that activation of NF-κB activation in human atherosclerosis was IKKβ-dependent, leading to selective up-regulation of major proinflammatory and prothrombotic mediators [64]. IKKβ/NF-κB signaling has also been demonstrated to function in macrophages to regulate atherosclerosis development in animal models. Macrophage-specific inhibition of NF-κB by overexpressing IκBα led to decreased foam-cell formation [65]. By contrast, myeloid-specific IκBα deletion promoted atherogenesis in LDLR^−/−^ mice [66]. We have previously reported that myeloid-specific IKKβ deficiency protected LDLR^−/−^ mice from high-fat Western diet-induced macrophage inflammatory responses and atherosclerosis [26]. In the present study, we found that exposure to HIV protein Tat can induce macrophage inflammatory responses in WT mice. When fed the low-fat AIN76 diet, IKKβ^F/F^LDLR^−/−^ and IKKβ^ΔMye^LDLR^−/−^ mice had similar atherosclerosis lesion sizes under basal conditions. However, treatment with Tat stimulated macrophage inflammatory responses and atherosclerotic lesional inflammation, leading to increased atherosclerosis development in IKKβ^F/F^LDLR^−/−^ mice. Deficiency of myeloid IKKβ inhibited Tat-induced atherosclerosis in IKKβ^ΔMye^LDLR^−/−^ mice, likely due to reduced macrophage inflammation and dysfunction.

In addition to inflammatory responses, macrophage migration plays an essential role in atherosclerotic lesion initiation and progression. NF-κB can also regulate important chemokines and adhesion molecules such as ICAM-1, VCAM-1, and MCP-1[26]. In the present study, we found that exposure to HIV protein Tat also stimulated the expression of several key chemokines and adhesion molecules, including ICAM-1, VCAM-1, and CCR2 in control macrophages, which is consistent with previous studies [67, 14, 17]. Deficiency of IKKβ abolished Tat-elicited upregulation of these genes. As expected, Tat treatment led to increased adhesion and migration properties of macrophages of control IKKβ^F/F^LDLR^−/−^ mice but not IKKβ^ΔMye^LDLR^−/−^ mice. Several chemokine receptors and their ligands such as CCR2 and MCP-1 have been well-characterized to have pro-atherogenic effects in vivo, and deficiency of CCR2 also led to decreased atherosclerosis in animal models [68]. It is likely that HIV protein Tat-mediated upregulation of those molecules contribute to increased atherosclerosis in our model. Collectively, our results indicate that myeloid IKKβ signaling plays an important role in mediating the atherogenic effects of HIV protein Tat.

HIV Tat has been shown to modulate the functions of several cell types associated with atherosclerosis development, including endothelial cells, vascular smooth muscle cells and monocytes/macrophages. For example, Tat has been shown to upregulate the expression of inflammatory mediators and adhesion molecules through activating NF-κB pathway in the human vascular endothelial cells [69–71]. Several studies have also demonstrated that Tat protein can activate NF-κB pathway to induce the production of inflammatory cytokines by human monocytes/macrophages [16, 36]. In the current study, we mainly focused on the role of IKKβ/NF-κB signaling in mediating the adverse effects of Tat on macrophage functions related to atherosclerosis. In addition, several studies have also demonstrated the potential mechanisms through which Tat activates IKKβ/NF-κB signaling. Tat proteins have been shown to interact with TLR4-MD2-CD14 complex to activate NF-κB pathway and to induce the production of proinflammatory cytokines such as IL-6 and IL-8 [16, 36]. Planes et al. confirmed that HIV Tat protein could recruit the TLR4 complex with rapid kinetics, leading to the activation of downstream MyD88 and IKKβ/NF-κB signaling [36]. Therefore, the impact of HIV protein Tat on IKKβ/NF-κB signaling in macrophages is likely through activating TLR4-MyD88 signaling cascade. Future studies are required to investigate the detailed mechanisms through which Tat and other HIV proteins affect IKKβ/NF-κB signaling in different cell types including endothelial cells and smooth muscle cells to contribute to the development of atherosclerosis.

While several HIV encoded proteins are suspected to have atherogenic effects, only a few animal studies have investigated their atherogenic effects in vivo. Cui et al. previously demonstrated that HIV protein Nef could also increase macrophage foam cell formation and atherosclerosis in ApoE^−/−^ mice [72]. Interestingly, treatment with Nef also led to elevated plasma cholesterol and triglyceride levels in ApoE^−/−^ mice [72]. While Tat treatment did not affect total cholesterol levels in our study, it also increased triglyceride levels in both IKKβ^F/F^LDLR^−/−^ and IKKβ^ΔMye^LDLR^−/−^ mice. Elevated triglyceride levels have been consistently reported in HIV-infected patients including those not treated with ART [73, 74]. Nevertheless, since deficiency of myeloid IKKβ did not affect Tat-induced triglyceride levels in our study, the decreased atherosclerosis in IKKβ^ΔMye^LDLR^−/−^ mice is likely due to ameliorated macrophage functions. All experimental mice used in our atherosclerosis study were male, partially due to the known crosstalk between NF-κB and estrogen signaling [40, 41]. However, this is a limitation of our study since sex differences have been widely reported in mouse atherosclerosis studies [42].

HIV-associated atherosclerosis has also been investigated in a novel mouse model, Tg26^+/−^/ApoE^−/−^ mice [74, 75]. Tg26 mice are a well-characterized transgenic model that expresses HIV-1 [74]. Kearns et al. found that expression of HIV transcripts also accelerated atherosclerosis development in hyperlipidemic Tg26^+/−^/ApoE^−/−^ mice [74]. Interestingly, the expression of HIV-1 also led to activated inflammatory signaling such as caspase-1 in macrophages and elevated plasma inflammatory cytokine levels [74]. Caspase-1 activation is mainly mediated by NLRP3 inflammasome, and NF-κB has been demonstrated to regulate the NLRP3 inflammasome [76]. Consistently, our RNA-Seq results confirmed that Tat treatment significantly increased NLRP3 mRNA levels in control macrophages. Therefore, it is plausible that IKKβ/NF-κB signaling may have contributed to the observed caspase-1 activation in macrophages of Tg26^+/−^/ApoE^−/−^ mice [74]. In addition, human studies have demonstrated the increased expression of IKKβ/NF-κB-regulated proinflammatory genes such as IL-1β and IL-6 in monocytes and macrophages of HIV patients [34, 35]. These studies indicate that macrophage activation may contribute significantly to HIV-associated atherosclerosis. Future studies are required to investigate the detailed mechanisms through which IKKβ and other signaling pathways regulate macrophage functions to mediate HIV-induced atherosclerosis in animal models and humans.

In addition to HIV infection, the use of ARV drugs has also been associated with dyslipidemia and increased risk of CVD [77, 73, 78]. Despite the strong evidence linking certain ARV drugs with CVD risk, the underlying mechanisms responsible for the adverse effects of ARV drugs have not been well-characterized. Interestingly, we have previously demonstrated that several widely used ARV drugs, including efavirenz and amprenavir, can activate an important xenobiotic receptor, pregnane X receptor (PXR), to induce dysregulation of lipid homeostasis and hyperlipidemia [48, 79]. We also found that activation of PXR by other ligands can have pro-atherogenic effects in animal models [80–82, 45]. Therefore, it is plausible that activation of PXR by some ARV drugs may also lead to increased atherosclerosis and CVD risk in HIV patients. Consequently, it would be interesting to investigate how ARV drugs and HIV infection affect multiple signaling pathways, including but not limited to, IKKβ/NF-κB and PXR signaling, and promote the development of atherosclerosis in animal models and humans in the future.

In summary, we investigated the effects of HIV protein Tat on macrophage IKKβ signaling and atherosclerosis development in vivo employing atherosclerosis-prone IKKβ^ΔMye^LDLR^−/−^ and IKKβ^F/F^LDLR^−/−^ mice. Exposure to Tat protein significantly increased atherosclerosis in IKKβ^F/F^LDLR^−/−^ but not IKKβ^ΔMye^LDLR^−/−^ mice. Deficiency of myeloid IKKβ attenuated Tat-elicited macrophage inflammatory response and dysfunction, which likely contributed to reduced atherosclerosis in IKKβ^ΔMye^LDLR^−/−^ mice. These results revealed the atherogenic effects of HIV protein Tat in vivo and demonstrated a pivotal role of myeloid IKKβ in Tat-driven atherogenesis. Our findings in the present study will hopefully stimulate further investigations of the contribution of macrophage dysfunction to HIV-associated atherosclerosis and the underlying mechanisms through which IKKβ and other signaling pathways mediate HIV infection-elicited macrophage dysfunction and atherosclerosis.

## Supplementary Material

1668811_Sup
