# Supplementary material for: HIV Protein Tat Induces Macrophage Dysfunction and Atherosclerosis Development in Low-Density Lipoprotein Receptor-Deficient Mice
Source: Cardiovasc Drugs Ther. Author manuscript; Available in PMC 2023 Apr 1. (PMC8286272; doi:10.1007/s10557-021-07141-x)
Supplement: 1668811_Sup [file NIHMS1668811-supplement-1668811_Sup.pdf]

Supplementary Material for

HIV Protein Tat Induces Macrophage Dysfunction and Atherosclerosis Development in Low-Density Lipoprotein Receptor-Deficient Mice

Zhaojie Meng,[1] Rebecca Hernandez,[1] Jingwei Liu,[1] Taesik Gwag,[2] Weiwei Lu,[2] Tzung K Hsiai,[3] Marcus Kaul,[1] Tong Zhou,[4] and Changcheng Zhou[1]

[1]Division of Biomedical Sciences, School of Medicine, University of California, Riverside, CA
[2]Department of Pharmacology and Nutritional Sciences, College of Medicine, University of Kentucky, Lexington, KY
[3]Departments of Medicine and Bioengineering, David Geffen School of Medicine, Henry Samueli School of Engineering and Applied Science, University of California, Los Angeles, CA
[4]Department of Physiology and Cell Biology, Reno School of Medicine, University of Nevada, Reno, NV

This PDF file includes:

Supplemental Figure 1-4
Supplemental Table 1-2

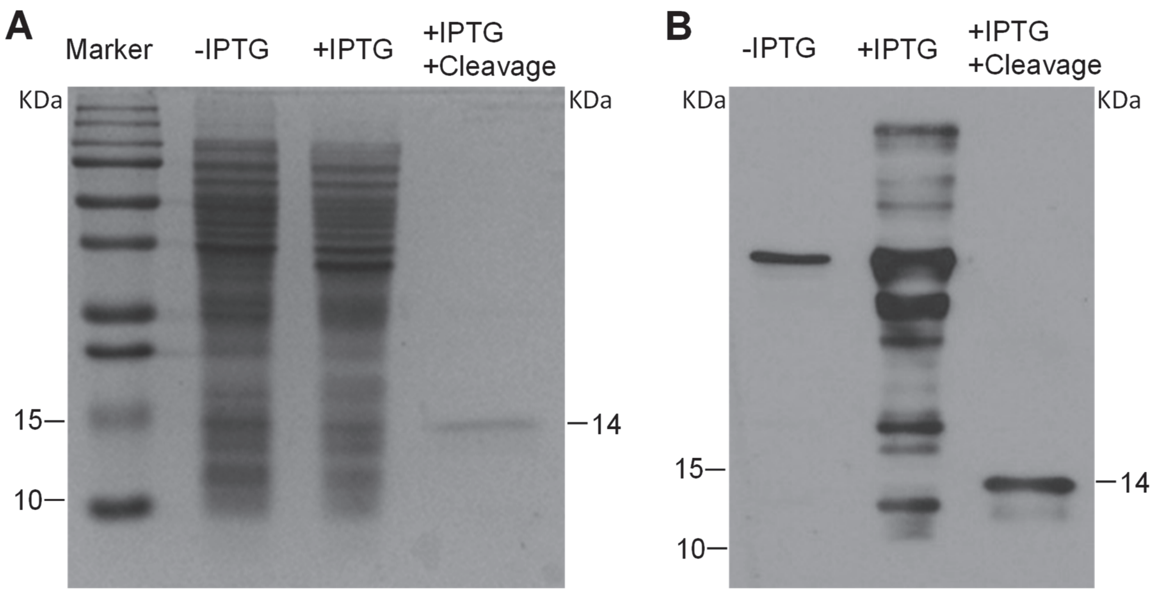

Supplemental Figure 1. Preparation and purification of recombinant Tat proteins. Recombinant HIV protein Tat_{1-72} was expressed as a glutathione S-transferase (GST)-fusion protein using an *Escherichia coli* vector with the induction of isopropyl β-D-1-thiogalactopyranoside (IPTG), purified by GST-affinity chromatography and cleaved from the fusion protein by thrombin protease. The process was verified by the Coomassie Blue staining (A) and Western blot analysis (B).

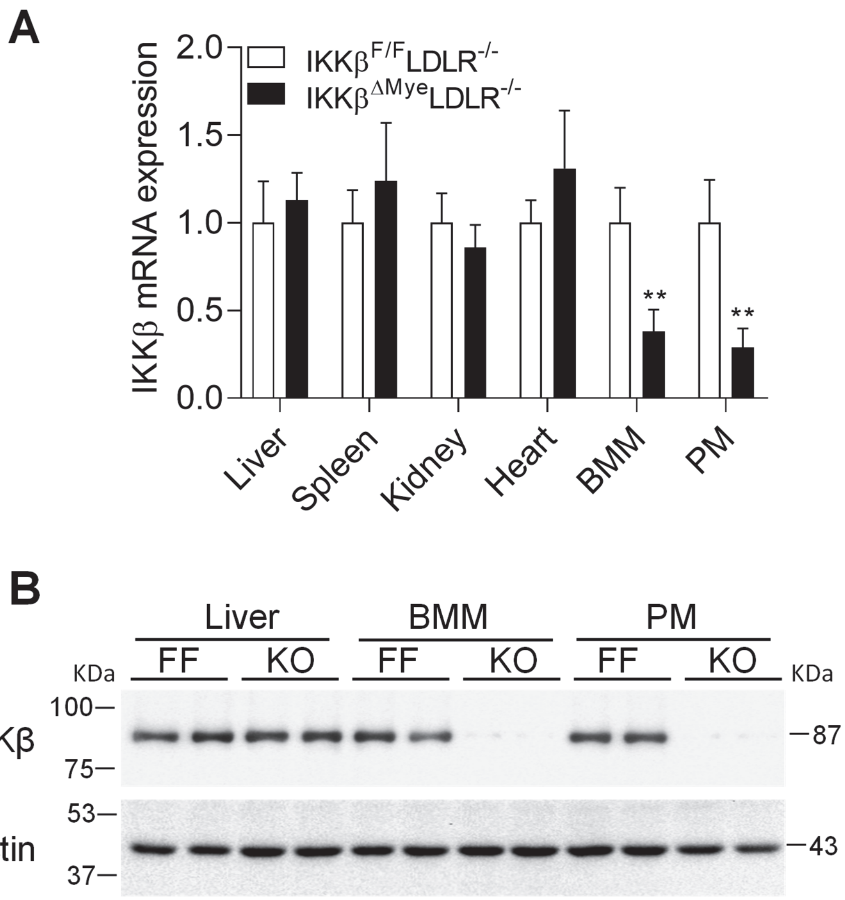

Supplementary Figure 2. Reduced IKKβ expression in macrophages of IKKβ$^{\Delta Mye}$LDLR$^{-/-}$ mice.

(A) mRNA levels of IKKβ in bone marrow-derived macrophages (BMM), peritoneal macrophages (PM), and the other major tissues of IKKβ$^{F/F}$LDLR$^{-/-}$ and IKKβ$^{\Delta Mye}$LDLR$^{-/-}$ mice (n=5, ***P*<0.01). (B) Immunoblotting for IKKβ proteins in Liver, PM and BMM of IKKβ$^{F/F}$LDLR$^{-/-}$ and IKKβ$^{\Delta Mye}$LDLR$^{-/-}$ mice.

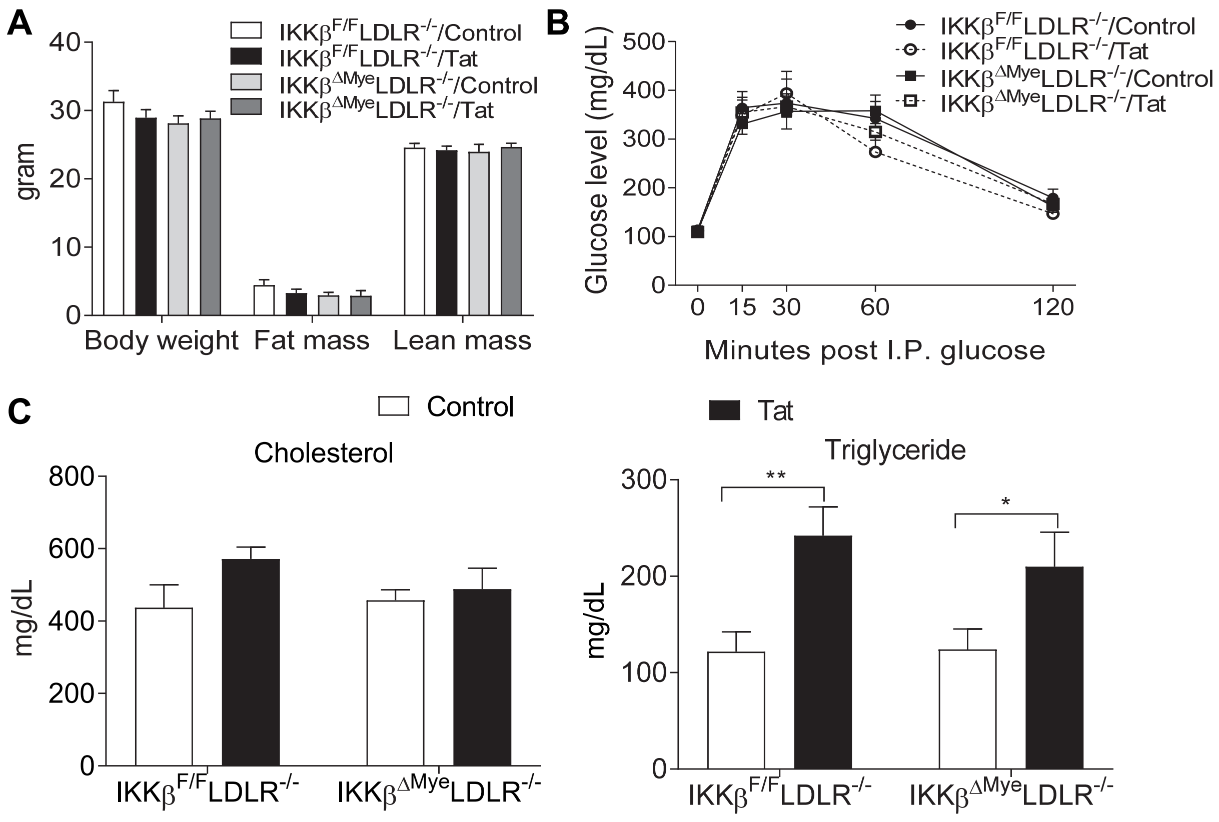

Supplemental Figure 3. Deficiency of myeloid IKKβ does not affect the impacts of HIV Tat on metabolic phenotypes and plasma lipid profiles.

Six-week-old male IKKβ$^{F/F}$LDLR$^{-/-}$ and IKKβ$^{\Delta Mye}$LDLR$^{-/-}$ littermates were fed a semi-synthetic AIN76a diet containing 0.02% cholesterol and treated with vehicle control or 1000 ng of Tat proteins by intravenous injection twice a week for 12 weeks. Body weight, fat and lean mass (A), glucose tolerance (B) and the plasma levels of cholesterol and triglyceride (C) were measured (n=6-8, *P<0.05 and **P<0.01).

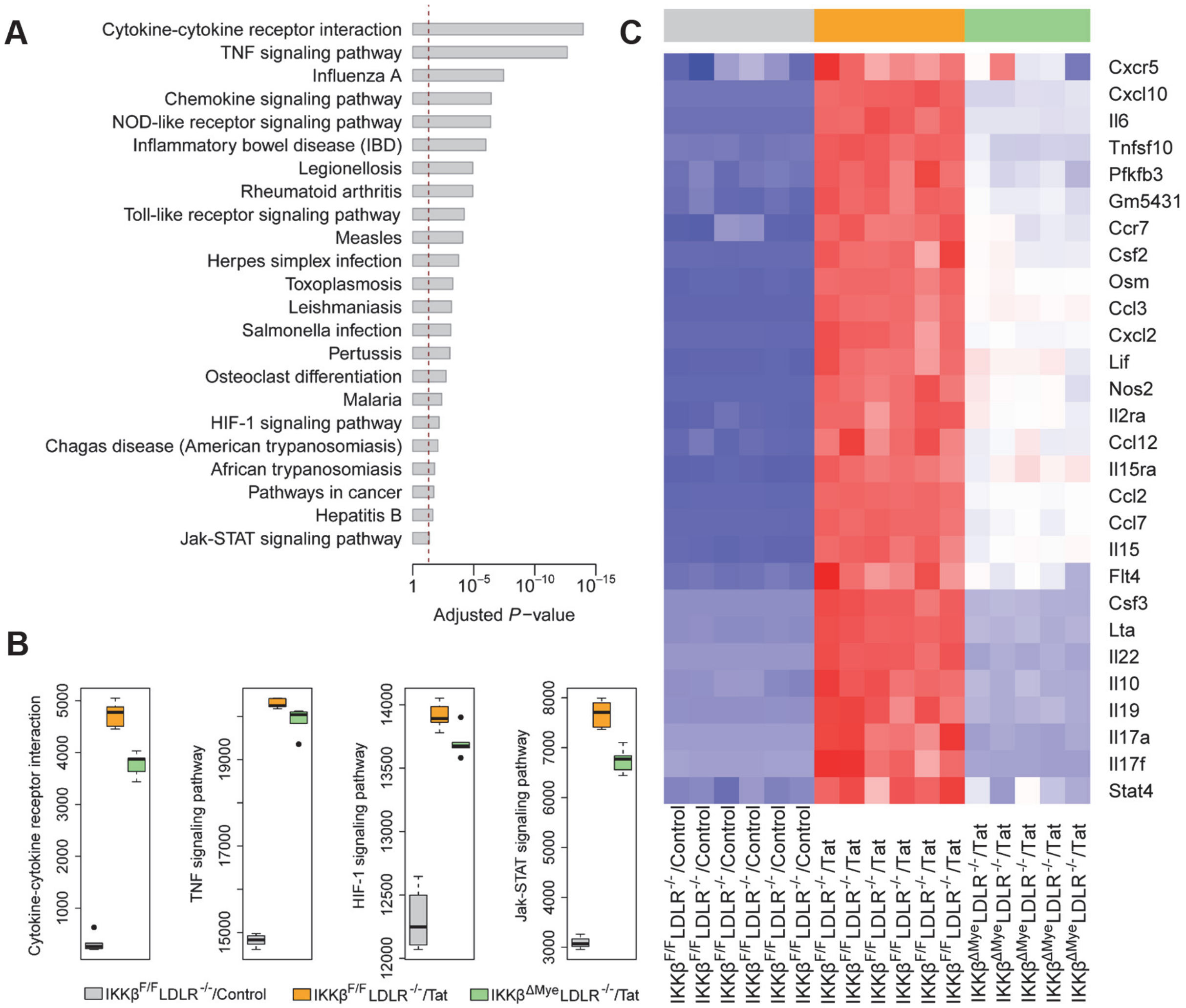

Supplemental Figure 4. Kyoto Encyclopedia of Genes and Genomes (KEGG) pathway analysis of RNA-Seq results from macrophages treated with control or HIV protein Tat. Peritoneal macrophages were isolated from IKKβ$^{F/F}$LDLR$^{-/-}$ and IKKβ$^{\Delta Mye}$LDLR$^{-/-}$ mice. Cells were treated with 100 nM Tat or vehicle control for 12 hours and total RNA was isolated for RNAseq analysis (n = 5-6). (A) The Kyoto Encyclopedia of Genes and Genomes (KEGG) pathways significantly associated with the DEGs in control macrophages after HIV Tat treatment. The *P*-values were computed by *Fisher*'s exact test. The vertical dash line indicates the significance level of α=0.05. The y-axis displays the KEGG pathways while the x-axis displays the *P*-values. (B) Geneset scores of the prioritized KEGG pathways. The geneset score was calculated using the *FAIME* algorithm. (C) Heatmap representation of DEGs involved in the pathways of "cytokine-cytokine receptor interaction", "TNF signaling pathway", "HIF-1 signaling pathway" and "Jak-STAT signaling pathway" shown in panel A and B. Each row shows one individual gene and each column a biological replicate of mouse. Red represents relatively increased gene expression while blue denotes downregulation.

Supplemental Table 1. Primer sequences for QPCR

Genes	Primer sequences	Genes	Primer sequences
CCR2	5'- GACGCTCAGATGCAAACCTT -3'	IL-6	5'-CCGAGGAAGACGTGGACACCTTC-3'
	5'- TCTTCTCCGCGCAGCTGCA -3'		5'-CCTCAGCCATGACCTGCCTTGTAG-3'
CCR5	5'-CAGTCGGAGACATGCT-3'	IL-12b	5'-AGGTCTCAGCCTTCTAAAGTTCCTC-3'
	5'-CTCGGGGTCCTGAGTT-3'		5'-TCTCTCGAATGAAATTTATCG-3'
ICAM-1	5'- GACGCTCAGATGCAAACCTT -3'	MCP-1	5'- GACGCTCAGATGCAAACCTT -3'
	5'- TCTTCTCCGCGCAGCTGCA -3'		5'- TCTTCTCCGCGCAGCTGCA -3'
IKKβ	5'- GACGCTCAGATGCAAACCTT -3'	TNFα	5'- GACGCTCAGATGCAAACCTT -3'
	5'- TCTTCTCCGCGCAGCTGCA -3'		5'- TCTTCTCCGCGCAGCTGCA -3'
IL-1β	5'- GACGCTCAGATGCAAACCTT -3'	VCAM-1	5'- GACGCTCAGATGCAAACCTT -3'
	5'- TCTTCTCCGCGCAGCTGCA -3'		5'- TCTTCTCCGCGCAGCTGCA -3'

Supplemental Table 2. Detailed RNAseq data of macrophage DEGs induced by HIV protein Tat

Gene	log2 (FC)	P-value	FDR	FC	Gene	log2 (FC)	P-value	FDR	FC
**Il17a**	11.52746183	1.05E-17	1.66E-16	2951.968955	**Schip1**	5.546570919	4.40E-21	1.31E-19	46.73951695
**Il22**	11.14292712	1.33E-18	2.48E-17	2261.285004	**Il23a**	5.545977951	1.51E-13	1.25E-12	46.7203103
**Nos2**	10.75825941	5.85E-34	4.23E-30	1732.043451	**Isg20**	5.54074417	9.54E-32	1.03E-28	46.55112633
**Edn1**	9.693469721	1.55E-30	9.06E-28	827.9901076	**Gm13889**	5.528973278	2.05E-11	1.29E-10	46.17286274
**Shisa3**	9.28785454	4.95E-22	1.78E-20	625.0615895	**Gbp5**	5.520946039	6.38E-32	7.28E-29	45.91666766
**Il19**	9.048145865	3.18E-20	8.08E-19	529.3748572	**Cish**	5.516376331	3.51E-27	4.58E-25	45.77145761
**Slamf1**	8.950240663	4.62E-29	1.36E-26	494.6419071	**Hap1**	5.507657827	5.43E-16	6.50E-15	45.49568538
**Il6**	8.930631517	4.65E-31	3.30E-28	487.9642068	**Hrc**	5.495043156	4.17E-14	3.75E-13	45.09961321
**Ptgs2**	8.792285954	2.07E-35	4.49E-31	443.3449876	**Rhcg**	5.489755865	4.88E-05	0.000146588	44.93463148
**Cxcl10**	8.602292898	1.52E-32	3.08E-29	388.640623	**Cmpk2**	5.467740225	2.95E-33	9.15E-30	44.25413092
**Lhx2**	8.59830964	5.27E-28	9.45E-26	387.5690724	**Apon**	5.459384066	7.96E-11	4.67E-10	43.99854973
**Csf3**	8.493565752	2.29E-32	3.55E-29	360.4276177	**Clic5**	5.429785999	1.22E-26	1.36E-24	43.10508004
**Csf2**	8.221330257	4.69E-26	4.40E-24	298.4468583	**Ccl17**	5.412563637	2.01E-12	1.44E-11	42.59356666
**Hdc**	8.04269652	2.68E-31	2.21E-28	263.6895374	**Mmp3**	5.388636582	6.19E-11	3.67E-10	41.89297927
**Ptx3**	7.995333678	6.10E-26	5.63E-24	255.1733189	**Plekha4**	5.357806988	3.41E-19	7.04E-18	41.00724703
**Il1b**	7.991602568	2.48E-34	2.68E-30	254.5142403	**Ccl12**	5.342231357	5.10E-19	1.02E-17	40.56690567
**Il27**	7.963964951	9.16E-33	2.21E-29	249.6849321	**Dll1**	5.321358646	1.23E-11	7.94E-11	39.98421463
**Il17f**	7.962694547	1.52E-17	2.36E-16	249.4651622	**Vgf**	5.312577702	4.48E-07	1.67E-06	39.74159038
**Hamp**	7.948007317	2.82E-15	3.04E-14	246.9383876	**Slc7a2**	5.310782616	5.68E-30	2.32E-27	39.69217229
**Il12a**	7.838566105	1.83E-30	1.03E-27	228.8988025	**Slc27a2**	5.303634846	3.41E-05	0.000104105	39.49600551
**Lipg**	7.772609898	3.86E-28	7.28E-26	218.6697577	**Gm15056**	5.300974091	1.77E-17	2.71E-16	39.42323035
**Tnfsf15**	7.44550119	7.70E-29	1.92E-26	174.308751	**Mycl**	5.294871296	3.73E-14	3.38E-13	39.25681699
**Has1**	7.341094108	1.32E-21	4.40E-20	162.1397606	**Krt16**	5.279423632	6.38E-07	2.34E-06	38.83871683
**Il1a**	6.944022248	1.23E-33	6.66E-30	123.128613	**Apobec4**	5.2787597	3.34E-05	0.000102194	38.82084727
**Gbp4**	6.942077466	6.72E-20	1.60E-18	122.9627449	**Cd40**	5.273054069	4.05E-33	1.10E-29	38.66762014
**Rnf225**	6.939248148	4.85E-23	2.15E-21	122.7218349	**Iigp1**	5.255963853	4.19E-30	1.93E-27	38.21226456
**Il1f6**	6.857306289	3.42E-08	1.45E-07	115.9457629	**Serpine1**	5.251364428	2.66E-29	8.74E-27	38.09063484
**Dnmt3c**	6.821665749	3.43E-13	2.72E-12	113.1165136	**Hcar2**	5.207282984	2.57E-31	2.21E-28	36.94437927
**Ccl5**	6.806814644	2.22E-31	2.08E-28	111.9580648	**Gm21748**	5.201111038	2.62E-21	8.24E-20	36.78666635
**Thbs4**	6.795445744	8.01E-15	8.04E-14	111.0792664	**Trem1**	5.198448048	4.18E-17	5.98E-16	36.71882651
**Alpk2**	6.788569542	3.11E-23	1.43E-21	110.5510979	**Pcp4**	5.19587958	0.0002391	0.000665956	36.6535132
**Ifng**	6.62654759	2.35E-23	1.12E-21	98.80742751	**Ifi208**	5.192750854	1.11E-29	4.13E-27	36.57411002
**Itgb8**	6.617633023	1.80E-25	1.47E-23	98.19876826	**Gbp10**	5.192448318	4.11E-12	2.81E-11	36.56644117
**Ch25h**	6.616720761	1.24E-28	2.74E-26	98.13669366	**Cd69**	5.142519743	4.16E-26	3.95E-24	35.32260265
**Iltifb**	6.597378766	6.28E-07	2.31E-06	96.82977027	**Hspa1a**	5.131323979	1.96E-28	4.09E-26	35.04954908
**Ccl7**	6.586822876	1.68E-29	5.89E-27	96.12387316	**Sprr2h**	5.119243263	3.86E-05	0.000117094	34.75727958
**Lif**	6.577964857	4.86E-24	2.80E-22	95.53548934	**Sele**	5.096033517	5.25E-07	1.94E-06	34.20258632
**Slc1a2**	6.560203731	8.71E-29	2.08E-26	94.36655501	**Lad1**	5.069185078	1.23E-23	6.40E-22	33.57196504
**Ifit1bl1**	6.55474832	5.19E-29	1.46E-26	94.01039084	**Scimp**	5.063544299	2.87E-28	5.67E-26	33.44095861
**Upp1**	6.493908926	5.04E-31	3.41E-28	90.12834078	**Xirp1**	5.058780532	0.0005883	0.001562349	33.33071895
**Epha4**	6.478228076	2.15E-06	7.47E-06	89.15402805	**Gm20708**	5.036826801	0.0015758	0.003993451	32.82735941
**Plagl1**	6.43655359	1.24E-20	3.43E-19	86.61551649	**Il10**	5.021200845	7.09E-17	9.74E-16	32.47372201
**Ifitm7**	6.428106259	8.30E-06	2.71E-05	86.10984335	**Ccl22**	5.018454286	1.65E-24	1.07E-22	32.41195834
**Inhba**	6.400537206	1.51E-29	5.37E-27	84.47995761	**Henmt1**	5.00059236	0.0005642	0.001502105	32.01314166
**Ccl3**	6.387822522	4.75E-32	5.72E-29	83.73869456	**Il17rd**	5.000462867	6.94E-14	6.02E-13	32.01026836
**Slco3a1**	6.383144001	5.59E-30	2.32E-27	83.46757789	**Calhm6**	4.978757232	5.31E-29	1.46E-26	31.53227229
**Nts**	6.381721719	3.80E-07	1.43E-06	83.38533182	**Gzmc**	4.972150627	3.82E-08	1.61E-07	31.38820509
**Cxcl2**	6.372881138	5.55E-29	1.47E-26	82.87592357	**Tarm1**	4.948082583	1.11E-27	1.71E-25	30.86890887
**AA467197**	6.270428933	7.81E-29	1.92E-26	77.19464861	**Olfr56**	4.932834611	4.03E-10	2.17E-09	30.54437068
**Areg**	6.2620888	9.76E-13	7.28E-12	76.74967837	**Cxcl9**	4.932123498	2.19E-22	8.45E-21	30.52931888
**Art3**	6.211330976	7.82E-06	2.56E-05	74.09637076	**Ntng2**	4.851266014	5.53E-24	3.17E-22	28.865334
**Cxcl1**	6.199019324	9.20E-29	2.12E-26	73.46673853	**Prom1**	4.840757797	0.0001289	0.000369848	28.65585022
**Etnk2**	6.120101355	3.95E-06	1.34E-05	69.55591761	**Vcam1**	4.836404104	1.20E-27	1.83E-25	28.56950439
**Tmem132e**	6.093160531	9.75E-11	5.65E-10	68.26908597	**Mmp13**	4.830501403	5.67E-30	2.32E-27	28.45285275
**Insrr**	6.050892752	1.36E-06	4.83E-06	66.29796818	**Neb**	4.824240483	6.06E-11	3.60E-10	28.32964234
**Pla1a**	6.034004202	3.30E-25	2.47E-23	65.5263925	**Hspa1b**	4.815548678	4.42E-27	5.61E-25	28.15947786
**Slc6a19**	5.953368088	0.0019252	0.00483385	61.96441725	**Gm8773**	4.80632435	2.99E-07	1.14E-06	27.98000567
**Serpina3g**	5.946269536	5.44E-29	1.46E-26	61.66028004	**Cxcl3**	4.801819867	1.22E-27	1.84E-25	27.89278079
**Urah**	5.94550855	4.12E-06	1.39E-05	61.62776431	**Fam3b**	4.781306001	4.40E-08	1.84E-07	27.49897617
**Fst**	5.926605513	1.18E-13	9.95E-13	60.82554821	**Apol9b**	4.775136559	1.81E-23	8.95E-22	27.38163253
**Il12b**	5.893999018	3.07E-32	3.92E-29	59.46624269	**Sprr2d**	4.771927344	2.13E-05	6.65E-05	27.32079091
**Ccnd2**	5.803222322	1.04E-29	3.96E-27	55.83981751	**Gbp3**	4.75571597	1.79E-33	7.74E-30	27.0155092
**Ccl4**	5.801151187	2.26E-32	3.55E-29	55.7597113	**Traf1**	4.738998814	2.07E-29	7.04E-27	26.70427504
**Ccl2**	5.792594324	3.15E-29	1.00E-26	55.42997009	**Trpm6**	4.713420661	0.0008649	0.00225918	26.23499593
**Il1rn**	5.784158124	2.61E-32	3.57E-29	55.10678762	**Pnp2**	4.687529065	5.80E-23	2.55E-21	25.76836444
**Tacr2**	5.77898075	3.04E-06	1.04E-05	54.9093813	**Ccl20**	4.682598719	0.0006597	0.001741766	25.68045251
**Rsad2**	5.75547747	1.56E-32	3.08E-29	54.02208698	**Coch**	4.681620565	1.29E-06	4.59E-06	25.66304695
**Gzmb**	5.747508474	1.27E-17	1.98E-16	53.7245085	**Il18**	4.658047575	9.90E-29	2.26E-26	25.24713145
**Serpina3f**	5.736076787	1.74E-27	2.50E-25	53.30048618	**Gstt4**	4.652898681	1.93E-06	6.74E-06	25.15718652
**Armcx4**	5.678212537	8.63E-17	1.17E-15	51.20499113	**A530032D15Rik**	4.646997073	5.84E-23	2.56E-21	25.05448665
**Heatr9**	5.65098009	1.73E-14	1.65E-13	50.24750615	**Dll4**	4.641913186	3.13E-11	1.92E-10	24.96635298
**Trim30c**	5.636684068	1.87E-30	1.03E-27	49.75205003	**Il15ra**	4.626944565	1.03E-25	8.93E-24	24.70865482
**Pax4**	5.613876542	9.56E-05	0.000278181	48.97170573	**Bmp10**	4.601608581	3.31E-06	1.13E-05	24.27852012
**Cd70**	5.611517937	7.08E-08	2.90E-07	48.89170924	**Calcr**	4.601180944	0.001339391	0.003428405	24.27132466
**Lta**	5.55497588	1.09E-28	2.46E-26	47.01261027	**Hbegf**	4.577747066	1.74E-25	1.43E-23	23.88026706
**Ifi205**	5.549544612	2.63E-32	3.57E-29	46.83595612	**Klrk1**	4.577661957	2.28E-23	1.09E-21	23.87885834
**Six1**	5.548136613	2.64E-12	1.86E-11	46.79026885	**Apol6**	4.575067433	0.001312814	0.003364347	23.8359535

Gene	log2 (FC)	P-value	FDR	FC	Gene	log2 (FC)	P-value	FDR	FC
**Ism1**	4.565543137	0.0001534	0.0004364	23.67911303	**Mbd3l2**	3.99094286	0.0037813	0.00913932	15.89986774
**Olfr2**	4.563824586	0.0008859	0.00231176	23.65092306	**Sphk1**	3.988401023	1.40E-25	1.18E-23	15.87187895
**Tnfsf10**	4.562691471	2.55E-25	1.97E-23	23.63235455	**Ifi206**	3.985066393	6.95E-30	2.74E-27	15.83523522
**Tmem171**	4.54949382	9.75E-19	1.85E-17	23.41715361	**Gdnf**	3.976480969	4.88E-06	1.64E-05	15.74128018
**Flrt3**	4.538274766	1.07E-27	1.67E-25	23.23575738	**Pln**	3.976115585	0.0040501	0.009744569	15.73729396
**Ms4a4c**	4.525597519	2.31E-31	2.08E-28	23.03247463	**Ppfia3**	3.968824816	1.45E-09	7.26E-09	15.65796497
**Sectm1a**	4.511218279	9.09E-07	3.28E-06	22.8040518	**Dnah5**	3.964572659	0.0012758	0.003272313	15.61188309
**Klri1**	4.5003052	1.44E-10	8.16E-10	22.63220429	**Phlda1**	3.957186071	1.00E-26	1.14E-24	15.53215464
**Igf2bp1**	4.492843479	6.15E-18	1.00E-16	22.51545112	**Nefh**	3.95277221	0.0027465	0.006755121	15.48470732
**Tnf**	4.478191425	2.91E-31	2.26E-28	22.28794079	**Krt23**	3.94771297	2.25E-06	7.80E-06	15.43050069
**Fosl1**	4.477626727	5.40E-18	8.90E-17	22.27921858	**Kcna2**	3.943778579	0.0002262	0.000631589	15.38847731
**Tnnt2**	4.467414129	0.0017726	0.00446816	22.12206458	**Kcna3**	3.940259436	1.04E-18	1.96E-17	15.35098619
**Mroh8**	4.467308829	2.16E-06	7.52E-06	22.12044998	**Cnn3**	3.937915208	1.05E-25	9.00E-24	15.3260627
**Dhh**	4.46652916	0.0009586	0.00249207	22.10849877	**Plaat3**	3.918779209	3.77E-24	2.23E-22	15.12411908
**Ereg**	4.466218212	1.58E-11	1.01E-10	22.10373417	**Pdgfrb**	3.91794248	1.23E-14	1.20E-13	15.11535
**Vnn3**	4.46519236	1.73E-26	1.84E-24	22.08802254	**Macc1**	3.913275333	6.77E-07	2.47E-06	15.06653055
**Ppp1r3g**	4.448710032	7.60E-06	2.49E-05	21.83710999	**Flt4**	3.905978747	1.84E-16	2.37E-15	14.99052231
**Slc4a11**	4.44201356	9.87E-08	3.97E-07	21.73598486	**Osm**	3.898224843	4.47E-29	1.35E-26	14.91017042
**Il2ra**	4.441665861	3.75E-21	1.14E-19	21.73074696	**Dok7**	3.89807097	1.45E-10	8.19E-10	14.90858024
**F3**	4.43665671	7.38E-25	5.22E-23	21.65542694	**Socs1**	3.89705463	1.24E-27	1.87E-25	14.89808124
**Eps8l2**	4.427832216	0.0017967	0.00452675	21.52337205	**Hba-a2**	3.896797306	0.0003400	0.000927916	14.89542421
**Axl**	4.423923002	2.28E-30	1.15E-27	21.46512996	**Srgn**	3.884648516	4.73E-30	2.14E-27	14.77051799
**Plet1**	4.421283247	1.16E-12	8.59E-12	21.42589029	**Gm4841**	3.870374885	7.80E-16	9.07E-15	14.62510305
**Enpp4**	4.419549218	1.82E-31	1.79E-28	21.40015317	**Batf2**	3.86054228	9.51E-27	1.09E-24	14.5257654
**Adamts4**	4.411213903	5.17E-13	4.01E-12	21.27686813	**Cd38**	3.857586894	1.69E-27	2.46E-25	14.49603958
**Vcan**	4.402753742	1.81E-27	2.58E-25	21.1524628	**Ifit1**	3.853250498	1.45E-29	5.25E-27	14.45253337
**Slc28a2**	4.399170452	8.59E-31	5.65E-28	21.09999061	**Ildr1**	3.852463581	1.23E-09	6.20E-09	14.44465239
**Cyp27b1**	4.387541382	0.0011837	0.00304887	20.93059447	**Fam131b**	3.847837509	0.0002037	0.000571711	14.39840909
**S1pr3**	4.379340072	1.59E-13	1.32E-12	20.81194756	**Scrt1**	3.847339445	0.0013200	0.003381233	14.39343916
**Arhgap8**	4.363889814	3.38E-05	0.00010314	20.59025531	**Zdhhc2**	3.834614869	2.69E-16	3.36E-15	14.26704718
**Tgtp1**	4.357823988	7.42E-28	1.22E-25	20.50386511	**Trp53i11**	3.821835258	2.94E-20	7.53E-19	14.14122562
**Gbp2**	4.348033906	1.80E-32	3.26E-29	20.36519758	**Pou3f1**	3.818278143	4.92E-17	6.97E-16	14.10640189
**Tgtp2**	4.345669002	1.17E-30	7.27E-28	20.33184175	**Vmn2r124**	3.812670106	0.0001346	0.000385411	14.05167399
**Spata31d1b**	4.332766848	3.57E-06	1.21E-05	20.15082287	**Efcab8**	3.812258366	0.0037844	0.009145855	14.04766427
**Eva1b**	4.332045068	1.26E-23	6.53E-22	20.14074394	**Dgat2**	3.811013874	6.33E-16	7.49E-15	14.03555175
**Myrfl**	4.329414658	0.0001370	0.00039216	20.10405556	**Mov10**	3.810561489	1.31E-30	7.88E-28	14.03115133
**Gm12185**	4.325618913	2.24E-20	5.86E-19	20.0512311	**Sprr2e**	3.806649483	5.27E-09	2.46E-08	13.99315605
**Socs3**	4.321606673	1.42E-31	1.46E-28	19.99554464	**4930467E23Rik**	3.793092961	0.003527885	0.008557459	13.8622829
**Ifi44**	4.317761143	1.59E-27	2.35E-25	19.94231712	**Olfr1033**	3.787598786	8.01E-22	2.74E-20	13.80959195
**Aplnr**	4.311594262	1.01E-05	3.27E-05	19.85725451	**Il15**	3.787088629	8.70E-27	1.01E-24	13.80470955
**Olfr1396**	4.297513968	9.30E-06	3.02E-05	19.66439598	**Il23r**	3.770980115	3.84E-07	1.44E-06	13.65142941
**Asap3**	4.2957974	1.22E-10	6.97E-10	19.64101252	**Sdc4**	3.765759908	2.03E-28	4.19E-26	13.60212272
**Isg15**	4.294929648	7.50E-29	1.89E-26	19.62920241	**Ikzf4**	3.758322274	2.94E-08	1.26E-07	13.53217912
**Mtmr7**	4.284534573	4.67E-20	1.15E-18	19.4882761	**Xkr8**	3.757302917	3.93E-28	7.34E-26	13.52262114
**Syt7**	4.279859607	3.69E-13	2.91E-12	19.42522773	**Xcl1**	3.742673243	3.56E-12	2.46E-11	13.38618769
**Angpt1**	4.278653649	9.47E-08	3.81E-07	19.40899684	**Pamr1**	3.735122313	4.05E-08	1.70E-07	13.31630868
**Csrnp1**	4.269431321	2.10E-28	4.30E-26	19.28532189	**Mybpc2**	3.734066941	3.47E-05	0.000105931	13.30657098
**Peg10**	4.253280771	1.33E-06	4.71E-06	19.07063228	**Sytl3**	3.733297571	2.32E-09	1.13E-08	13.29947666
**Pcsk1**	4.249881692	0.0024902	0.00615953	19.02575357	**Bco2**	3.729435265	3.19E-16	3.92E-15	13.26391963
**Cdh15**	4.245361674	0.0013809	0.00353014	18.96623845	**Timp1**	3.721628592	1.99E-25	1.61E-23	13.1923401
**Apol9a**	4.243133371	2.62E-18	4.59E-17	18.93696691	**Nlrp3**	3.707857403	2.94E-27	3.94E-25	13.06701224
**Trim69**	4.232883209	0.0011439	0.00295058	18.80289903	**Casp7**	3.698213792	1.89E-30	1.03E-27	12.97995784
**Epb41l4b**	4.232331537	1.08E-06	3.87E-06	18.79571038	**Ptges**	3.687934467	2.14E-30	1.10E-27	12.88780323
**Tpbg**	4.228355569	6.60E-16	7.80E-15	18.743982	**Tnfaip3**	3.687104161	9.91E-31	6.32E-28	12.88038812
**Styk1**	4.226615946	1.45E-11	9.31E-11	18.72139365	**Ccdc184**	3.663373965	0.0001927	0.000542521	12.67025772
**Rnase10**	4.224747878	5.63E-06	1.88E-05	18.69716822	**Marcksl1**	3.662148991	2.90E-30	1.40E-27	12.65950413
**Adora2b**	4.220275263	7.37E-30	2.85E-27	18.63929338	**Lpar1**	3.661357678	3.72E-21	1.13E-19	12.65256235
**Slfn4**	4.2200174	6.88E-27	8.24E-25	18.63596215	**Tnfaip6**	3.657639321	1.58E-09	7.85E-09	12.61999402
**Cst7**	4.207767835	4.71E-19	9.48E-18	18.47839868	**Il33**	3.651452445	1.00E-12	7.46E-12	12.56599012
**Draxin**	4.198963132	2.19E-09	1.07E-08	18.36596928	**Oas1d**	3.637084072	0.0035094	0.008515563	12.44146157
**Ifit2**	4.188054466	5.52E-30	2.32E-27	18.22762217	**Slc6a4**	3.636438178	2.52E-09	1.22E-08	12.43589278
**Elfn1**	4.180952491	1.31E-05	4.20E-05	18.1381133	**Lmcd1**	3.629605218	5.78E-05	0.000172012	12.37713258
**Pde11a**	4.165335147	3.44E-08	1.46E-07	17.94282499	**Atp10a**	3.625005457	1.82E-28	3.84E-26	12.33773327
**Serpinb2**	4.150531318	8.20E-28	1.31E-25	17.7596509	**Col27a1**	3.622597748	6.51E-11	3.85E-10	12.31716004
**Vmn1r84**	4.136406267	0.0022332	0.00556122	17.58661923	**Phf11b**	3.621653898	2.30E-29	7.66E-27	12.30910446
**Oasl1**	4.135308165	2.75E-31	2.21E-28	17.57323833	**Ccrl2**	3.616768058	4.55E-31	3.30E-28	12.26748888
**Garem2**	4.131255548	4.03E-07	1.51E-06	17.52394331	**Spats2l**	3.612418634	6.19E-05	0.000183586	12.23056065
**Htra4**	4.120985512	1.53E-18	2.82E-17	17.39963951	**Ifi213**	3.611381713	1.37E-27	2.04E-25	12.22177323
**Txndc2**	4.119862895	0.002845916	0.006986866	17.38610546	**Kdm6b**	3.609865123	3.64E-26	3.54E-24	12.20893221
**Chic1**	4.117099742	2.97E-14	2.73E-13	17.3528382	**Ifit3b**	3.596466891	1.28E-28	2.80E-26	12.09607338
**Zfp811**	4.113487261	5.33E-14	4.71E-13	17.30944138	**Tnfrsf9**	3.588700859	8.59E-14	7.36E-13	12.03113511
**Gm43302**	4.102299703	0.001454438	0.00370365	17.17573227	**F830016B08Rik**	3.583848099	2.79E-09	1.35E-08	11.99073424
**Fam160a1**	4.073306001	0.001238518	0.003182977	16.83399865	**Rab11fip1**	3.578069175	2.06E-27	2.90E-25	11.94279969
**Ms4a4d**	4.073054721	9.92E-08	3.99E-07	16.83106685	**Timeless**	3.549212577	8.32E-23	3.54E-21	11.70629453
**Acod1**	4.067793058	2.80E-33	9.15E-30	16.76979395	**Alox12**	3.547660251	1.90E-08	8.30E-08	11.69370544
**Tmem200b**	4.060094425	1.57E-06	5.54E-06	16.68054388	**Mlkl**	3.545953255	2.87E-29	9.30E-27	11.67987764
**Oprd1**	4.058442317	1.73E-05	5.44E-05	16.66145303	**1700012B09Rik**	3.543313429	4.21E-06	1.42E-05	11.65852548
**Nt5c1a**	4.055281887	9.60E-11	5.57E-10	16.62499368	**Ifit3**	3.543115182	1.37E-29	5.02E-27	11.65692354
**Src**	4.043267508	1.16E-28	2.60E-26	16.48712005	**Chrna5**	3.537456286	2.64E-07	1.01E-06	11.61128943
**Has2**	4.038273274	7.29E-08	2.98E-07	16.43014461	**Sema7a**	3.517693542	2.25E-16	2.84E-15	11.45331675
**Mfsd2a**	4.003874891	4.79E-05	0.00014380	16.04303168	**Vmn2r79**	3.514557351	7.96E-06	2.60E-05	11.42844609

Gene	log2 (FC)	P-value	FDR	FC	Gene	log2 (FC)	P-value	FDR	FC
**Ifnb1**	3.513929174	0.0001311	0.00037595	11.42347102	**Pmepa1**	3.139852294	3.27E-20	8.27E-19	8.814338452
**Thbs1**	3.510934788	2.08E-29	7.04E-27	11.39978562	**Nlgn2**	3.126199907	1.85E-15	2.05E-14	8.73132079
**Nfkb2**	3.498799573	4.71E-31	3.30E-28	11.30429859	**Tnfsf4**	3.124618835	1.32E-08	5.87E-08	8.72175724
**Ier3**	3.487889653	4.92E-28	8.90E-26	11.21913586	**Lacc1**	3.111371697	7.22E-28	1.19E-25	8.64203873
**Serpina11**	3.475406875	0.0016490	0.00417058	11.12248213	**Phf11c**	3.111018522	6.97E-20	1.66E-18	8.639923403
**Gm17535**	3.472329037	0.0021710	0.00541703	11.09877878	**Gbp6**	3.109061269	2.03E-27	2.88E-25	8.628209871
**Slc6a20a**	3.462936042	0.0001818	0.00051297	11.02675237	**Gng4**	3.108444585	1.26E-08	5.63E-08	8.62452251
**Dusp14**	3.443150022	1.67E-07	6.52E-07	10.8765569	**Cfb**	3.107992521	4.89E-30	2.16E-27	8.621820466
**Micall2**	3.440655911	1.22E-22	4.96E-21	10.8577699	**1600014C10Rik**	3.107771632	9.32E-27	1.08E-24	8.62050049
**Zfp819**	3.440228549	9.13E-05	0.000266532	10.85455404	**Cstdc4**	3.10219676	0.003582199	0.00868532	8.587253372
**Sod3**	3.434314328	2.08E-12	1.49E-11	10.81014769	**Kctd4**	3.087824496	2.52E-09	1.23E-08	8.502131049
**Plek2**	3.432903215	2.49E-05	7.71E-05	10.79957935	**Zyx**	3.087489695	5.38E-29	1.46E-26	8.500158215
**Usp18**	3.431453775	7.86E-29	1.92E-26	10.78873473	**Gm10719**	3.086928871	0.002009753	0.005035529	8.496854564
**Phf11a**	3.427418001	2.27E-27	3.13E-25	10.75859666	**Ctnnd2**	3.086897903	8.03E-07	2.91E-06	8.496672177
**Lcn2**	3.427064433	5.32E-29	1.46E-26	10.75596032	**Slc15a3**	3.077596553	3.48E-30	1.64E-27	8.442068604
**P2ry14**	3.417363163	2.32E-24	1.44E-22	10.68387549	**Trim30b**	3.068345128	2.66E-18	4.64E-17	8.388106204
**Gm16181**	3.416627443	9.33E-14	7.97E-13	10.67842851	**Pgf**	3.067100221	0.0002027	0.000569055	8.380871197
**Gipr**	3.414698789	0.002976776	0.007289975	10.66416268	**Nfkbiz**	3.063732438	1.16E-26	1.30E-24	8.361329965
**Gbp7**	3.408966999	9.09E-26	8.02E-24	10.6218783	**Eng**	3.059703756	2.94E-27	3.94E-25	8.33801378
**Procr**	3.407716521	1.88E-26	1.98E-24	10.6126756	**Car2**	3.057909734	5.38E-11	3.21E-10	8.327651722
**Tlr3**	3.397952687	1.28E-24	8.55E-23	10.54109392	**Car13**	3.057904995	5.29E-27	6.60E-25	8.32762437
**Tmem67**	3.396131439	3.12E-26	3.11E-24	10.52779531	**Nlrc5**	3.05470119	8.66E-23	3.67E-21	8.309151663
**Tjp1**	3.394630847	1.33E-18	2.49E-17	10.51685071	**Furin**	3.053916986	3.00E-28	5.82E-26	8.304636296
**Nt5c3**	3.392045799	4.64E-29	1.36E-26	10.49802329	**Rcl1**	3.050158363	9.98E-26	8.73E-24	8.283028559
**Ms4a12**	3.386700249	1.28E-05	4.10E-05	10.45919744	**Hif3a**	3.049625456	0.001822062	0.004588592	8.279969517
**Adora2a**	3.386323561	3.16E-27	4.21E-25	10.4564669	**Ifit1bl2**	3.047009699	3.89E-14	3.51E-13	8.264970665
**Mrgpra2b**	3.381503727	0.0018866	0.00474336	10.42159165	**Pdpn**	3.042879268	5.50E-27	6.78E-25	8.241341925
**Synpo2**	3.377118732	1.02E-12	7.61E-12	10.38996386	**Aldh1b1**	3.025167928	4.03E-26	3.85E-24	8.140785062
**Adm**	3.374466605	7.23E-10	3.76E-09	10.3708814	**Olfr656**	3.025167413	0.0020482	0.005127822	8.140782155
**Gm10800**	3.370695177	0.0015190	0.00385953	10.34380571	**Htr1b**	3.018921031	0.0005102	0.001364472	8.105611509
**Dlgap3**	3.369243437	0.0015309	0.00388699	10.33340229	**Cd274**	3.013438586	6.51E-28	1.11E-25	8.074867497
**Cd86**	3.360839521	1.19E-27	1.83E-25	10.27338364	**Pla2g4c**	3.012354516	3.51E-07	1.33E-06	8.068802159
**Gadd45b**	3.355618726	5.78E-28	1.02E-25	10.23627372	**Il4i1**	3.012183658	7.01E-19	1.36E-17	8.067846628
**Rab39b**	3.353313508	0.0014097	0.00359864	10.2199307	**Ifi204**	3.01214723	3.67E-29	1.12E-26	8.067642919
**Helz2**	3.353251033	2.40E-24	1.47E-22	10.21948814	**Hivep3**	3.009300718	2.03E-19	4.37E-18	8.051740732
**Zc3h12a**	3.346349832	5.65E-29	1.47E-26	10.17071947	**Hilpda**	3.004502067	9.87E-25	6.76E-23	8.025003752
**Gm4951**	3.343937654	1.50E-22	5.94E-21	10.15372828	**Tcp10b**	2.99959076	2.86E-10	1.57E-09	7.997731016
**Ifih1**	3.337714778	5.11E-29	1.46E-26	10.11002583	**Oas1g**	2.999328057	6.13E-27	7.39E-25	7.996274822
**Ifi209**	3.332984715	3.30E-29	1.04E-26	10.07693308	**Kazn**	2.997335152	1.51E-13	1.26E-12	7.985236585
**Phf11d**	3.324753848	5.12E-29	1.46E-26	10.01960582	**Gipc3**	2.995787928	1.89E-08	8.25E-08	7.976677379
**Txlnb**	3.319507221	6.46E-15	6.57E-14	9.983233852	**Zhx2**	2.988271752	1.41E-23	7.17E-22	7.935228415
**Misp**	3.318979824	4.08E-12	2.79E-11	9.979585011	**Mmp25**	2.988264936	5.49E-14	4.83E-13	7.935190926
**Nr4a3**	3.314004337	3.21E-18	5.57E-17	9.945227247	**Kalrn**	2.985044381	2.83E-09	1.37E-08	7.91749681
**Car4**	3.293137871	4.42E-05	0.00013330	9.802419377	**Cemip2**	2.983036723	2.56E-24	1.56E-22	7.906486463
**Ifi211**	3.288922236	9.03E-29	2.11E-26	9.773817972	**Hmgn3**	2.975492332	2.83E-14	2.61E-13	7.865248414
**Igtp**	3.279663337	5.86E-30	2.35E-27	9.71129261	**Sell**	2.975412322	1.01E-18	1.92E-17	7.864812231
**Ifi47**	3.279165228	2.41E-30	1.19E-27	9.707940241	**Glipr2**	2.970429962	6.26E-26	5.75E-24	7.837697877
**Bmp8b**	3.270693967	0.0007970	0.00208901	9.651103892	**Met**	2.965050169	1.83E-25	1.49E-23	7.808525622
**Tagap**	3.265549446	1.38E-26	1.50E-24	9.61675021	**Sema4c**	2.963905429	2.36E-16	2.98E-15	7.80233222
**Pde4b**	3.2636804	8.99E-29	2.11E-26	9.604299547	**Pnp**	2.961965065	4.22E-27	5.38E-25	7.791845464
**Gm2237**	3.262881283	0.0005494	0.00146427	9.598981141	**Pdcd1**	2.961665359	2.01E-07	7.81E-07	7.790226955
**Spry1**	3.257687043	1.05E-15	1.20E-14	9.564483371	**Sp140**	2.957655209	6.15E-29	1.59E-26	7.768603118
**Epha1**	3.256731646	0.0003201	0.00087602	9.558151572	**Rab44**	2.951474064	8.84E-08	3.58E-07	7.735390176
**Pla2g4f**	3.255794418	0.0001586	0.00045035	9.551944264	**Herc6**	2.949551498	1.60E-28	3.41E-26	7.725088696
**Dusp16**	3.24028633	5.70E-28	1.01E-25	9.449816597	**Malt1**	2.942507402	3.95E-26	3.80E-24	7.68746215
**Clrn3**	3.240159851	7.24E-06	2.38E-05	9.448988183	**Penk**	2.941534798	1.56E-11	9.97E-11	7.682281336
**Cdh5**	3.22558324	3.17E-13	2.53E-12	9.353998776	**Uba7**	2.939543648	4.85E-24	2.80E-22	7.671685873
**Elovl2**	3.201092453	7.22E-06	2.38E-05	9.196548115	**Pcdh17**	2.924229493	0.0003735	0.001014549	7.590681893
**Gm10717**	3.195983521	0.0020662	0.00516983	9.164038509	**Slc2a6**	2.91415933	9.24E-28	1.46E-25	7.537882623
**Mndal**	3.193858913	3.48E-28	6.62E-26	9.150552875	**Gm49342**	2.912020699	6.81E-24	3.80E-22	7.526716851
**Fabp3**	3.193489133	5.37E-27	6.66E-25	9.148207774	**Fhod3**	2.910730062	0.0002906	0.000799866	7.519986446
**Crtam**	3.192651531	9.17E-07	3.31E-06	9.142898038	**Irf7**	2.91035726	7.35E-29	1.88E-26	7.518043487
**Mab21l3**	3.190827159	2.68E-06	9.26E-06	9.131343615	**Mxd1**	2.908601692	1.97E-26	2.06E-24	7.508900587
**Mid1**	3.189950725	2.42E-24	1.48E-22	9.125798031	**Sh3bp5**	2.908474646	1.36E-26	1.49E-24	7.508239371
**Casp4**	3.189753134	3.47E-29	1.07E-26	9.124548252	**St3gal5**	2.907587814	6.10E-28	1.07E-25	7.503625441
**Gm21738**	3.189387524	0.0023974	0.00594565	9.122236186	**Gbp9**	2.904950116	3.12E-25	2.36E-23	7.489918996
**Bcl6b**	3.188934216	4.68E-06	1.57E-05	9.119370344	**Jak2**	2.89840843	3.21E-28	6.17E-26	7.456033957
**Vmn1r60**	3.181718999	0.0027321	0.00672347	9.073876342	**Ak4**	2.896960408	1.96E-18	3.50E-17	7.448554146
**Slamf9**	3.180942741	7.73E-25	5.43E-23	9.068995359	**Irf1**	2.892075728	2.28E-27	3.13E-25	7.423377459
**Daxx**	3.180168019	8.35E-29	2.01E-26	9.064126646	**Akap12**	2.888583641	8.73E-13	6.55E-12	7.405430681
**Ermap**	3.179244474	1.62E-05	5.13E-05	9.058326076	**Trim43b**	2.87820743	0.0039048	0.009419091	7.352360113
**Gja1**	3.179014657	6.31E-21	1.83E-19	9.056883228	**Lrrc8c**	2.870725691	2.11E-27	2.95E-25	7.314329865
**BC147527**	3.166510941	5.74E-18	9.44E-17	8.978727164	**Ccr7**	2.862355202	2.19E-15	2.39E-14	7.272015135
**Serpinb6b**	3.165271568	6.53E-14	5.69E-13	8.971017141	**Csta2**	2.854608816	1.50E-10	8.48E-10	7.233073518
**Nmi**	3.16183892	2.00E-26	2.07E-24	8.949697502	**Pim1**	2.85439861	5.70E-27	6.91E-25	7.232019711
**Magee2**	3.15828375	0.0012956	0.00332193	8.927670308	**Carlr**	2.852254754	4.85E-05	0.000145656	7.221280854
**Phactr1**	3.154984024	1.54E-10	8.70E-10	8.907274306	**Il1f9**	2.848598804	8.24E-20	1.93E-18	7.203004492
**Bend7**	3.146982988	0.0005758	0.00153049	8.858012223	**Xaf1**	2.846137954	1.12E-26	1.26E-24	7.190728575
**Jaml**	3.146396571	2.05E-10	1.14E-09	8.854412409	**Aqp9**	2.839492866	4.18E-26	3.95E-24	7.157684067
**Sprr2b**	3.144271015	0.0010311	0.00267324	8.841376604	**Nrg1**	2.83930517	2.79E-14	2.58E-13	7.156752905
**Arg2**	3.142755356	1.77E-20	4.79E-19	8.832092954	**Pdgfrl**	2.838644305	0.0001753	0.000495381	7.153475317

Gene	log2 (FC)	P-value	FDR	FC	Gene	log2 (FC)	P-value	FDR	FC
Zbp1	2.837211373	2.17E-28	4.37E-26	7.146373774	Slc7a11	2.652664991	3.19E-25	2.40E-23	6.288277964
Abca13	2.836521308	0.0001424	0.00040685	7.142956362	Wfdc21	2.651924668	2.82E-06	9.68E-06	6.285051943
Irgm1	2.83637124	7.49E-28	1.22E-25	7.142213398	Klrg2	2.649700943	3.49E-06	1.19E-05	6.275371821
Milr1	2.817549177	4.08E-27	5.24E-25	7.049637999	Unc93a	2.649516536	0.0014081	0.003595342	6.27456975
Gnb4	2.815469441	7.89E-12	5.21E-11	7.039482822	Sp110	2.645599475	8.23E-28	1.31E-25	6.257556772
Oas3	2.809808376	2.12E-27	2.95E-25	7.011914362	Tbx21	2.641778273	2.25E-10	1.24E-09	6.241004592
Rasgrp1	2.807399261	2.15E-24	1.35E-22	7.000215136	Tnfsf9	2.640737419	2.64E-24	1.60E-22	6.236503549
Clcf1	2.802719158	7.61E-13	5.76E-12	6.977543234	Stk40	2.63843283	3.38E-27	4.45E-25	6.226549192
Slfn9	2.802503855	7.99E-23	3.44E-21	6.976502002	Jag1	2.636854986	1.19E-23	6.18E-22	6.219743071
Ell2	2.801964297	4.91E-26	4.59E-24	6.973893328	Gem	2.631607536	2.15E-16	2.73E-15	6.197161374
Casp12	2.799740041	0.0034889	0.00846767	6.963149705	Stk39	2.631096916	1.71E-14	1.63E-13	6.19496837
Pde6b	2.799089074	0.0001452	0.00041460	6.96000853	Ddx58	2.630261805	1.97E-26	2.06E-24	6.19138342
Rnf180	2.794748158	8.22E-21	2.33E-19	6.939098076	Clmp	2.62960232	3.62E-25	2.69E-23	6.188553861
Trim56	2.783061482	9.28E-26	8.15E-24	6.883114372	Apobec3	2.626166221	2.26E-25	1.79E-23	6.173831982
Gch1	2.781437516	8.09E-27	9.59E-25	6.875370767	Crim1	2.625988359	2.07E-20	5.48E-19	6.173070888
Mefv	2.779853961	1.70E-26	1.82E-24	6.867828252	Gm29094	2.621607576	2.20E-16	2.79E-15	6.154354624
Hsh2d	2.779647744	5.68E-10	3.00E-09	6.866846642	Vmn2r114	2.618096925	6.23E-06	2.06E-05	6.139396834
Dcbld1	2.77891343	0.0003211	0.00087865	6.863352389	Rcn1	2.613553727	2.84E-20	7.30E-19	6.12009364
Ugt3a1	2.777702601	2.36E-05	7.34E-05	6.857594515	Tnfrsf8	2.61230574	8.72E-15	8.71E-14	6.114801811
Slpi	2.777271875	8.71E-24	4.72E-22	6.855547438	Npnt	2.610090744	0.0003188	0.000873027	6.105420849
Foxf1	2.776043411	0.0001029	0.00029843	6.849712384	Zfp36	2.610004923	1.69E-27	2.46E-25	6.105057669
Gm12216	2.775351398	2.38E-09	1.16E-08	6.846427588	Fpr1	2.6075517	1.57E-25	1.31E-23	6.094685176
Camk2n1	2.770575395	2.20E-10	1.22E-09	6.823800151	AC167036.2	2.605750212	4.42E-05	0.000133209	6.087079514
Trim72	2.769490639	0.0011608	0.00299166	6.818671295	Parp12	2.603441346	6.98E-28	1.16E-25	6.077345641
Krt222	2.769355632	1.66E-06	5.83E-06	6.818033237	Olfr709-ps1	2.591361627	2.57E-06	8.88E-06	6.026672329
Usb1	2.767058016	1.53E-28	3.31E-26	6.80718357	Irgm2	2.590493306	8.62E-27	1.01E-24	6.023046118
Stx1b	2.766043933	0.0023150	0.00575449	6.802400423	Fap	2.588572467	4.62E-06	1.56E-05	6.015032226
Sntb1	2.764805316	1.23E-18	2.29E-17	6.796562771	H2-Q6	2.582819118	2.73E-25	2.08E-23	5.991092542
Tnfsf8	2.762532998	1.87E-11	1.18E-10	6.785866265	Zeb1	2.581130446	3.72E-14	3.37E-13	5.98408408
Frzb	2.760990553	0.0026443	0.00651781	6.778615092	Tnip1	2.580211758	2.88E-28	5.67E-26	5.980274712
Bcl2a1d	2.754451909	1.54E-26	1.67E-24	6.747962281	Slc25a22	2.577814688	3.97E-23	1.80E-21	5.970346601
Exoc3l4	2.752819481	5.86E-10	3.09E-09	6.740331192	Slamf7	2.576790626	1.08E-26	1.21E-24	5.966110199
Nfkbib	2.750149871	1.04E-26	1.18E-24	6.727870195	Mt2	2.574138135	6.88E-22	2.39E-20	5.955151186
Clic4	2.744386588	4.44E-28	8.17E-26	6.701047288	Fmnl2	2.571114276	7.54E-22	2.60E-20	5.942682383
Palld	2.742892193	3.10E-20	7.88E-19	6.694109698	Tmtc2	2.568668123	1.89E-12	1.35E-11	5.932614843
Epop	2.739666545	4.25E-12	2.90E-11	6.679159398	H2-M2	2.565921602	5.73E-25	4.16E-23	5.92133141
Gm5431	2.739635804	7.41E-25	5.22E-23	6.679017081	Fbn1	2.563756073	9.05E-17	1.22E-15	5.912449978
Sass6	2.738520758	1.36E-17	2.13E-16	6.673856923	Acp5	2.560313788	3.80E-27	4.90E-25	5.898359629
1110032F04Rik	2.7384311	1.13E-09	5.72E-09	6.673442182	Etnk1	2.55586924	1.20E-25	1.01E-23	5.880216361
Rnd1	2.736765106	6.77E-19	1.32E-17	6.665740278	Dcbld2	2.553962216	1.13E-24	7.61E-23	5.872448744
Rgs1	2.736685404	2.68E-17	3.99E-16	6.665372041	Gm6377	2.553509893	3.61E-21	1.10E-19	5.870607865
Tuba8	2.73296573	3.60E-08	1.52E-07	6.648208967	F10	2.550253679	4.26E-28	7.89E-26	5.857372635
Igf2bp3	2.732660715	0.000209484	0.000587307	6.646803551	Cks2	2.547670353	3.66E-14	3.32E-13	5.84689366
Ifi203	2.7324567	3.98E-26	3.82E-24	6.645863676	Tpst1	2.547162866	7.31E-24	4.02E-22	5.844837303
Fam102b	2.732435393	1.69E-24	1.09E-22	6.645765524	Igsf8	2.544479217	2.08E-26	2.15E-24	5.833975052
Ddx4	2.72872225	2.86E-09	1.38E-08	6.62868295	Tap1	2.543066573	2.14E-28	4.34E-26	5.828265395
Cflar	2.725843424	1.75E-25	1.44E-23	6.61546893	AC133103.1	2.542356886	0.0008087	0.00211835	5.825399075
Asb2	2.723845563	3.04E-11	1.87E-10	6.606314093	Muc1	2.541964557	0.0001374	0.000393091	5.82381512
1700019D03Rik	2.722046611	1.56E-05	4.96E-05	6.59808156	Abtb2	2.540011033	2.28E-24	1.42E-22	5.815934547
Fscn1	2.721992701	2.32E-17	3.49E-16	6.59783501	Ifi35	2.539007414	9.14E-25	6.31E-23	5.811890066
Htr7	2.720804564	4.85E-10	2.59E-09	6.592403571	Pttg1	2.534803734	2.31E-23	1.10E-21	5.794980209
Mip	2.717174898	0.002218232	0.005525957	6.57583864	Alpl	2.527866967	4.51E-06	1.52E-05	5.767183663
Fosl2	2.714127563	6.42E-23	2.80E-21	6.561963475	Zkscan2	2.52717342	0.0016050	0.00406521	5.76441187
Kpna3	2.708109131	7.45E-27	8.87E-25	6.534646215	Nox1	2.5199969	0.0020834	0.005210486	5.735808667
Npr1	2.708098001	3.09E-08	1.31E-07	6.534595803	Car12	2.519994739	0.0004718	0.001266509	5.735800075
Cd80	2.708046378	9.69E-25	6.67E-23	6.534361985	Fcrlb	2.509883829	2.35E-12	1.66E-11	5.695742123
Kynu	2.701083859	4.91E-07	1.82E-06	6.502902796	Tspan3	2.498788406	6.74E-26	6.12E-24	5.652105544
Slfn8	2.700824506	4.89E-27	6.13E-25	6.501733878	Ttc39c	2.49826473	7.42E-25	5.22E-23	5.65005429
Mapkbp1	2.691881453	5.06E-22	1.81E-20	6.46155525	Abracl	2.497790308	8.96E-24	4.85E-22	5.648196607
Cd14	2.690988616	2.40E-27	3.28E-25	6.45755764	Zmynd15	2.496625587	3.20E-21	9.86E-20	5.643638526
Fpr2	2.688408456	9.88E-25	6.76E-23	6.446019066	Ccne1	2.496158935	1.22E-16	1.61E-15	5.641813342
Trpm4	2.687177395	6.69E-22	2.34E-20	6.440520983	Flnb	2.495853206	1.51E-22	5.96E-21	5.640617882
Dusp8	2.681567734	2.67E-11	1.66E-10	6.415526796	Tnc	2.495733324	1.77E-08	7.75E-08	5.64014919
Parp14	2.67896785	4.34E-25	3.21E-23	6.403975772	Tap2	2.491151589	6.08E-22	2.14E-20	5.622265522
Ifi214	2.67896002	3.07E-06	1.05E-05	6.403941014	Ccdc60	2.490545754	0.0003680	0.001000328	5.619905043
Trim21	2.678875382	5.70E-27	6.91E-25	6.403565331	Tex12	2.482006334	0.0007121	0.001874032	5.586738657
Ms4a4a	2.676676468	1.11E-21	3.71E-20	6.393812635	Ccdc149	2.481192192	3.99E-06	1.35E-05	5.583586838
Nod1	2.670570985	1.60E-25	1.32E-23	6.366811208	Rabgef1	2.478338226	2.19E-25	1.75E-23	5.5725522
C130026I21Rik	2.668819085	6.15E-25	4.42E-23	6.359084524	Ddx60	2.469594871	5.98E-25	4.32E-23	5.538882257
March5	2.668378851	9.29E-28	1.46E-25	6.357144363	Trex1	2.455171549	2.27E-25	1.79E-23	5.48378325
Sp6	2.668220852	0.0004789	0.00128452	6.356448189	Bdkrb1	2.451310704	6.31E-05	0.000186978	5.469127531
Nod2	2.666332342	1.22E-22	4.94E-21	6.348132943	Pfkfb3	2.449530554	2.35E-25	1.84E-23	5.462383304
Peli1	2.662514992	1.23E-26	1.36E-24	6.331358079	Ugcg	2.447972152	3.74E-26	3.62E-24	5.456486013
Prrg4	2.660302008	6.72E-05	0.00019860	6.321653704	Stxbp3	2.44512973	3.43E-26	3.37E-24	5.445746137
Il17ra	2.660021009	4.54E-15	4.72E-14	6.320422532	Rtn1	2.443775852	5.55E-06	1.85E-05	5.440638044
Fndc3a	2.660007439	1.03E-25	8.93E-24	6.320363083	Frmd6	2.441612399	1.05E-14	1.03E-13	5.432485428
Lag3	2.659371884	6.54E-08	2.69E-07	6.31757937	Stat5a	2.439924077	1.50E-23	7.57E-22	5.426131747
Casp3	2.657709189	5.65E-23	2.49E-21	6.310302603	Ripk2	2.438809156	3.57E-22	1.31E-20	5.421940028
E2f5	2.655940335	1.52E-18	2.79E-17	6.302570429	Lcp2	2.438580819	5.54E-27	6.79E-25	5.421081959
Svep1	2.655455566	7.38E-10	3.83E-09	6.300453019	Plcd1	2.43353153	2.96E-15	3.17E-14	5.402141877
Rnf144a	2.653704966	1.63E-12	1.18E-11	6.292812537	Ankrd33b	2.430168169	4.32E-23	1.95E-21	5.389562509

Gene	log2 (FC)	P-value	FDR	FC	Gene	log2 (FC)	P-value	FDR	FC
**F11r**	2.429884479	2.88E-14	2.66E-13	5.388502816	**Azi2**	2.27315432	7.59E-26	6.83E-24	4.833788399
**Ifitm1**	2.428479758	4.49E-09	2.11E-08	5.383258702	**Tor1aip2**	2.270975537	3.19E-27	4.22E-25	4.826493838
**Maff**	2.423000273	8.97E-22	3.06E-20	5.36285138	**Cdkn1a**	2.270332943	7.67E-26	6.88E-24	4.824344536
**Plaur**	2.421595662	1.03E-24	6.99E-23	5.357632638	**Plekhh1**	2.270154431	0.0003627	0.000986377	4.823747633
**Olfm1**	2.420982576	3.38E-19	6.99E-18	5.355356349	**Srp54b**	2.268339863	0.0028389	0.006971264	4.817684319
**Fzd1**	2.41935398	1.99E-25	1.61E-23	5.349314331	**Itprip**	2.267616889	2.33E-22	8.93E-21	4.815270648
**Atp2b4**	2.4161064	4.90E-13	3.82E-12	5.337286295	**Map2k1**	2.267592677	3.61E-27	4.69E-25	4.815189836
**Zup1**	2.410282777	2.48E-23	1.17E-21	5.315785081	**Arel1**	2.264863164	1.20E-24	8.04E-23	4.806088332
**Itga9**	2.406922328	1.34E-16	1.75E-15	5.303417508	**Wfdc18**	2.263371383	2.94E-09	1.42E-08	4.801121289
**Olfr1269**	2.406249081	5.39E-08	2.24E-07	5.300943195	**Adgrg7**	2.251699616	8.88E-07	3.21E-06	4.762435706
**Apol7c**	2.405118745	5.23E-10	2.77E-09	5.296791592	**Parp9**	2.25145762	3.16E-26	3.14E-24	4.761636929
**Gpr31b**	2.402019429	3.71E-08	1.57E-07	5.285424803	**Slc7a3**	2.250560833	0.0001216	0.000350144	4.75867799
**Ppp1r16b**	2.402000022	3.48E-12	2.40E-11	5.285353704	**Ctla4**	2.250248387	0.0002688	0.000743747	4.757647507
**Fnbp1l**	2.399607674	2.95E-20	7.55E-19	5.276596533	**Stx11**	2.250061408	2.29E-25	1.80E-23	4.757030936
**Rnf213**	2.399007861	1.14E-19	2.57E-18	5.274403195	**Setdb2**	2.248237223	3.18E-19	6.60E-18	4.751019813
**Gfi1**	2.395063645	1.83E-10	1.03E-09	5.260003082	**Parp10**	2.247474822	6.38E-24	3.59E-22	4.748509771
**Armcx6**	2.391294771	3.83E-11	2.33E-10	5.246279867	**Cacnb3**	2.247156082	3.71E-11	2.26E-10	4.74746078
**Klri2**	2.390571951	3.44E-10	1.86E-09	5.243652031	**Rgs16**	2.246329576	9.73E-07	3.50E-06	4.744741785
**Tpx2**	2.389906447	2.78E-19	5.85E-18	5.24123373	**Dhx58**	2.243933277	8.03E-26	7.16E-24	4.736867368
**Mki67**	2.389814785	1.10E-22	4.56E-21	5.240900737	**Samd9l**	2.243435894	7.42E-24	4.07E-22	4.735234567
**Slamf8**	2.388860063	9.27E-27	1.07E-24	5.237433652	**Zbtb32**	2.240488951	3.28E-09	1.57E-08	4.72557194
**Cp**	2.388238792	6.42E-17	8.89E-16	5.235178732	**Dtx2**	2.240053559	2.24E-23	1.08E-21	4.724146023
**Ptch1**	2.382200085	1.37E-15	1.54E-14	5.213311572	**Olfr539**	2.238847738	7.07E-19	1.37E-17	4.720199176
**Xcr1**	2.380523459	0.0002786	0.00076887	5.207256451	**Stat3**	2.238066513	1.88E-26	1.98E-24	4.717643864
**Naa25**	2.379385066	2.43E-22	9.24E-21	5.203149158	**Mpp2**	2.236935829	1.87E-05	5.88E-05	4.71394795
**Gm19684**	2.379144052	1.19E-12	8.75E-12	5.202280005	**Aftph**	2.236790377	1.54E-24	1.00E-22	4.713472717
**Trim30d**	2.378634445	1.44E-24	9.45E-23	5.200442712	**Akt3**	2.235537947	1.00E-22	4.15E-21	4.709382639
**Znfx1**	2.37776282	2.64E-25	2.02E-23	5.19730174	**Sik3**	2.230467664	1.89E-23	9.29E-22	4.692860789
**Hat1**	2.374287792	2.38E-24	1.47E-22	5.184798032	**Slfn2**	2.230088294	3.29E-26	3.26E-24	4.691626921
**Nr4a1**	2.371496705	1.05E-16	1.41E-15	5.174777042	**Nfat5**	2.228546031	5.15E-14	4.56E-13	4.68661418
**Samsn1**	2.371151555	3.31E-25	2.47E-23	5.173539178	**Gfpt2**	2.228005597	7.08E-15	7.16E-14	4.6848589
**Gpr18**	2.36697383	3.89E-13	3.07E-12	5.158579426	**Abcb1a**	2.225349622	4.42E-18	7.44E-17	4.676242097
**Cx3cl1**	2.366866381	3.24E-05	9.91E-05	5.158195239	**Rffl**	2.225204161	9.18E-24	4.93E-22	4.675770631
**Pde10a**	2.365206558	8.77E-10	4.51E-09	5.15226414	**Ms4a4b**	2.223500327	1.23E-09	6.17E-09	4.670251771
**Hspa1l**	2.358973235	9.31E-14	7.96E-13	5.130051238	**Rapgef2**	2.222779988	4.91E-22	1.77E-20	4.667920493
**Fam71f2**	2.358160479	0.0025059	0.00619478	5.127161989	**Stxbp1**	2.22268691	7.67E-22	2.63E-20	4.667619343
**Sfrp1**	2.356266168	0.0013243	0.00339129	5.120434256	**Eif2ak2**	2.217564566	1.51E-24	9.91E-23	4.651076169
**Notch1**	2.355296344	1.25E-19	2.80E-18	5.116993297	**Gramd1a**	2.215476946	1.93E-23	9.46E-22	4.644350797
**Scube3**	2.354026663	0.0002095	0.00058734	5.112491939	**Fam83c**	2.2151301	0.0002094	0.000587025	4.643234357
**Lysmd2**	2.353348024	2.05E-05	6.42E-05	5.110087605	**D6Ertd527e**	2.210735887	0.0026442	0.006517813	4.629113344
**Pi4k2b**	2.347612359	8.55E-23	3.63E-21	5.089811966	**Bpifc**	2.209076347	1.08E-07	4.31E-07	4.62379151
**Nfkb1**	2.345528451	2.45E-25	1.90E-23	5.082465267	**Tlr2**	2.20810884	1.74E-27	2.50E-25	4.620691721
**Olfr735**	2.344085838	4.86E-14	4.33E-13	5.077385633	**Ogfr**	2.207218897	6.93E-25	4.95E-23	4.617842274
**St3gal1**	2.342717487	2.32E-20	6.03E-19	5.072572175	**Gpr85**	2.204393205	3.38E-14	3.09E-13	4.608806524
**Olfr1122**	2.341804035	2.28E-12	1.61E-11	5.069361457	**Rbl1**	2.203022687	1.13E-23	5.94E-22	4.604430371
**Ccl9**	2.340058077	1.32E-23	6.80E-22	5.063230197	**Whamm**	2.201456885	5.30E-21	1.57E-19	4.59943575
**Rab3ip**	2.338902108	8.21E-21	2.33E-19	5.059174874	**Tet1**	2.199299024	5.64E-05	0.000168152	4.592561445
**Rnd3**	2.338429911	4.44E-23	2.00E-21	5.057519269	**Ext1**	2.19909771	3.68E-22	1.35E-20	4.591920643
**Ddit4**	2.335024455	9.54E-17	1.28E-15	5.045595128	**Tmco3**	2.195973212	4.80E-23	2.13E-21	4.581986512
**H2-Q7**	2.334029159	1.58E-25	1.31E-23	5.042115475	**Aldh1a2**	2.195732544	2.94E-20	7.53E-19	4.581222217
**Plagl2**	2.332521894	7.06E-24	3.90E-22	5.036850443	**D16Ertd472e**	2.194199245	4.43E-19	8.98E-18	4.576355873
**Oas1a**	2.329729543	6.53E-26	5.95E-24	5.027110996	**Ehd3**	2.193574443	6.02E-08	2.48E-07	4.574374373
**Samd11**	2.327754089	5.36E-06	1.79E-05	5.02023218	**Errfi1**	2.190851109	9.10E-20	2.09E-18	4.565747604
**Gm8369**	2.326269695	3.92E-07	1.47E-06	5.015069505	**Bst2**	2.189088179	1.69E-23	8.40E-22	4.560171805
**Samhd1**	2.325434576	6.63E-28	1.11E-25	5.012167318	**Tnfrsf4**	2.18873962	5.44E-05	0.000162795	4.559070187
**Hif1a**	2.324730974	1.35E-25	1.14E-23	5.009723479	**Ccng2**	2.188491713	9.55E-21	2.68E-19	4.558286843
**Stk38l**	2.324141329	4.08E-19	8.33E-18	5.007676371	**Tlr7**	2.187880879	2.12E-25	1.69E-23	4.556357283
**Trim30a**	2.3238305	2.09E-26	2.15E-24	5.006597584	**Clec4e**	2.185596795	7.18E-26	6.49E-24	4.549149337
**Tent5a**	2.32171135	2.05E-24	1.30E-22	4.999248875	**Dusp5**	2.185476215	1.64E-18	2.99E-17	4.548769134
**Nfkbia**	2.320699393	1.71E-25	1.41E-23	4.995743457	**AC168977.1**	2.185089782	0.000492336	0.001319081	4.547550887
**Ramp3**	2.319972199	3.93E-12	2.70E-11	4.993225975	**Csrp1**	2.184767303	3.16E-22	1.18E-20	4.546534509
**Galnt18**	2.314786055	0.000123193	0.000354504	4.975308743	**Rhoh**	2.180283562	3.72E-18	6.37E-17	4.532426303
**Prf1**	2.313283391	1.92E-08	8.41E-08	4.970129321	**Gjb2**	2.180154975	0.0002834	0.000783133	4.532022347
**Cdk5r1**	2.308671429	7.89E-10	4.09E-09	4.954266338	**Tent4a**	2.180087077	2.40E-22	9.15E-21	4.531809059
**Bcl2a1b**	2.308434471	8.46E-26	7.52E-24	4.953452681	**Prdm1**	2.180031273	1.39E-22	5.61E-21	4.531633771
**Dab2**	2.305502084	1.18E-24	7.93E-23	4.943394641	**Psmb10**	2.179260475	1.39E-23	7.10E-22	4.529213273
**Dtx3l**	2.303974367	2.65E-24	1.60E-22	4.938162689	**Nrp2**	2.176720956	1.72E-20	4.65E-19	4.521247692
**Serpinb9**	2.303837358	1.37E-21	4.56E-20	4.937693745	**Itga2**	2.174656256	9.50E-07	3.42E-06	4.514781775
**Parp11**	2.302755186	6.92E-24	3.84E-22	4.933991348	**Ms4a6d**	2.173408401	1.98E-26	2.06E-24	4.510878417
**Itpr1**	2.300770185	5.99E-21	1.75E-19	4.927207349	**Gprc5c**	2.170147275	7.94E-07	2.88E-06	4.50069336
**P3h2**	2.29570004	7.81E-05	0.000229368	4.909921775	**Il12rb1**	2.169527347	2.75E-08	1.18E-07	4.498759821
**Cd83**	2.289061509	5.48E-20	1.33E-18	4.887380776	**Pml**	2.162740272	1.53E-23	7.68E-22	4.477645373
**Scarf1**	2.288509958	8.39E-23	3.57E-21	4.885512656	**Coro2a**	2.162258292	5.37E-26	5.00E-24	4.476149716
**C6**	2.28764895	0.001897893	0.004768483	4.882597826	**Zc3hav1l**	2.15934643	9.16E-07	3.31E-06	4.467124395
**Epsti1**	2.285728929	3.96E-24	2.32E-22	4.876104107	**Cxcl16**	2.15512256	2.43E-25	1.89E-23	4.454064839
**Dusp2**	2.281797875	1.13E-19	2.56E-18	4.862835789	**Inpp5b**	2.152339328	3.96E-24	2.32E-22	4.445480388
**Mitd1**	2.277404175	5.26E-20	1.28E-18	4.848048645	**Ubtd2**	2.151457877	3.13E-13	2.50E-12	4.442765139
**Csgalnact1**	2.276413934	0.0038177	0.00922133	4.844722171	**Olfr975**	2.148841081	4.51E-05	0.000135892	4.434714045
**Lap3**	2.274641593	1.33E-23	6.83E-22	4.838774116	**Asb11**	2.148220713	0.0013214	0.003384371	4.432807501
**Tfpi2**	2.274564784	1.65E-05	5.22E-05	4.838516508	**Carhsp1**	2.141897545	2.48E-24	1.51E-22	4.413421524
**Slc2a1**	2.273884901	9.25E-24	4.95E-22	4.836236851	**Cxcl5**	2.139448696	5.47E-16	6.54E-15	4.40593648

Gene	log2 (FC)	P-value	FDR	FC	Gene	log2 (FC)	P-value	FDR	FC
**Trim12a**	2.138324652	6.72E-22	2.34E-20	4.402505028	**Mthfr**	1.99422986	1.65E-23	8.23E-22	3.984033724
**Tgm2**	2.136779024	2.51E-27	3.41E-25	4.397790942	**Arid5a**	1.993434817	5.00E-18	8.27E-17	3.981838802
**Ccdc25**	2.135486958	1.90E-21	6.16E-20	4.393854079	**Sppl2a**	1.992901733	4.61E-25	3.39E-23	3.980367762
**Cd200**	2.131112271	1.16E-25	9.86E-24	4.380550765	**Gm4070**	1.991534237	5.84E-21	1.71E-19	3.976596655
**Wars**	2.129703798	2.29E-26	2.33E-24	4.376276214	**Lnpk**	1.987294521	1.57E-18	2.88E-17	3.964927597
**Pcgf5**	2.125619765	4.58E-23	2.05E-21	4.363905211	**Lrp11**	1.986840071	5.52E-20	1.34E-18	3.963678837
**Mark1**	2.119929212	0.0020487	0.00512829	4.346726167	**Jarid2**	1.986757201	9.74E-23	4.07E-21	3.963451165
**Sipa1l1**	2.118616811	7.39E-19	1.42E-17	4.342773802	**Ms4a6c**	1.986516228	5.19E-25	3.79E-23	3.962789208
**Ehd1**	2.115797731	1.08E-25	9.26E-24	4.334296148	**Dmwd**	1.986277915	2.12E-06	7.38E-06	3.962134664
**Rnf24**	2.114782871	1.04E-14	1.03E-13	4.33124827	**Tor3a**	1.985640714	4.89E-25	3.58E-23	3.960385078
**Tcf4**	2.111594497	6.75E-21	1.96E-19	4.321686727	**P4ha2**	1.984891133	1.78E-12	1.28E-11	3.958327915
**Vmn2r53**	2.109064073	0.0005693	0.00151398	4.314113321	**Col5a3**	1.984376402	2.60E-07	9.98E-07	3.956915898
**Dbnl**	2.106925222	6.38E-26	5.84E-24	4.307722219	**Cth**	1.984171878	0.0004408	0.001187592	3.956354985
**Tnfrsf1b**	2.106786402	6.55E-28	1.11E-25	4.307307741	**Arl4a**	1.982431385	9.42E-17	1.27E-15	3.951584846
**Creb5**	2.102156119	4.98E-20	1.22E-18	4.293505735	**Aida**	1.979565175	1.99E-19	4.30E-18	3.943742003
**Atf3**	2.100221689	2.24E-26	2.29E-24	4.287752669	**Psd4**	1.979539293	5.84E-24	3.30E-22	3.943671255
**Stat2**	2.100015103	6.15E-25	4.42E-23	4.28713873	**Grap**	1.979487629	2.44E-15	2.65E-14	3.94353003
**Hk2**	2.0997589	1.00E-24	6.80E-23	4.286377461	**Il4ra**	1.978010467	1.94E-20	5.18E-19	3.939494354
**Selenow**	2.096018649	1.57E-21	5.13E-20	4.275279229	**Cd164**	1.976384468	1.86E-23	9.15E-22	3.935056821
**Cpeb4**	2.093888995	2.57E-21	8.10E-20	4.268972873	**Adar**	1.976241632	4.43E-25	3.27E-23	3.934667245
**Batf**	2.093194386	2.77E-20	7.14E-19	4.266918001	**Ppp4r2**	1.97366338	9.87E-24	5.25E-22	3.927641848
**Il18bp**	2.092260031	2.77E-21	8.67E-20	4.264155445	**Intu**	1.972981036	9.05E-18	1.45E-16	3.925784651
**Ubr4**	2.090278697	3.50E-18	6.03E-17	4.25830326	**Rgs14**	1.971991222	1.33E-23	6.85E-22	3.923092146
**Lgals9**	2.0884198	2.44E-25	1.90E-23	4.252820017	**Inava**	1.969813357	9.81E-06	3.18E-05	3.917174387
**Rnf135**	2.086671708	6.34E-20	1.52E-18	4.24767006	**Ccno**	1.969642115	0.0012454	0.003199086	3.916709462
**Pkp4**	2.084966188	6.70E-18	1.09E-16	4.242651532	**Adamts9**	1.969398887	5.10E-07	1.89E-06	3.916049191
**Spryd7**	2.084735893	2.24E-24	1.40E-22	4.241974336	**Slc39a14**	1.968848009	7.78E-22	2.66E-20	3.914554173
**Denr**	2.084426324	4.51E-23	2.03E-21	4.241064206	**Msi2**	1.967741503	3.55E-18	6.10E-17	3.911552973
**Gm21897**	2.083317756	9.11E-10	4.67E-09	4.237806619	**Zfp382**	1.96770758	2.06E-11	1.29E-10	3.911460999
**Oaf**	2.080739317	8.37E-18	1.34E-16	4.230239417	**Magohb**	1.966938682	1.52E-14	1.46E-13	3.909376905
**Gimap6**	2.07950305	6.84E-15	6.93E-14	4.226616014	**Tlk2**	1.966548199	4.32E-23	1.95E-21	3.908318928
**Prpf38a**	2.078815133	2.08E-21	6.68E-20	4.224601127	**Heg1**	1.965690572	4.55E-18	7.63E-17	3.90599627
**Olfr571**	2.077571814	4.21E-09	1.99E-08	4.22096192	**Trim25**	1.964202615	1.12E-23	5.89E-22	3.901969807
**Olfr1167**	2.076589991	0.0006726	0.00177433	4.21809033	**Ier2**	1.963834132	1.90E-20	5.09E-19	3.900973322
**Cxcr5**	2.076240688	4.46E-10	2.39E-09	4.217069179	**Tal1**	1.962697795	2.83E-13	2.27E-12	3.897901933
**Fasl**	2.074555991	0.0007963	0.00208743	4.2121476	**Cdc42ep2**	1.960997742	3.86E-24	2.27E-22	3.893311403
**Stfa3**	2.073572386	4.94E-09	2.31E-08	4.209276805	**Traf4**	1.958196446	1.45E-07	5.69E-07	3.885759054
**Sp100**	2.073058418	1.04E-25	9.00E-24	4.207777496	**Nes**	1.95818819	3.14E-09	1.51E-08	3.885736819
**Gm7609**	2.072747851	1.63E-09	8.09E-09	4.20687179	**Junb**	1.956967373	7.87E-25	5.50E-23	3.882450076
**Oasl2**	2.072699457	2.86E-25	2.17E-23	4.206730678	**Tnfsf11**	1.955938358	6.11E-05	0.000181516	3.87968187
**Tmem243**	2.066122621	1.28E-20	3.53E-19	4.187597036	**Gjb4**	1.955724202	3.78E-05	0.000114758	3.879106008
**Rmdn3**	2.065916159	5.81E-24	3.30E-22	4.186997798	**Dcp2**	1.955571713	7.45E-21	2.14E-19	3.878696018
**Mgat4a**	2.064376845	1.13E-22	4.68E-21	4.182532774	**Rbms2**	1.953892574	2.33E-17	3.50E-16	3.874184266
**Itga5**	2.062606766	2.58E-26	2.61E-24	4.177404265	**Plk2**	1.951661095	2.57E-25	1.98E-23	3.868196528
**Pla2g4a**	2.062114397	6.60E-24	3.69E-22	4.175978827	**Ahr**	1.950754527	8.46E-20	1.97E-18	3.865766577
**Tmem184b**	2.059249874	8.72E-25	6.04E-23	4.167695496	**Lyrm1**	1.944601089	3.46E-16	4.22E-15	3.849313274
**Stat4**	2.057229739	6.68E-16	7.88E-15	4.16186376	**Grasp**	1.943425273	1.57E-05	4.98E-05	3.846177311
**Fcgr1**	2.053224799	1.65E-24	1.07E-22	4.150326396	**Mdm2**	1.943133314	5.83E-24	3.30E-22	3.845399037
**Psme1**	2.051889291	1.41E-24	9.31E-23	4.1464862	**Mllt6**	1.941133925	2.03E-20	5.40E-19	3.8400735
**Dgkh**	2.049847596	1.61E-16	2.09E-15	4.140622263	**Tapbp**	1.940516474	1.53E-24	1.00E-22	3.838430361
**Fhl3**	2.048926664	1.79E-22	7.00E-21	4.137979975	**Ets2**	1.939499727	1.16E-25	9.86E-24	3.835726158
**Ube2l6**	2.048803321	3.23E-25	2.42E-23	4.137626215	**Rnf31**	1.938329637	2.10E-23	1.02E-21	3.832616476
**Tmcc3**	2.047950524	5.24E-21	1.55E-19	4.135181131	**Shfl**	1.938162405	6.32E-10	3.32E-09	3.83217224
**Dennd3**	2.047119273	7.25E-19	1.40E-17	4.132799211	**Prrx1**	1.935666192	1.05E-05	3.40E-05	3.825547382
**Parp8**	2.044662685	7.10E-22	2.46E-20	4.125767964	**Vmn1r6**	1.934699739	0.000933968	0.002431411	3.822985528
**Zfp800**	2.04303113	5.66E-19	1.11E-17	4.121104738	**Rnaset2b**	1.933679965	1.19E-22	4.90E-21	3.820284194
**Fpr3**	2.039775177	0.003486479	0.008462703	4.11181449	**Tet2**	1.929147715	4.38E-14	3.93E-13	3.80830154
**Rhbdf2**	2.034065886	1.58E-23	7.91E-22	4.095574637	**Mafk**	1.923941149	5.65E-22	2.01E-20	3.794582466
**Oas2**	2.033135744	1.10E-20	3.07E-19	4.092934966	**A4galt**	1.923611954	0.001835931	0.004620834	3.793716716
**Adcy6**	2.031558099	2.72E-07	1.04E-06	4.088461624	**Pim3**	1.923433746	2.08E-20	5.49E-19	3.793248127
**Frmd4a**	2.031469067	2.81E-23	1.31E-21	4.088209324	**Stxbp6**	1.923201267	9.43E-11	5.48E-10	3.792636926
**Calcrl**	2.02814967	3.79E-21	1.15E-19	4.078813861	**Ly6c2**	1.919131114	6.04E-19	1.18E-17	3.781952159
**Bcl2l14**	2.028132763	1.64E-05	5.18E-05	4.07876606	**Prdx5**	1.918686968	4.37E-21	1.31E-19	3.780788033
**Eno2**	2.028121191	9.72E-14	8.27E-13	4.078733344	**Psme2**	1.915602263	3.46E-22	1.28E-20	3.772712761
**Mier3**	2.02798085	9.40E-22	3.19E-20	4.078336595	**Smox**	1.91248927	2.37E-22	9.03E-21	3.764580919
**Mtmr14**	2.023383313	1.50E-23	7.56E-22	4.065360562	**Chst11**	1.909655668	3.63E-22	1.33E-20	3.757194149
**Hook2**	2.022762153	5.11E-20	1.24E-18	4.063610577	**Olfr107**	1.906890964	0.0001000	0.000290586	3.750000957
**Gm10053**	2.022086436	7.43E-22	2.56E-20	4.061707745	**Zfp658**	1.906638419	6.24E-16	7.40E-15	3.749344575
**Sec24b**	2.021367185	1.13E-23	5.93E-22	4.059683297	**Spred1**	1.9059878	1.49E-22	5.92E-21	3.747654095
**Dhx40**	2.016767753	3.59E-22	1.32E-20	4.046761298	**Ckap2l**	1.904267372	9.82E-09	4.45E-08	3.743187645
**Slc12a4**	2.009029384	1.98E-24	1.26E-22	4.025113275	**Sco1**	1.902631329	3.67E-18	6.31E-17	3.738945207
**Fcgr4**	2.007683342	1.47E-22	5.85E-21	4.021359573	**Lancl2**	1.901541557	4.14E-17	5.94E-16	3.736121977
**Ralgds**	2.005400069	3.48E-26	3.40E-24	4.015000226	**Casp1**	1.901022719	3.20E-24	1.91E-22	3.734778592
**Olfr509**	2.004592353	1.52E-09	7.57E-09	4.012752993	**Ppa1**	1.900412931	3.33E-21	1.02E-19	3.733200338
**Nipal1**	2.003714445	0.00012931	0.000371074	4.010311899	**Psmb9**	1.899994284	2.78E-22	1.04E-20	3.732117178
**Pdzk1ip1**	2.000129989	6.85E-05	0.00020224	4.000360421	**Olfr46**	1.899741412	2.54E-11	1.58E-10	3.73146308
**Tes**	2.000088156	2.14E-23	1.03E-21	4.000244427	**Dpp10**	1.899396693	5.85E-09	2.72E-08	3.730571587
**Serpine2**	1.999797028	0.000323006	0.000883194	3.999437282	**Hmga2**	1.897062268	9.26E-14	7.92E-13	3.724540029
**Dcun1d3**	1.997724434	6.64E-21	1.93E-19	3.993695765	**Mmp2**	1.896639679	2.32E-11	1.45E-10	3.723449213
**Trafd1**	1.996781958	4.50E-26	4.24E-24	3.991087637	**Pnpt1**	1.893831223	1.73E-22	6.79E-21	3.716207923
**Gvin1**	1.996780781	7.34E-21	2.11E-19	3.991084379	**Il2rb**	1.892723439	3.29E-12	2.28E-11	3.713355501

Gene	log2 (FC)	P-value	FDR	FC	Gene	log2 (FC)	P-value	FDR	FC
**Gm21833**	1.892411199	1.52E-05	4.81E-05	3.712551913	**Bean1**	1.759849601	0.0027824	0.006838563	3.386628179
**Asah2**	1.889753205	1.65E-18	3.00E-17	3.705718274	**Tnfaip8l3**	1.758819267	5.35E-08	2.22E-07	3.384210404
**Nxpe3**	1.888117344	2.76E-09	1.34E-08	3.701518768	**Dync1i2**	1.758552247	2.47E-23	1.17E-21	3.3835841
**Tmem229b**	1.884933733	1.49E-21	4.90E-20	3.693359594	**Gimap9**	1.758099758	2.27E-12	1.61E-11	3.382523032
**Casp8**	1.883434537	8.52E-25	5.92E-23	3.689523583	**Git1**	1.75596693	6.43E-23	2.80E-21	3.377526128
**Slfn5**	1.883015247	1.40E-19	3.13E-18	3.688451453	**Nectin2**	1.755361463	2.69E-12	1.89E-11	3.376108953
**Tnfrsf25**	1.882895911	3.35E-05	0.00010228	3.688146367	**Phlpp2**	1.755070449	7.77E-13	5.87E-12	3.375428007
**Saa3**	1.880005145	6.76E-19	1.31E-17	3.680763728	**Stx2**	1.754610377	3.33E-21	1.02E-19	3.374351763
**Ttc9c**	1.879559049	5.07E-23	2.24E-21	3.679625774	**Larp1**	1.752659197	9.73E-20	2.23E-18	3.369791189
**Slc26a1**	1.872253257	0.0033953	0.00825424	3.661039291	**Gm6034**	1.752283356	0.0005201	0.001389833	3.368913428
**Marf1**	1.871158037	1.42E-19	3.16E-18	3.658261072	**Ptpn2**	1.751619258	3.51E-17	5.11E-16	3.367363016
**Ascc3**	1.869487628	8.56E-20	1.98E-18	3.654027846	**Filip1l**	1.751344737	1.80E-20	4.84E-19	3.366722324
**Ktn1**	1.868942218	3.03E-19	6.33E-18	3.652646703	**Notch2**	1.750286311	8.03E-18	1.29E-16	3.364253251
**Aff1**	1.868716539	1.57E-18	2.87E-17	3.65207537	**Elp5**	1.749571118	8.64E-17	1.17E-15	3.362585889
**Rnf145**	1.867759322	5.93E-22	2.10E-20	3.64965305	**Dnajb6**	1.747575722	3.51E-23	1.60E-21	3.357938301
**Phlpp1**	1.867109829	1.24E-19	2.78E-18	3.648010366	**Tex14**	1.746930755	3.35E-07	1.27E-06	3.356437446
**Rnf114**	1.863353411	7.68E-22	2.63E-20	3.638524212	**Tnip3**	1.746533657	8.16E-20	1.91E-18	3.355513723
**Igsf9**	1.861771445	1.06E-08	4.79E-08	3.63453663	**Man2a1**	1.745558758	5.28E-21	1.56E-19	3.353247005
**Slx4ip**	1.861051689	4.37E-10	2.34E-09	3.632723821	**Pilra**	1.743589624	1.32E-22	5.35E-21	3.348673282
**Csf3r**	1.860170008	1.15E-23	6.03E-22	3.630504418	**Adap2**	1.74336046	2.16E-18	3.84E-17	3.348141405
**C2**	1.859327954	1.29E-12	9.45E-12	3.628386028	**Ranbp2**	1.741071081	4.82E-19	9.66E-18	3.342832531
**Dusp1**	1.858428145	2.11E-22	8.20E-21	3.626123708	**Bmp6**	1.741002478	0.0037302	0.009018863	3.342673577
**Acpp**	1.851681695	1.88E-18	3.38E-17	3.609206514	**Il10ra**	1.74006376	1.49E-23	7.56E-22	3.340499308
**Nfkbid**	1.850065077	4.98E-19	9.95E-18	3.605164469	**Klk9**	1.732496355	6.49E-05	0.000192159	3.323023177
**Olfr102**	1.849375014	0.0008087	0.00211835	3.603440477	**Psme2b**	1.730568393	6.07E-12	4.06E-11	3.318585383
**Ttc39b**	1.848205681	4.03E-21	1.21E-19	3.600520999	**Ankrd17**	1.728414667	2.00E-19	4.31E-18	3.313634931
**Psmb8**	1.848002665	6.38E-22	2.24E-20	3.60001437	**Tiparp**	1.726166004	3.14E-22	1.17E-20	3.308474141
**Bcl9**	1.847421242	1.33E-16	1.74E-15	3.598563815	**Ackr3**	1.72180343	0.0032313	0.007882065	3.298484738
**Slc9b2**	1.846944185	8.18E-06	2.67E-05	3.597374071	**Steap1**	1.721512386	0.0003328	0.000909194	3.297819381
**Tm4sf1**	1.846466483	7.01E-07	2.56E-06	3.596183113	**Lztfl1**	1.720525341	3.62E-19	7.45E-18	3.295563894
**Tnfrsf18**	1.844921324	0.0004751	0.00127506	3.592333581	**Foxq1**	1.719552297	0.0039951	0.009624133	3.293341907
**Ly86**	1.841905609	9.80E-23	4.08E-21	3.584832246	**Dnah17**	1.719524024	6.50E-07	2.38E-06	3.293277367
**Rilpl1**	1.840110041	1.88E-14	1.78E-13	3.580373365	**Riox2**	1.718591279	4.59E-22	1.66E-20	3.291148854
**Itk**	1.8395453	4.88E-08	2.04E-07	3.578972108	**Olr1**	1.716717077	2.33E-17	3.50E-16	3.286876107
**Btla**	1.838526268	1.20E-08	5.37E-08	3.576445031	**Alas1**	1.715863594	1.61E-20	4.38E-19	3.284932201
**Gdf15**	1.837178147	6.44E-22	2.26E-20	3.573104597	**Tspo**	1.715133551	6.93E-22	2.40E-20	3.283270355
**Perm1**	1.83672344	9.12E-16	1.05E-14	3.571978606	**Wdr59**	1.713065175	5.86E-18	9.62E-17	3.278566539
**Zbtb46**	1.835147423	3.26E-09	1.56E-08	3.568078666	**Reg2**	1.711838772	3.85E-05	0.000116698	3.275780686
**Gpr34**	1.83476347	0.0038386	0.00926868	3.567129197	**Reps1**	1.710231339	5.51E-22	1.96E-20	3.272132885
**Dusp10**	1.834163788	2.21E-12	1.57E-11	3.565646764	**Svbp**	1.710166532	5.15E-17	7.26E-16	3.271985901
**Chmp4b**	1.833441442	3.42E-23	1.56E-21	3.563861922	**Lrrc63**	1.709498617	9.83E-05	0.000285802	3.270471442
**Itga7**	1.832069955	0.0013189	0.00337882	3.560475574	**Dpysl3**	1.707850645	3.91E-10	2.11E-09	3.266737757
**Cd163l1**	1.827318558	0.0011881	0.00305954	3.54876873	**Olfr344**	1.706160113	1.56E-07	6.13E-07	3.262912078
**Crem**	1.82542228	6.71E-17	9.25E-16	3.544107292	**Pygm**	1.705059902	0.0003200	0.000876018	3.260424703
**Cmtr1**	1.821290734	3.93E-22	1.43E-20	3.533972305	**Crybg1**	1.704278341	2.29E-21	7.28E-20	3.258658888
**Dnase1l3**	1.817834953	3.22E-05	9.86E-05	3.525517283	**AC147806.2**	1.702273507	4.20E-15	4.38E-14	3.254133656
**Spsb1**	1.816903188	9.54E-11	5.54E-10	3.523241062	**Amigo2**	1.702184262	3.14E-05	9.63E-05	3.253932361
**Coa5**	1.815217727	2.64E-24	1.60E-22	3.519127359	**Olfr433**	1.701392712	5.15E-06	1.73E-05	3.252147546
**Rep15**	1.814201682	0.000227249	0.000634325	3.516649822	**Stard3**	1.701124478	3.37E-24	2.00E-22	3.251542943
**Tlr11**	1.813095768	3.43E-05	0.00010478	3.513955125	**Mgst2**	1.699650195	0.0001382	0.000395331	3.248221906
**Rnf19b**	1.812573561	7.24E-24	4.00E-22	3.512683423	**Fam53c**	1.696571698	9.98E-20	2.28E-18	3.241298072
**2700081O15Rik**	1.81242388	2.77E-08	1.18E-07	3.512318998	**Usp12**	1.695861723	2.49E-23	1.18E-21	3.239703367
**Gpr171**	1.811517795	2.82E-05	8.68E-05	3.510113777	**Rap2c**	1.694134014	2.26E-22	8.69E-21	3.23582596
**Gimap5**	1.807924869	6.01E-06	1.99E-05	3.501382973	**Smg7**	1.693080843	1.86E-20	4.99E-19	3.233464661
**Triobp**	1.807488382	7.73E-24	4.23E-22	3.500323791	**Plpp3**	1.691671221	7.96E-23	3.44E-21	3.230306864
**Klra2**	1.80453421	2.53E-16	3.18E-15	3.493163595	**Igf2bp2**	1.69085362	3.90E-11	2.37E-10	3.228476711
**Slc35g2**	1.803374846	0.002315014	0.005754494	3.490357583	**Bfar**	1.689680606	2.28E-22	8.75E-21	3.225852795
**Myo1d**	1.799149934	9.96E-10	5.08E-09	3.480151071	**Tlr1**	1.685274269	3.25E-20	8.23E-19	3.216015297
**Usp25**	1.7984052	2.98E-23	1.39E-21	3.478355044	**9930111J21Rik1**	1.682714047	5.12E-13	3.98E-12	3.210313183
**Stat1**	1.797625556	9.10E-26	8.02E-24	3.476475823	**Zbtb5**	1.682337104	4.21E-18	7.12E-17	3.209474513
**Acsl1**	1.796917135	2.20E-25	1.75E-23	3.474769152	**Gbp2b**	1.680115018	0.0001554	0.000441723	3.204534981
**Hsp90aa1**	1.796586014	2.70E-23	1.27E-21	3.473971731	**Vps54**	1.678367065	3.05E-21	9.47E-20	3.200654752
**Psma5**	1.795366969	3.38E-23	1.55E-21	3.471037542	**Slfn1**	1.678054841	6.44E-24	3.61E-22	3.199962151
**P4ha1**	1.791606454	5.04E-24	2.90E-22	3.462001749	**Lrrc32**	1.677398418	5.09E-12	3.45E-11	3.198506507
**Gm42517**	1.789078418	6.73E-09	3.10E-08	3.455940594	**Nbas**	1.676634422	4.91E-18	8.15E-17	3.196813147
**Akap13**	1.786954368	9.31E-20	2.14E-18	3.450856227	**Hivep1**	1.675541702	4.05E-19	8.29E-18	3.194392747
**Tra2a**	1.786728201	2.12E-21	6.80E-20	3.450315289	**Glrp1**	1.674094823	1.06E-14	1.05E-13	3.191190698
**Gm8113**	1.786567987	3.57E-22	1.31E-20	3.449932146	**Il18rap**	1.673964893	1.15E-07	4.58E-07	3.19090331
**Gstt1**	1.781824102	1.50E-05	4.75E-05	3.438606674	**Pdlim7**	1.673763268	1.99E-13	1.63E-12	3.190457393
**Csprs**	1.781649363	3.69E-10	1.99E-09	3.438190216	**Pglyrp2**	1.673609765	0.0001180	0.000340386	3.190117946
**St3gal3**	1.77913347	4.37E-20	1.09E-18	3.432199635	**Snai1**	1.666355901	2.38E-05	7.40E-05	3.174118304
**H2-Q4**	1.779058653	1.12E-23	5.90E-22	3.432021648	**Ly6d**	1.66615791	1.96E-08	8.55E-08	3.173682729
**Sptbn2**	1.777252875	1.20E-06	4.27E-06	3.427728578	**Spata13**	1.663906681	6.88E-16	8.10E-15	3.168734271
**1110038F14Rik**	1.776326502	5.00E-17	7.06E-16	3.425528296	**Slc30a4**	1.661025576	4.75E-15	4.93E-14	3.162412529
**Max**	1.774940388	2.12E-24	1.34E-22	3.422238694	**Cycs**	1.660919572	2.05E-20	5.44E-19	3.162180174
**Larp1b**	1.77322451	1.18E-18	2.21E-17	3.418170852	**Bcl2a1a**	1.660213913	1.47E-22	5.87E-21	3.16063385
**P2ry10**	1.771831193	4.41E-09	2.08E-08	3.414871266	**Sesn3**	1.659945392	3.24E-12	2.25E-11	3.160045633
**Sgcb**	1.770788973	9.80E-22	3.31E-20	3.412405215	**Psma3**	1.659022847	4.70E-10	2.50E-09	3.158025559
**Rel**	1.768260036	7.24E-21	2.09E-19	3.40642876	**Tph1**	1.657731454	4.76E-09	2.23E-08	3.155199994
**Prkx**	1.766712137	2.64E-22	9.97E-21	3.402775889	**Iqsec2**	1.657412271	2.33E-16	2.94E-15	3.154502012
**Klf8**	1.765435393	1.32E-14	1.28E-13	3.399765862	**Tor1aip1**	1.65679564	2.39E-24	1.47E-22	3.153154014

Gene	log2 (FC)	P-value	FDR	FC	Gene	log2 (FC)	P-value	FDR	FC
**BC035044**	1.655386908	0.002605276	0.006425912	3.150076593	**Sorl1**	-1.60788183	3.30E-16	4.04E-15	0.328079685
**Slc6a12**	1.65528253	2.75E-07	1.05E-06	3.149848697	**Nphp3**	-1.609584417	5.43E-06	1.81E-05	0.327692733
**Csf1**	1.653482338	1.20E-22	4.90E-21	3.145920772	**Shtn1**	-1.60977078	8.92E-17	1.20E-15	0.327650405
**Gsdmd**	1.650714784	1.49E-21	4.90E-20	3.139891667	**Plcb1**	-1.609976751	8.36E-09	3.83E-08	0.32760363
**Vmn1r229**	1.650709506	2.12E-14	2.00E-13	3.13988018	**Wdr76**	-1.610006562	3.66E-08	1.54E-07	0.327596861
**Plac8**	1.650518825	4.93E-12	3.34E-11	3.13946521	**Cep295nl**	-1.610244173	0.003903385	0.009416677	0.32754291
**Mmp14**	1.649038621	1.45E-22	5.80E-21	3.136245773	**Pdf**	-1.611235913	5.05E-13	3.93E-12	0.327317828
**Gpd2**	1.648750231	1.71E-20	4.64E-19	3.135618911	**Fgd2**	-1.613488316	0.001588941	0.004025354	0.326807202
**Hk3**	1.642827516	9.97E-23	4.14E-21	3.1227726	**Tfrc**	-1.614250726	2.48E-21	7.84E-20	0.326634543
**Fstl1**	1.642380448	2.09E-23	1.02E-21	3.121805053	**Gm14440**	-1.615391893	1.15E-07	4.59E-07	0.326376278
**Shf**	1.642015327	2.22E-09	1.09E-08	3.121015077	**Tmem106c**	-1.616189148	1.03E-14	1.02E-13	0.326195967
**St6galnac4**	1.640195332	1.00E-22	4.15E-21	3.117080324	**Bpgm**	-1.616272071	5.09E-11	3.05E-10	0.326177219
**Cldnd1**	1.639655512	2.29E-20	5.98E-19	3.11591421	**Xk**	-1.616577796	0.0005864	0.001557893	0.326108105
**Trim26**	1.638227406	9.14E-19	1.74E-17	3.11283133	**Cercam**	-1.617584448	4.55E-09	2.14E-08	0.32588064
**Irf4**	1.635685337	3.29E-14	3.01E-13	3.107351264	**Pnkd**	-1.61807171	5.68E-13	4.38E-12	0.325770594
**Ift22**	1.633084701	1.19E-19	2.68E-18	3.101754924	**B3gntl1**	-1.620832426	2.19E-07	8.46E-07	0.325147801
**Sema3c**	1.632948095	8.99E-09	4.09E-08	3.101461239	**Wrb**	-1.620956706	2.05E-12	1.46E-11	0.325119793
**Ccnyl1**	1.632464714	7.86E-17	1.07E-15	3.100422257	**Ctns**	-1.621940268	1.72E-17	2.64E-16	0.324898217
**Tmed5**	1.630075802	2.22E-19	4.76E-18	3.095292616	**Jmjd8**	-1.623326779	4.65E-15	4.83E-14	0.324586122
**Myd88**	1.628949259	2.07E-23	1.01E-21	3.092876568	**Tmem26**	-1.623702022	1.34E-11	8.66E-11	0.324501708
**Pou1f1**	1.628387949	0.0016858	0.00425672	3.091673456	**F8a**	-1.623790809	8.31E-11	4.87E-10	0.324481738
**Lilrb4a**	1.626849891	0.0012808	0.00328419	3.088379178	**Cdkl2**	-1.625346297	1.99E-08	8.68E-08	0.324132076
**Cask**	1.625232633	2.11E-21	6.78E-20	3.08491905	**Wwp1**	-1.626542691	2.22E-17	3.35E-16	0.323863392
**Carmil1**	1.62443935	2.30E-10	1.27E-09	3.083223237	**Abhd15**	-1.627292321	6.84E-06	2.26E-05	0.323695156
**Macroh2a1**	1.622820925	8.17E-23	3.49E-21	3.079766397	**Pcbp3**	-1.628205652	3.98E-07	1.49E-06	0.323490298
**Mctp2**	1.622425705	2.63E-05	8.14E-05	3.078922822	**Thap3**	-1.629132879	1.50E-08	6.63E-08	0.323282456
**Camk2b**	1.622351713	0.0039664	0.00955922	3.078764918	**Mis18a**	-1.630384547	1.19E-10	6.82E-10	0.323002101
**Dnaja1**	1.61946589	9.13E-21	2.57E-19	3.072612619	**Stimate**	-1.630796848	3.76E-10	2.03E-09	0.322909805
**Dnajb3**	1.619452196	0.0001271	0.00036481	3.072583453	**C1galt1**	-1.630829049	2.95E-19	6.19E-18	0.322902597
**Ggct**	1.61607258	3.31E-12	2.29E-11	3.065394132	**Polg2**	-1.631451294	0.0002878	0.000792728	0.322763357
**Hopx**	1.610866185	1.19E-15	1.35E-14	3.054351681	**H2az2**	-1.631742013	1.39E-10	7.89E-10	0.322698323
**Dst**	1.610404704	1.18E-13	9.95E-13	3.053374828	**Cryzl2**	-1.632093152	3.87E-13	3.05E-12	0.322619791
**Icos**	1.610311972	1.61E-05	5.08E-05	3.053178573	**Mageh1**	-1.634848984	0.001492083	0.003793273	0.322004112
**Scd4**	1.609985432	1.47E-05	4.68E-05	3.052487594	**Fes**	-1.635379197	9.26E-21	2.60E-19	0.321885792
**Olfr1307**	1.609943714	1.45E-09	7.22E-09	3.052399328	**Arhgap45**	-1.636135996	2.03E-19	4.37E-18	0.321716984
**Sh3pxd2b**	1.607599508	3.79E-20	9.57E-19	3.047443574	**Hmbs**	-1.636692428	1.11E-15	1.26E-14	0.321592925
**Zfp281**	1.607448146	4.40E-16	5.30E-15	3.047123865	**Abcb4**	-1.637097001	0.0028806	0.007067244	0.321502754
**Eif6**	1.604179521	1.18E-19	2.67E-18	3.040227999	**Lta4h**	-1.63760093	1.82E-19	3.98E-18	0.321390473
**Apbb1ip**	1.603600856	2.14E-22	8.28E-21	3.039008808	**Rgs10**	-1.638034652	4.70E-18	7.84E-17	0.321293867
**Vmn2r108**	1.601843738	1.35E-05	4.32E-05	3.035309725	**Scml4**	-1.638635923	0.0004762	0.001277708	0.32115999
**Tank**	1.601380795	1.55E-21	5.10E-20	3.034335889	**Timm21**	-1.63864018	4.13E-05	0.000124727	0.321159042
**Tspan33**	1.601201487	0.0004288	0.00115645	3.033958785	**Galnt12**	-1.639219721	0.0028502	0.006996653	0.321030056
**Serping1**	1.599844763	3.70E-08	1.56E-07	3.031106962	**Prxl2a**	-1.639994604	1.47E-15	1.64E-14	0.320857674
**Trim14**	1.599724606	4.22E-20	1.05E-18	3.030854522	**Card11**	-1.641410231	6.48E-10	3.39E-09	0.320542991
**Zfp719**	1.598869348	3.63E-16	4.42E-15	3.029058305	**Mctp1**	-1.64396391	1.86E-16	2.39E-15	0.319976108
**Olfr1417**	1.598710346	0.0033824	0.00822472	3.028724484	**Dph7**	-1.649250895	4.47E-07	1.67E-06	0.31880565
**Spdya**	1.598693837	3.66E-07	1.38E-06	3.028689827	**Lmna**	-1.649499104	4.27E-20	1.06E-18	0.318750806
**Chic2**	1.598626581	1.62E-19	3.56E-18	3.028548639	**Hectd3**	-1.649680639	1.97E-18	3.51E-17	0.3187107
**Med13**	1.59738541	3.43E-13	2.72E-12	3.025944255	**Trim65**	-1.649912933	1.37E-09	6.85E-09	0.318659387
**Vopp1**	1.597177253	2.19E-21	6.99E-20	3.025507693	**Tanc2**	-1.650850414	1.82E-10	1.02E-09	0.318452386
**Bcorl1**	1.596206102	5.75E-14	5.05E-13	3.023471756	**Igip**	-1.652504421	7.13E-08	2.91E-07	0.318087499
**Alcam**	1.595825378	8.05E-23	3.46E-21	3.022673974	**Pkd1**	-1.653802196	3.92E-09	1.86E-08	0.317801492
**Plcl1**	1.59288052	0.0012430	0.00319332	3.016510325	**Uchl3**	-1.65506584	4.20E-12	2.87E-11	0.317523254
**Olfr204**	1.591943993	4.75E-06	1.60E-05	3.014552788	**Hes6**	-1.65546972	6.27E-13	4.81E-12	0.317434377
**Vmn2r11**	1.591718355	6.90E-15	6.98E-14	3.014081349	**Mars2**	-1.655907805	1.54E-10	8.71E-10	0.317338
**Tbrg1**	1.58713292	1.56E-20	4.25E-19	3.004516657	**Pou6f1**	-1.656220748	4.45E-05	0.000134109	0.317269172
**Ehd2**	1.586143565	3.31E-09	1.58E-08	3.00245696	**Tarbp1**	-1.657964769	2.69E-10	1.48E-09	0.316885869
**Vegfc**	1.585035743	5.94E-07	2.18E-06	3.000152307	**Hexdc**	-1.658047487	1.33E-06	4.71E-06	0.3168677
**Tapbpl**	1.58496406	1.11E-21	3.71E-20	3.000003242	**Phf7**	-1.658199198	2.03E-08	8.82E-08	0.316834381
**Gm14444**	-1.586120718	0.0010681	0.00276299	0.333065836	**Nr2f6**	-1.65868301	2.64E-14	2.45E-13	0.316728148
**Lancl1**	-1.589515493	9.44E-15	9.38E-14	0.332283027	**Rnh1**	-1.659807456	3.77E-22	1.38E-20	0.316481384
**Arhgap4**	-1.589917392	2.98E-15	3.19E-14	0.332190474	**Casp6**	-1.659983155	7.05E-12	4.68E-11	0.316442843
**Acad11**	-1.590725717	1.13E-12	8.36E-12	0.332004404	**Atraid**	-1.660613958	1.75E-17	2.69E-16	0.316304512
**Ethe1**	-1.591563954	1.42E-12	1.03E-11	0.331811558	**Fktn**	-1.66128595	2.03E-12	1.45E-11	0.316157215
**Gstp1**	-1.591680359	3.67E-14	3.33E-13	0.331784787	**Nat10**	-1.662009405	9.52E-16	1.09E-14	0.315998715
**Osgin1**	-1.592770128	8.49E-11	4.97E-10	0.331534261	**Fabp4**	-1.662601244	1.58E-20	4.31E-19	0.315869108
**Irf2bpl**	-1.593124691	4.94E-19	9.86E-18	0.331452792	**Swsap1**	-1.662697047	7.49E-11	4.41E-10	0.315848134
**H2-DMa**	-1.594047366	6.42E-16	7.59E-15	0.331240879	**Jun**	-1.663758821	3.43E-21	1.05E-19	0.315615766
**Tsacc**	-1.594509968	0.0005085	0.00136033	0.331134683	**Ccdc57**	-1.663773433	8.48E-07	3.07E-06	0.315612569
**Tmem126b**	-1.595358941	1.21E-14	1.19E-13	0.33093988	**Snx7**	-1.665515361	2.80E-15	3.02E-14	0.315231725
**Camk2g**	-1.595585539	5.54E-15	5.69E-14	0.330887905	**Gng2**	-1.665996649	2.68E-20	6.91E-19	0.31512658
**Ppa2**	-1.595630569	9.34E-12	6.12E-11	0.330877577	**Mfsd12**	-1.666907371	1.26E-21	4.21E-20	0.314927714
**Gorasp1**	-1.597315041	2.54E-15	2.75E-14	0.330491474	**Ccdc28b**	-1.667817249	8.84E-07	3.20E-06	0.314729159
**Oxnad1**	-1.59998078	1.30E-09	6.52E-09	0.329881373	**AW146154**	-1.669339604	2.02E-07	7.82E-07	0.314397227
**Gpr19**	-1.602242708	3.87E-07	1.45E-06	0.329364573	**Bag2**	-1.669547461	0.0004451	0.001198721	0.314351933
**Aph1b**	-1.602619868	2.29E-12	1.62E-11	0.32927848	**1110032A03Rik**	-1.669656566	7.18E-11	4.23E-10	0.314328161
**Nmrk1**	-1.605421573	5.65E-09	2.63E-08	0.328639644	**Slc26a11**	-1.670966291	1.46E-09	7.29E-09	0.314042933
**Kantr**	-1.60550545	5.20E-08	2.16E-07	0.328620537	**Rcor3**	-1.671006717	9.67E-10	4.94E-09	0.314034133
**Abhd3**	-1.605765629	8.34E-05	0.00024438	0.328561278	**Stra6l**	-1.671248714	1.96E-20	5.21E-19	0.313981462
**Gapt**	-1.606195535	3.73E-11	2.27E-10	0.328463386	**Hsd17b10**	-1.672103367	3.73E-18	6.40E-17	0.313795514
**Emilin1**	-1.607271806	9.52E-16	1.09E-14	0.328218439	**Ap1s2**	-1.673446118	5.91E-19	1.16E-17	0.313503593

Gene	log2 (FC)	P-value	FDR	FC	Gene	log2 (FC)	P-value	FDR	FC
**H6pd**	-1.673599158	1.03E-20	2.88E-19	0.313470339	**B4galnt1**	-1.738034769	1.32E-20	3.64E-19	0.299777754
**Igf1**	-1.673621348	2.81E-23	1.31E-21	0.313465517	**Tbc1d8b**	-1.739232248	2.57E-13	2.08E-12	0.299529033
**Anxa9**	-1.67555227	1.56E-08	6.91E-08	0.313046251	**Fggy**	-1.739361018	1.46E-11	9.38E-11	0.299502299
**Dhodh**	-1.675883611	3.92E-11	2.38E-10	0.312974363	**Fam98c**	-1.740529869	4.52E-11	2.72E-10	0.299259745
**Sulf2**	-1.67677283	3.26E-19	6.77E-18	0.312781517	**Crocc**	-1.740873011	3.63E-08	1.53E-07	0.299188575
**Cd320**	-1.678123143	5.86E-07	2.16E-06	0.312488902	**Zfp108**	-1.741271686	0.0004674	0.001255715	0.299105908
**Car5b**	-1.678569341	1.15E-09	5.83E-09	0.31239227	**Dmxl2**	-1.741668674	8.64E-15	8.64E-14	0.299023614
**Ivns1abp**	-1.678879987	5.79E-21	1.70E-19	0.312325012	**Vcpkmt**	-1.741728669	3.01E-11	1.85E-10	0.29901118
**Itpr3**	-1.679901818	9.04E-10	4.64E-09	0.312103877	**Abhd11**	-1.743908674	1.21E-11	7.86E-11	0.298559696
**Dennd4c**	-1.680421502	5.00E-18	8.28E-17	0.311991472	**Maml3**	-1.745150742	3.00E-06	1.03E-05	0.298302766
**Pxylp1**	-1.680896958	2.41E-08	1.04E-07	0.311888668	**Zc3h8**	-1.745793761	1.18E-09	5.93E-09	0.29816984
**Cdpf1**	-1.681711306	9.54E-14	8.12E-13	0.311712668	**Vmn2r13**	-1.745797133	0.0027275	0.006712886	0.298169143
**Arpin**	-1.682033738	2.08E-10	1.16E-09	0.311643011	**Ebpl**	-1.745825197	1.00E-09	5.10E-09	0.298163343
**Mcm2**	-1.682214954	1.46E-14	1.41E-13	0.311603868	**Lmbr1**	-1.747411666	5.98E-09	2.77E-08	0.297835646
**Ncs1**	-1.683194929	4.66E-09	2.19E-08	0.311392278	**Gm14326**	-1.747959279	2.69E-13	2.17E-12	0.297722616
**Lrba**	-1.683490986	5.33E-12	3.60E-11	0.311328383	**Ddhd2**	-1.749571443	1.09E-16	1.45E-15	0.297390106
**Tesmin**	-1.683567484	0.0031637	0.00772583	0.311311875	**Iqgap3**	-1.74970867	0.0030628	0.007490483	0.29736182
**Creb3l3**	-1.683805389	0.0017340	0.00437494	0.311260543	**Noa1**	-1.749739035	1.37E-12	1.00E-11	0.297355562
**Rfx3**	-1.684398351	0.0001161	0.00033489	0.311132638	**Lrrc51**	-1.750894057	0.0024809	0.006139466	0.297117594
**Cracr2a**	-1.684471655	3.00E-11	1.85E-10	0.31111683	**Recql**	-1.750954146	5.14E-14	4.56E-13	0.297105219
**Slc22a4**	-1.68663167	2.42E-15	2.62E-14	0.310651372	**Uvssa**	-1.751451042	0.0001810	0.000510797	0.297002907
**Dut**	-1.688178123	3.62E-08	1.53E-07	0.310318557	**Cdc14b**	-1.753441625	2.77E-12	1.94E-11	0.296593395
**Poli**	-1.688713361	4.31E-09	2.03E-08	0.31020345	**2700049A03Rik**	-1.754091384	8.22E-10	4.24E-09	0.296459846
**Ntpcr**	-1.690091476	1.74E-12	1.26E-11	0.309907274	**Zfp78**	-1.755554427	0.0003597	0.000978696	0.296159357
**Pstpip1**	-1.690388084	6.86E-18	1.11E-16	0.309843566	**Ick**	-1.756511105	3.64E-10	1.97E-09	0.295963033
**Asap2**	-1.690660956	6.58E-11	3.89E-10	0.309784968	**Srd5a1**	-1.756670317	1.82E-05	5.72E-05	0.295930373
**Slc22a18**	-1.693432857	8.69E-07	3.14E-06	0.309190338	**Elmod3**	-1.757395105	2.53E-10	1.39E-09	0.29578174
**Cdc42ep3**	-1.695297599	5.85E-10	3.09E-09	0.308790955	**Cbx7**	-1.759501149	1.03E-06	3.70E-06	0.295350273
**Gstm1**	-1.696548536	6.27E-22	2.20E-20	0.308523324	**Wbp1**	-1.759758395	2.05E-11	1.29E-10	0.295297614
**Lage3**	-1.697298171	1.51E-12	1.10E-11	0.308363055	**Chtf18**	-1.759785592	1.57E-07	6.15E-07	0.295292047
**Tmem104**	-1.697704924	2.05E-19	4.41E-18	0.308276127	**Crip1**	-1.760515574	7.42E-17	1.02E-15	0.295142672
**Mknk1**	-1.701561386	4.46E-18	7.51E-17	0.307453176	**Pfkfb2**	-1.762198714	9.55E-09	4.33E-08	0.29479854
**Gm14548**	-1.70212955	8.89E-21	2.51E-19	0.307332118	**Lrrc27**	-1.762464712	1.62E-14	1.55E-13	0.294744192
**Atpaf1**	-1.702557504	9.61E-13	7.17E-12	0.307240966	**Tmem38a**	-1.762480219	0.0002399	0.000667877	0.294741024
**Cnst**	-1.703128144	3.22E-13	2.57E-12	0.307119465	**Nudt8**	-1.763192622	6.91E-08	2.83E-07	0.294595516
**Platr25**	-1.704062084	9.69E-09	4.39E-08	0.306920713	**Fhod1**	-1.763733078	8.14E-14	7.00E-13	0.294485177
**Phpt1**	-1.704175219	3.59E-08	1.52E-07	0.306896645	**Dsn1**	-1.76416931	7.68E-07	2.79E-06	0.294396146
**Cdc7**	-1.705171441	9.26E-08	3.74E-07	0.306684798	**Calcoco1**	-1.765174309	2.03E-14	1.92E-13	0.294191137
**Dhrs13**	-1.705602515	0.0006161	0.00163121	0.306593174	**Mcee**	-1.768720182	1.24E-13	1.04E-12	0.293468959
**Cradd**	-1.706169777	1.01E-09	5.15E-09	0.306472647	**Pigb**	-1.770379501	4.96E-14	4.41E-13	0.293131619
**Extl2**	-1.706633405	1.49E-14	1.44E-13	0.306374174	**Tex2**	-1.772089259	4.20E-18	7.12E-17	0.29278443
**Depdc7**	-1.707492682	6.35E-08	2.61E-07	0.30619175	**Dock7**	-1.772921737	1.53E-16	1.99E-15	0.292615534
**Asap1**	-1.709300332	5.48E-21	1.62E-19	0.305808342	**Zfhx3**	-1.773998267	4.62E-11	2.78E-10	0.292397267
**E2f6**	-1.709901832	1.50E-10	8.49E-10	0.305680869	**1190007I07Rik**	-1.7743256	1.87E-09	9.20E-09	0.292330933
**Slc19a1**	-1.709960436	2.46E-11	1.53E-10	0.305668452	**Sh3glb2**	-1.778313078	1.34E-12	9.84E-12	0.291524072
**Pms1**	-1.711270812	1.39E-07	5.48E-07	0.305390944	**Pip4p2**	-1.778396021	4.30E-16	5.19E-15	0.291507312
**2300009A05Rik**	-1.712101905	9.48E-10	4.85E-09	0.305215068	**Rgl2**	-1.779263613	1.40E-17	2.17E-16	0.291332062
**Gm14391**	-1.712961111	4.77E-07	1.77E-06	0.30503335	**Lima1**	-1.779981083	5.92E-20	1.43E-18	0.291187215
**Rasgrp4**	-1.713060554	3.47E-12	2.40E-11	0.305012325	**Mrpl42**	-1.780594548	1.47E-12	1.07E-11	0.291063422
**4930579G24Rik**	-1.713524944	8.45E-07	3.06E-06	0.30491416	**Amz1**	-1.780743727	3.33E-17	4.86E-16	0.291033327
**Fgfr1**	-1.714548914	9.69E-18	1.54E-16	0.304697821	**Tmem141**	-1.784637572	1.46E-08	6.46E-08	0.290248885
**Cdiptos**	-1.715100467	0.0002048	0.00057457	0.304581355	**Fam89b**	-1.785026431	2.41E-21	7.61E-20	0.290170662
**Bicdl1**	-1.715266619	9.73E-07	3.50E-06	0.304546279	**Tmc6**	-1.785370068	2.84E-15	3.05E-14	0.290101555
**Adam8**	-1.715825952	1.88E-23	9.26E-22	0.304428229	**Accs**	-1.785951987	3.72E-09	1.77E-08	0.289984564
**Tmem223**	-1.715855642	4.51E-13	3.54E-12	0.304421964	**Sh3yl1**	-1.786057864	2.19E-05	6.83E-05	0.289963283
**Soat1**	-1.717507796	8.25E-24	4.50E-22	0.304073544	**Man1c1**	-1.786280588	9.97E-16	1.14E-14	0.289918522
**Apln**	-1.720230989	0.0007209	0.00189666	0.303500124	**Rassf8**	-1.78701784	4.57E-18	7.65E-17	0.289770405
**Tecpr1**	-1.720283178	2.98E-20	7.62E-19	0.303489145	**Tmem109**	-1.787091719	7.25E-21	2.09E-19	0.289755566
**Fam168a**	-1.720701816	2.60E-18	4.55E-17	0.303401092	**Ehbp1**	-1.787882261	8.81E-09	4.02E-08	0.289596835
**Entpd4**	-1.722332924	1.08E-16	1.43E-15	0.303058261	**Zfp442**	-1.788481101	0.0003172	0.00086879	0.289476653
**Lilra6**	-1.723557416	9.73E-17	1.31E-15	0.302801149	**Armc9**	-1.788814535	1.53E-05	4.86E-05	0.289409757
**Cebpzos**	-1.723654926	2.70E-11	1.67E-10	0.302780684	**Auh**	-1.789055247	5.64E-13	4.35E-12	0.289361473
**Lrrn4**	-1.724234982	0.0002771	0.00076512	0.302658971	**Cyb5a**	-1.7899229	1.87E-20	5.01E-19	0.2891875
**P3h3**	-1.725820965	1.05E-09	5.33E-09	0.302326435	**Mlh3**	-1.795054969	7.16E-12	4.75E-11	0.288160607
**Capn15**	-1.727579211	2.74E-11	1.69E-10	0.301958207	**Bzw2**	-1.795433732	7.68E-11	4.51E-10	0.288084964
**C2cd2l**	-1.727754305	8.63E-17	1.17E-15	0.301921562	**Klf10**	-1.795640525	6.27E-17	8.70E-16	0.288043673
**Tube1**	-1.731794712	0.0003195	0.00087459	0.301077184	**4930438A08Rik**	-1.796190785	0.000166868	0.000472659	0.287933831
**Fuz**	-1.731815036	3.26E-09	1.56E-08	0.301072942	**Myrf**	-1.796929602	6.50E-08	2.67E-07	0.287786415
**Inpp5f**	-1.732979919	6.30E-12	4.21E-11	0.300829944	**Arl15**	-1.797017223	3.95E-09	1.87E-08	0.287768937
**Traip**	-1.733287426	3.13E-05	9.58E-05	0.300765829	**Ifitm6**	-1.798222144	1.12E-14	1.10E-13	0.287528697
**Ech1**	-1.733339393	1.70E-18	3.09E-17	0.300754996	**Suclg2**	-1.798938748	5.09E-15	5.26E-14	0.287385913
**Zbtb38**	-1.733766241	1.87E-15	2.06E-14	0.300666025	**Lfng**	-1.799956417	2.33E-16	2.94E-15	0.287183264
**Coro1c**	-1.734383431	6.33E-25	4.53E-23	0.300537427	**Syngr1**	-1.800461578	7.98E-21	2.27E-19	0.287082724
**Trub1**	-1.735002242	8.01E-10	4.15E-09	0.300408545	**Ednrb**	-1.800623437	2.99E-19	6.25E-18	0.287050518
**Kif22**	-1.735257028	3.64E-06	1.24E-05	0.300355497	**Hs6st1**	-1.80126925	5.04E-16	6.04E-15	0.28692205
**Gata6**	-1.736277795	4.37E-07	1.63E-06	0.300143058	**Ptpdc1**	-1.802565978	2.47E-06	8.54E-06	0.286664274
**Clec4n**	-1.736330207	6.04E-18	9.85E-17	0.300132154	**Tmem9**	-1.803037495	5.11E-13	3.97E-12	0.286570598
**Ophn1**	-1.736422712	8.94E-13	6.70E-12	0.30011291	**Trpv4**	-1.8032148	7.48E-11	4.41E-10	0.286535382
**C1qtnf12**	-1.736550377	5.99E-09	2.78E-08	0.300086354	**Cisd1**	-1.80417287	6.61E-15	6.71E-14	0.286345161
**Def8**	-1.737078364	1.90E-19	4.12E-18	0.299976551	**Lrfn4**	-1.806442256	5.58E-06	1.86E-05	0.285895089
**Rpp40**	-1.737977224	5.40E-10	2.86E-09	0.299789711	**H3c14**	-1.807689525	2.04E-07	7.92E-07	0.285648028

Gene	log2 (FC)	P-value	FDR	FC	Gene	log2 (FC)	P-value	FDR	FC
**Gm14410**	-1.808269843	1.32E-08	5.87E-08	0.28553315	**Wdr91**	-1.883263514	3.20E-22	1.19E-20	0.271069836
**Dock2**	-1.809320911	8.30E-23	3.54E-21	0.285325202	**Cyp2u1**	-1.883659161	0.0018785	0.004724616	0.270995507
**Ccdc34**	-1.809435281	7.93E-09	3.63E-08	0.285302584	**Nhlrc1**	-1.884070333	6.31E-07	2.31E-06	0.270918284
**Ptgr1**	-1.810497546	9.46E-20	2.17E-18	0.285092591	**Eno3**	-1.884712617	5.66E-12	3.81E-11	0.270797699
**Chst14**	-1.810860821	8.92E-16	1.03E-14	0.285020813	**Sord**	-1.884768227	2.48E-17	3.70E-16	0.270787261
**Ccr2**	-1.812261176	1.54E-12	1.12E-11	0.284744291	**Gramd4**	-1.8848835	2.77E-13	2.23E-12	0.270765625
**Fbxo9**	-1.813060233	2.25E-15	2.45E-14	0.284586625	**B4galt4**	-1.886025258	4.14E-06	1.40E-05	0.270551424
**Zfp882**	-1.813336935	3.11E-05	9.55E-05	0.284532048	**1700003F12 Rik**	-1.88625144	0.0001878 42	0.000529301	0.270509011
**Nudt6**	-1.814428009	0.0001575	0.0004474	0.284316945	**Dctpp1**	-1.886390676	5.62E-13	4.34E-12	0.270482906
**Prr11**	-1.816293253	0.0004257	0.00114876	0.283949593	**Zfp810**	-1.888372608	1.33E-08	5.93E-08	0.270111579
**Hdac10**	-1.819664702	3.38E-06	1.15E-05	0.283286803	**Cdca3**	-1.888797587	5.07E-06	1.70E-05	0.270032023
**Ccnb1**	-1.819770699	9.31E-07	3.36E-06	0.28326599	**Galnt4**	-1.889042867	1.70E-16	2.19E-15	0.269986118
**Bbs7**	-1.820186119	1.41E-09	7.04E-09	0.283184436	**Mical1**	-1.89151036	4.40E-14	3.94E-13	0.269524745
**Ankrd10**	-1.822968688	7.80E-12	5.16E-11	0.282638776	**Rarg**	-1.892354341	1.90E-19	4.12E-18	0.269367119
**Nrp1**	-1.823657992	7.65E-21	2.19E-19	0.282503767	**Itgb3bp**	-1.89333127	0.0011042	0.00285359	0.269184777
**Pwwp3a**	-1.823666185	1.55E-12	1.13E-11	0.282502162	**Slc36a1**	-1.896877328	4.21E-18	7.13E-17	0.268523949
**Tmem143**	-1.825726316	1.06E-09	5.40E-09	0.282099044	**Map3k15**	-1.897032605	3.47E-11	2.12E-10	0.26849505
**Zfp760**	-1.825727025	1.04E-09	5.31E-09	0.282098906	**Zbtb20**	-1.897430296	1.34E-10	7.62E-10	0.268421047
**Sclt1**	-1.827315941	0.0002175	0.00060854	0.281788386	**Cerk**	-1.898134173	2.07E-18	3.69E-17	0.268290119
**Dusp18**	-1.82754275	1.10E-10	6.36E-10	0.281744089	**Peg12**	-1.898992619	0.0029090	0.007131302	0.268130526
**Crppa**	-1.828178099	1.02E-05	3.29E-05	0.281620039	**Gpr180**	-1.900204798	3.17E-14	2.91E-13	0.267905332
**Amacr**	-1.828613361	5.86E-09	2.72E-08	0.281535087	**Rasgrf2**	-1.900991814	0.0002196	0.000613824	0.267759225
**Rassf2**	-1.830348906	5.94E-19	1.16E-17	0.281196607	**Zfp12**	-1.90105804	3.73E-06	1.27E-05	0.267746934
**Cysltr1**	-1.830582883	1.10E-13	9.32E-13	0.281151006	**Slc17a5**	-1.901232515	1.40E-18	2.60E-17	0.267714555
**Ivd**	-1.830840491	3.51E-17	5.11E-16	0.281100809	**Rem1**	-1.904729711	0.0009630	0.002502973	0.267066382
**Cdca8**	-1.831838424	7.47E-09	3.43E-08	0.280906434	**Srl**	-1.904759385	0.0031830	0.007769611	0.267060889
**Tctex1d4**	-1.832382672	0.0001591	0.00045153	0.280800484	**Usp2**	-1.905788212	3.49E-09	1.67E-08	0.266870508
**Racgap1**	-1.832953843	1.04E-09	5.29E-09	0.280689336	**Adcy9**	-1.908114941	3.90E-11	2.36E-10	0.266440456
**Mcemp1**	-1.833139879	1.32E-18	2.46E-17	0.280653143	**Prr15**	-1.9096701	0.0016670	0.004212755	0.2661534
**Rb1**	-1.833168324	1.08E-18	2.04E-17	0.28064761	**Camkmt**	-1.910116581	1.26E-08	5.63E-08	0.266071044
**Enc1**	-1.834151727	6.31E-13	4.83E-12	0.280456373	**Ccdc157**	-1.9102075	0.0022534	0.005609628	0.266054277
**Hsd3b7**	-1.836329485	1.86E-17	2.84E-16	0.280033342	**Kif20b**	-1.911047724	7.10E-05	0.000209192	0.265899372
**Epb41**	-1.83642224	1.88E-19	4.09E-18	0.280015338	**Tbc1d31**	-1.911613157	7.81E-15	7.86E-14	0.265795179
**Pink1**	-1.837259416	4.87E-18	8.09E-17	0.279852896	**Sgo2a**	-1.911693004	0.0013561	0.003469147	0.265780469
**Ttc8**	-1.837640772	3.01E-07	1.14E-06	0.279778931	**Gpt**	-1.912791614	3.07E-07	1.17E-06	0.265578155
**Pkib**	-1.83901213	2.15E-14	2.02E-13	0.279513113	**Erp29**	-1.913463843	2.03E-21	6.54E-20	0.265454436
**Rogdi**	-1.83936646	2.45E-08	1.06E-07	0.279444472	**Rfx2**	-1.914567861	8.21E-05	0.000240647	0.265251376
**Rttn**	-1.839515599	1.94E-10	1.09E-09	0.279415586	**Galm**	-1.916525997	3.04E-16	3.75E-15	0.2648916
**Stab1**	-1.839703099	1.50E-18	2.76E-17	0.279379274	**Pira2**	-1.916596741	2.70E-23	1.27E-21	0.264878612
**Spc25**	-1.840479604	3.66E-06	1.24E-05	0.279228943	**Wdr60**	-1.916702787	4.40E-08	1.84E-07	0.264859142
**Klhl36**	-1.8408864	2.11E-11	1.32E-10	0.27915022	**Bivm**	-1.919768297	4.19E-13	3.30E-12	0.264296954
**Ncapd3**	-1.841107923	3.49E-10	1.89E-09	0.279107361	**Csf1r**	-1.920391463	4.61E-24	2.67E-22	0.264182817
**Prkch**	-1.84415193	1.46E-17	2.27E-16	0.27851908	**Tmlhe**	-1.920465087	5.13E-09	2.40E-08	0.264169335
**Xxylt1**	-1.845712532	5.09E-16	6.10E-15	0.278217962	**Gpx1**	-1.920670434	2.36E-22	9.03E-21	0.264131737
**Sall2**	-1.845850937	0.0002085	0.00058429	0.278191272	**Acyp1**	-1.921346924	9.03E-11	5.26E-10	0.264007913
**Grk5**	-1.84628561	2.20E-18	3.91E-17	0.278107468	**Kif26b**	-1.922457882	0.0016894	0.004265479	0.26380469
**Mettl27**	-1.84750266	1.88E-07	7.31E-07	0.277872957	**Pdgfc**	-1.922900067	0.0014737	0.003749711	0.263723846
**Bcl2**	-1.847672326	4.57E-15	4.75E-14	0.27784028	**Zik1**	-1.922967299	0.0003062	0.000840249	0.263711557
**Pop5**	-1.847770471	6.74E-12	4.49E-11	0.277821379	**Cad**	-1.924248042	1.95E-18	3.49E-17	0.263477552
**Tmem150a**	-1.848187019	1.86E-11	1.18E-10	0.277741176	**Mdp1**	-1.924846184	3.88E-14	3.50E-13	0.263368337
**Zfp69**	-1.848640018	2.79E-05	8.61E-05	0.27765398	**Kctd17**	-1.927843561	5.14E-12	3.48E-11	0.262821725
**Itga6**	-1.849454252	5.53E-16	6.61E-15	0.277497321	**Fuom**	-1.928353334	7.07E-13	5.38E-12	0.262728874
**Zfp93**	-1.85075928	1.55E-05	4.93E-05	0.277246417	**Sash3**	-1.930485107	1.73E-20	4.67E-19	0.262340944
**Slc35g1**	-1.851806245	7.28E-05	0.00021441 5	0.277045292	**4930430F08 Rik**	-1.932484414	1.38E-09	6.90E-09	0.26197764
**Tmem238**	-1.852629562	1.85E-06	6.49E-06	0.276887233	**2900026A0 2Rik**	-1.933023417	0.0025430 84	0.006283238	0.261879781
**Clec10a**	-1.855429946	5.10E-14	4.52E-13	0.276350294	**Zbtb14**	-1.936781426	4.85E-17	6.88E-16	0.26119851
**Zfp971**	-1.856437766	3.33E-12	2.31E-11	0.276157312	**Sap25**	-1.937251409	0.0003714	0.001009037	0.261113434
**Nfam1**	-1.8577318	7.48E-20	1.77E-18	0.275909722	**Dnmt1**	-1.938105009	1.12E-21	3.76E-20	0.260958987
**L2hgdh**	-1.859528086	4.90E-07	1.82E-06	0.275566404	**Tk1**	-1.939117085	9.81E-05	0.000285186	0.260775984
**Tonsl**	-1.859629152	1.36E-05	4.35E-05	0.2755471	**Tmem151a**	-1.939184346	0.0011969	0.003080817	0.260763826
**Fbxo10**	-1.862271308	4.79E-11	2.88E-10	0.275042924	**Mgst3**	-1.939552163	8.77E-12	5.77E-11	0.260697352
**Prkra**	-1.866111681	3.45E-13	2.74E-12	0.274311749	**Shc2**	-1.943474495	0.0002906	0.000799866	0.259989543
**Mthfd1**	-1.866528342	1.51E-18	2.78E-17	0.274232537	**Entpd5**	-1.943910498	1.12E-16	1.48E-15	0.259910983
**Fut11**	-1.866530183	5.71E-14	5.01E-13	0.274232187	**Kifc5b**	-1.945299341	0.0008231	0.002155197	0.259660894
**Neo1**	-1.867874758	2.38E-09	1.16E-08	0.273976725	**Rasa3**	-1.945912016	9.75E-22	3.30E-20	0.259550646
**Gm2026**	-1.867875375	0.0014393	0.00366776	0.273976608	**BC052040**	-1.946169486	8.92E-11	5.20E-10	0.25950433
**Tatdn3**	-1.870114744	5.84E-08	2.41E-07	0.273551668	**Lrp8**	-1.947377193	1.02E-09	5.21E-09	0.259287185
**Mzt2**	-1.870514334	2.58E-11	1.60E-10	0.273475911	**Ptpn18**	-1.947545027	1.28E-14	1.24E-13	0.259257022
**Cd300ld**	-1.871666499	1.99E-23	9.70E-22	0.273257595	**Mrpl23**	-1.948090555	0.0006709	0.001770217	0.259159008
**Coasy**	-1.872770345	7.47E-16	8.73E-15	0.273048598	**Ptgfrn**	-1.948371115	3.35E-18	5.80E-17	0.259108614
**Atp6v1g2**	-1.873618455	0.0002677	0.00074097	0.27288813	**Polr3gl**	-1.948395546	3.33E-12	2.30E-11	0.259104227
**Atad2**	-1.873882686	8.71E-15	8.70E-14	0.272838154	**Mvb12b**	-1.949316712	3.03E-16	3.75E-15	0.25893884
**Trim62**	-1.874269219	2.65E-10	1.45E-09	0.272765064	**Zfp61**	-1.949773716	3.10E-09	1.49E-08	0.258856829
**Ubxn11**	-1.875481885	0.0001233 34	0.00035486	0.272535886	**Cipc**	-1.950994888	4.42E-21	1.32E-19	0.258637812
**Fmnl1**	-1.876131373	4.89E-20	1.20E-18	0.272413221	**Mamdc2**	-1.952556684	1.59E-11	1.01E-10	0.258357974
**Cep57l1**	-1.876327275	3.31E-08	1.41E-07	0.272376233	**Als2cl**	-1.95369464	8.94E-11	5.21E-10	0.258154269
**Rab3d**	-1.877271541	4.77E-19	9.58E-18	0.272198016	**Slc1a5**	-1.954674143	2.81E-16	3.50E-15	0.257979057
**Map3k4**	-1.877403562	6.08E-16	7.21E-15	0.272173109	**Tnfsf13**	-1.954714128	4.09E-15	4.27E-14	0.257971907
**Pwwp2b**	-1.87890501	4.60E-19	9.27E-18	0.271889999	**Rida**	-1.955288654	9.87E-10	5.04E-09	0.257869195
**Gtf3a**	-1.880327644	8.85E-17	1.20E-15	0.271622022	**Fbxo21**	-1.955538839	5.14E-12	3.48E-11	0.257824481
**Sgf29**	-1.882646511	2.58E-14	2.40E-13	0.27118579	**Krt14**	-1.957106852	0.0005062 62	0.001355052	0.257544413

Gene	log2 (FC)	P-value	FDR	FC	Gene	log2 (FC)	P-value	FDR	FC
**Smyd4**	-1.957737949	1.10E-09	5.59E-09	0.257431776	**Nim1k**	-2.023364721	0.0020494	0.005128922	0.245983812
**Ankrd44**	-1.957883672	2.04E-15	2.23E-14	0.257405775	**Tjp2**	-2.023485804	5.49E-19	1.09E-17	0.245963168
**2210418O10Rik**	-1.95812981	9.44E-16	1.08E-14	0.257361863	**Zfp763**	-2.023864178	1.21E-07	4.78E-07	0.245898668
**Atp1a3**	-1.95908183	2.82E-23	1.31E-21	0.257192089	**Gm14435**	-2.025699504	0.0002912	0.000801181	0.245586046
**Mettl26**	-1.959448598	9.42E-09	4.28E-08	0.257126712	**Cplane2**	-2.027734101	0.0033135	0.008067264	0.245239946
**Xylt1**	-1.959729366	4.71E-16	5.65E-15	0.257076677	**Mblac1**	-2.028290207	1.78E-11	1.13E-10	0.245145434
**Slc17a9**	-1.961342943	1.57E-09	7.82E-09	0.256789311	**Rab27a**	-2.032436503	1.29E-11	8.31E-11	0.244441899
**Sh3pxd2a**	-1.963514775	4.29E-13	3.38E-12	0.256403032	**St6galnac6**	-2.032770482	1.21E-16	1.60E-15	0.244385318
**Zfp41**	-1.965489621	1.93E-12	1.39E-11	0.256052292	**Knstrn**	-2.034443515	1.48E-06	5.23E-06	0.244102079
**Gltp**	-1.965668435	1.70E-24	1.09E-22	0.256020558	**Gfod1**	-2.035178032	2.08E-11	1.30E-10	0.243977831
**Dhrs7**	-1.966669817	7.30E-19	1.41E-17	0.255842914	**Mtg1**	-2.035756495	1.12E-14	1.10E-13	0.243880025
**Rassf3**	-1.966965849	8.74E-20	2.02E-18	0.255790422	**F13a1**	-2.035761467	2.21E-16	2.80E-15	0.243879185
**March9**	-1.967912634	1.87E-12	1.34E-11	0.255622612	**Gm7694**	-2.036161412	8.24E-05	0.000241619	0.243811586
**Tnrc6b**	-1.969550195	2.72E-12	1.90E-11	0.255332627	**Pnpo**	-2.036261566	4.25E-15	4.43E-14	0.24379466
**Gm4631**	-1.970001801	1.68E-19	3.69E-18	0.255252713	**Agfg2**	-2.037078891	2.01E-14	1.90E-13	0.243656584
**Pmm1**	-1.970402042	3.26E-13	2.60E-12	0.255181909	**Cib2**	-2.038479554	2.43E-05	7.55E-05	0.243420141
**Sox7**	-1.970586696	0.0001111	0.00032110	0.25514925	**Cep250**	-2.040307832	3.41E-11	2.09E-10	0.243111858
**Ccpg1os**	-1.975351319	6.18E-05	0.00018347	0.254307987	**Gas2**	-2.040733365	1.41E-05	4.51E-05	0.243040161
**Stk26**	-1.9784964	8.07E-10	4.17E-09	0.253754199	**Inpp5d**	-2.041355814	1.15E-22	4.71E-21	0.242935324
**Pank2**	-1.97909827	1.46E-06	5.16E-06	0.253648359	**Fan1**	-2.04182707	9.67E-10	4.94E-09	0.242855982
**Gm15448**	-1.979258495	1.81E-12	1.30E-11	0.25362019	**Prxl2c**	-2.041840602	1.79E-17	2.73E-16	0.242853704
**Kyat1**	-1.980588272	4.27E-07	1.60E-06	0.253386528	**Vipr2**	-2.042133984	0.0028601	0.007019313	0.242804323
**Tmem42**	-1.980994397	2.10E-09	1.03E-08	0.253315209	**Nudt12**	-2.042681976	0.0003300	0.000901707	0.242712114
**Eml6**	-1.982127652	3.31E-07	1.25E-06	0.253116305	**Garem1**	-2.045309222	0.0024404	0.006045486	0.242270521
**Cbr3**	-1.983734678	3.28E-12	2.28E-11	0.252834514	**Pld4**	-2.04536657	2.93E-21	9.11E-20	0.242260891
**Cpeb1**	-1.984534433	2.15E-10	1.19E-09	0.252694395	**Traf3ip3**	-2.049894734	0.0001647	0.000466639	0.241501703
**Trim37**	-1.984833372	9.83E-17	1.32E-15	0.252642039	**Per2**	-2.05193215	3.50E-08	1.48E-07	0.241160888
**Slc29a1**	-1.985506542	1.07E-19	2.44E-18	0.252524183	**Mnd1**	-2.053078681	0.0039169	0.009445059	0.24096931
**Limk1**	-1.986054906	1.40E-16	1.83E-15	0.252428217	**Fgr**	-2.053771959	2.24E-20	5.86E-19	0.240853541
**Cntnap1**	-1.986162758	0.0005648	0.00150331	0.252409347	**Gm5141**	-2.054303284	1.14E-06	4.08E-06	0.240764855
**Rapgef6**	-1.986484909	2.06E-20	5.45E-19	0.252352991	**Fv1**	-2.054942348	5.04E-05	0.00015117	0.240658228
**Clec5a**	-1.987879952	1.31E-16	1.73E-15	0.252109091	**Wdr7**	-2.055177629	2.01E-20	5.34E-19	0.240618983
**Acsf2**	-1.988169454	8.46E-13	6.36E-12	0.252058506	**Sema6b**	-2.055730225	4.17E-14	3.75E-13	0.240526837
**Mmp8**	-1.98910302	1.68E-16	2.18E-15	0.251895452	**Pcyox1**	-2.05612017	1.46E-21	4.82E-20	0.240461833
**Cmc2**	-1.98912325	8.44E-10	4.35E-09	0.25189192	**Ankrd26**	-2.056196079	2.57E-08	1.11E-07	0.240449182
**Rwdd2a**	-1.990057807	2.33E-06	8.06E-06	0.251728801	**Bcas3**	-2.056423061	1.15E-16	1.52E-15	0.240411354
**Plcb2**	-1.992534	6.27E-17	8.70E-16	0.251297113	**Sccpdh**	-2.057262928	2.74E-11	1.69E-10	0.240271439
**Cbr1**	-1.993021096	6.65E-15	6.74E-14	0.251212282	**Rfng**	-2.058084334	2.18E-14	2.05E-13	0.240134678
**Anln**	-1.995419999	2.26E-06	7.84E-06	0.250794915	**Zfp970**	-2.058652213	3.78E-10	2.04E-09	0.240040174
**Prox2**	-1.995804825	0.0019845	0.00497519	0.250728026	**Palm**	-2.059062931	2.73E-19	5.76E-18	0.239971847
**Ggact**	-1.997110059	0.0008859	0.00231176	0.250501291	**Ncapd2**	-2.060709065	2.36E-10	1.30E-09	0.239698192
**Dcaf4**	-1.998310286	4.05E-12	2.78E-11	0.250292977	**Mmp19**	-2.061797267	9.92E-17	1.33E-15	0.23951746
**Haus8**	-1.99834701	1.89E-18	3.39E-17	0.250286605	**Bnip3l**	-2.061856114	1.59E-23	7.93E-22	0.23950769
**Pctp**	-1.998956248	1.02E-18	1.93E-17	0.250180934	**Zfp667**	-2.061967162	3.68E-07	1.39E-06	0.239489256
**Rap1gap**	-1.999524126	0.003101364	0.00757882	0.250082476	**Crat**	-2.062374407	2.45E-21	7.75E-20	0.239421662
**Rpusd3**	-2.000061232	2.47E-06	8.56E-06	0.24998939	**Dnajc12**	-2.063048757	8.82E-09	4.02E-08	0.239309777
**Myo18a**	-2.000410629	3.15E-18	5.47E-17	0.249928854	**Cers5**	-2.065832084	2.32E-20	6.03E-19	0.238848532
**Smim24**	-2.001388569	3.72E-09	1.77E-08	0.249759495	**Ndrg3**	-2.066617183	2.79E-18	4.87E-17	0.238718589
**Hscb**	-2.001549311	2.96E-15	3.17E-14	0.249731669	**Tlr13**	-2.066865512	2.66E-23	1.25E-21	0.238677502
**Cnpy4**	-2.003521767	1.50E-14	1.45E-13	0.249390469	**Atrip**	-2.068334197	2.20E-05	6.84E-05	0.238434648
**Sipa1l2**	-2.003643916	4.07E-18	6.94E-17	0.249369354	**Nckipsd**	-2.071771513	1.74E-13	1.44E-12	0.237867238
**Tcea2**	-2.003734678	9.16E-05	0.00026720	0.249353667	**Sac3d1**	-2.072229028	2.13E-13	1.74E-12	0.237791817
**Rangrf**	-2.004196015	1.85E-08	8.10E-08	0.249273942	**Sept9**	-2.072391976	5.16E-23	2.27E-21	0.23776496
**Spata2l**	-2.005765904	1.86E-07	7.23E-07	0.249002839	**Hagh**	-2.073044682	3.14E-16	3.86E-15	0.237657415
**Vegfb**	-2.00644457	4.24E-17	6.07E-16	0.248885732	**Ust**	-2.074117515	3.14E-07	1.19E-06	0.237480751
**Maoa**	-2.0065463	9.07E-15	9.03E-14	0.248868182	**Exosc5**	-2.074911615	3.23E-15	3.44E-14	0.237350071
**Gm5150**	-2.00697452	1.87E-09	9.23E-09	0.248794325	**Scarb1**	-2.079067507	7.51E-20	1.78E-18	0.236667333
**Pask**	-2.007053904	0.0039071	0.00942345	0.248780635	**Ptpn22**	-2.081095759	1.34E-14	1.30E-13	0.236334842
**Cenpa**	-2.007161581	0.0011315	0.00292066	0.248762068	**Dhcr24**	-2.08111728	5.53E-19	1.09E-17	0.236331317
**Meis1**	-2.007996903	0.0036342	0.00880454	0.248618076	**Ttyh3**	-2.082119268	4.19E-20	1.05E-18	0.236167236
**Arfgef3**	-2.008474588	1.57E-08	6.95E-08	0.248535771	**Tdo2**	-2.083645753	0.003852298	0.009300676	0.235917484
**AI467606**	-2.00898881	6.17E-18	1.00E-16	0.2484472	**Ptger4**	-2.08610763	8.36E-18	1.34E-16	0.235515248
**Oxld1**	-2.009405425	1.74E-07	6.78E-07	0.248375465	**Plekhg3**	-2.091884561	2.38E-15	2.59E-14	0.234574068
**Zdhhc9**	-2.01098441	3.93E-19	8.06E-18	0.248103775	**Ndc1**	-2.095514947	1.31E-16	1.73E-15	0.23398453
**Pbld1**	-2.011306639	0.0021955	0.00547488	0.248048366	**Tesk2**	-2.095536396	7.35E-08	3.00E-07	0.233981051
**Hpgds**	-2.011937781	3.51E-18	6.05E-17	0.247939875	**Mbp**	-2.096521112	1.54E-17	2.38E-16	0.233821401
**Sirt3**	-2.012796062	1.93E-10	1.08E-09	0.247792416	**Pcgf2**	-2.097160763	3.47E-07	1.31E-06	0.233717754
**Emc9**	-2.013240411	5.02E-13	3.91E-12	0.247716108	**4930404N11Rik**	-2.097275171	9.08E-10	4.66E-09	0.233699221
**Slc45a4**	-2.013595568	2.99E-19	6.25E-18	0.247655134	**Zfp248**	-2.097398477	0.0001159	0.000334504	0.233679248
**Gspt2**	-2.014072416	1.15E-07	4.57E-07	0.247573291	**Zcchc24**	-2.098104051	3.17E-11	1.94E-10	0.233564991
**Smim14**	-2.014200756	3.64E-19	7.48E-18	0.247551268	**Dna2**	-2.100905524	5.54E-14	4.87E-13	0.233111887
**Mak**	-2.014579556	2.32E-08	1.00E-07	0.247486278	**M1ap**	-2.101452418	1.74E-05	5.50E-05	0.233023536
**Pigyl**	-2.014839721	1.75E-13	1.44E-12	0.247441653	**Slc35c2**	-2.101682417	2.34E-19	4.98E-18	0.232986389
**L1cam**	-2.018828776	1.26E-11	8.14E-11	0.246758421	**Il16**	-2.103611982	9.12E-17	1.23E-15	0.232674985
**Acaa2**	-2.018907521	4.01E-18	6.84E-17	0.246744953	**Ddb2**	-2.104117266	0.0007237	0.001903662	0.232593508
**Cyb5rl**	-2.019083381	8.70E-09	3.97E-08	0.246714877	**Tmem241**	-2.104880071	4.19E-06	1.41E-05	0.23247056
**Abcd4**	-2.020476921	1.31E-15	1.47E-14	0.246476683	**Aif1**	-2.10690288	1.67E-13	1.38E-12	0.23214484
**Psma8**	-2.021291644	1.75E-05	5.51E-05	0.246337531	**Rbl2**	-2.107804093	9.00E-16	1.04E-14	0.231999871
**Cbx3**	-2.021949203	3.72E-16	4.53E-15	0.24622528	**Prkar2a**	-2.109697433	7.28E-19	1.41E-17	0.231695602
**Ube2t**	-2.02212438	0.000779914	0.002047075	0.246195384	**Ano6**	-2.110987245	3.89E-20	9.78E-19	0.231488552
**Kcnk6**	-2.022391163	2.30E-19	4.91E-18	0.246149862	**Zfp273**	-2.111384358	1.89E-07	7.36E-07	0.231424842

Gene	log2 (FC)	P-value	FDR	FC	Gene	log2 (FC)	P-value	FDR	FC
**Nfic**	-2.112065383	5.50E-19	1.09E-17	0.231315624	**Nme3**	-2.221207293	2.60E-11	1.61E-10	0.214461815
**D130040H23Rik**	-2.112144986	5.49E-05	0.000163864	0.231302861	**Abcb6**	-2.221546419	1.32E-15	1.48E-14	0.214411409
**E2f1**	-2.112471449	3.48E-14	3.17E-13	0.231250526	**Gm14409**	-2.22190005	0.0009702	0.002520366	0.214358859
**Tssk6**	-2.114884738	1.74E-05	5.47E-05	0.230864022	**Abcd1**	-2.222614764	3.94E-21	1.19E-19	0.214252692
**Kmt5c**	-2.115944589	2.77E-12	1.94E-11	0.230694483	**Acad10**	-2.223299632	5.63E-11	3.35E-10	0.214151007
**Pqlc3**	-2.116521718	9.03E-23	3.80E-21	0.230602216	**Myo7a**	-2.224167175	6.98E-17	9.61E-16	0.214022269
**Tmem268**	-2.119422486	2.09E-20	5.50E-19	0.230139019	**Lyrm9**	-2.225575349	5.68E-09	2.64E-08	0.21381347
**Dio2**	-2.119509176	2.38E-18	4.20E-17	0.230125191	**Akap17b**	-2.22746523	1.39E-08	6.20E-08	0.213533565
**Zfp13**	-2.121215921	0.0003347	0.00091403	0.229853108	**Sec22c**	-2.229297383	1.25E-18	2.33E-17	0.21326256
**Tada2a**	-2.123387212	2.20E-10	1.22E-09	0.229507434	**Tbxas1**	-2.229574537	3.01E-22	1.12E-20	0.213221595
**Bex1**	-2.123951245	7.84E-14	6.75E-13	0.229417723	**Arhgef10l**	-2.233125361	4.46E-20	1.11E-18	0.21269745
**Scml2**	-2.125008725	0.0010358	0.00268405	0.229249624	**Asrgl1**	-2.235022029	9.31E-08	3.76E-07	0.212418006
**Grhpr**	-2.125086787	3.67E-11	2.24E-10	0.22923722	**Stac2**	-2.235650087	9.10E-20	2.09E-18	0.212325553
**Rpusd2**	-2.126777015	1.88E-10	1.05E-09	0.228968809	**Mospd3**	-2.237658036	1.31E-16	1.73E-15	0.212030243
**Plxnc1**	-2.128985633	4.45E-22	1.61E-20	0.228618549	**Them6**	-2.244930141	2.07E-13	1.69E-12	0.210964164
**Loxl3**	-2.129001896	4.38E-09	2.06E-08	0.228615972	**Parp1**	-2.24494492	3.11E-21	9.64E-20	0.210962003
**Slc25a45**	-2.129609288	4.51E-18	7.57E-17	0.228519742	**Acox3**	-2.246556347	8.99E-21	2.53E-19	0.210726499
**Zfp704**	-2.130144828	3.97E-17	5.73E-16	0.228434929	**Coq10a**	-2.246681063	1.89E-16	2.42E-15	0.210708284
**Tmem41a**	-2.13231768	1.56E-12	1.14E-11	0.228091141	**Retnlg**	-2.251164889	3.64E-05	0.000110732	0.210054429
**Ube2cbp**	-2.132417356	0.0025458	0.00628840	0.228075383	**Slc25a42**	-2.251632713	4.58E-06	1.54E-05	0.209986326
**Gne**	-2.134034612	5.25E-14	4.65E-13	0.227819854	**C5ar2**	-2.25239949	1.40E-07	5.50E-07	0.20987475
**Cep112**	-2.135496887	0.0023229	0.00577146	0.22758906	**Irak1bp1**	-2.253973437	1.31E-08	5.84E-08	0.209645906
**Mast3**	-2.140264561	1.54E-18	2.82E-17	0.226838187	**Edaradd**	-2.255539183	1.80E-05	5.67E-05	0.209418502
**Elmsan1**	-2.140982414	6.45E-17	8.92E-16	0.226725346	**Adgre5**	-2.255837648	4.43E-24	2.58E-22	0.209375182
**Nptxr**	-2.141984694	0.0013425	0.00343520	0.226567888	**Morn1**	-2.256627342	1.57E-05	4.98E-05	0.209260607
**Ppp1r12b**	-2.143002373	5.48E-16	6.55E-15	0.226408123	**Antxr2**	-2.257278392	2.77E-21	8.67E-20	0.209166195
**Cdo1**	-2.144264894	3.67E-06	1.25E-05	0.226210077	**Ccdc125**	-2.257840193	2.65E-17	3.95E-16	0.209084759
**Tspan14**	-2.144743083	1.26E-22	5.11E-21	0.226135111	**Ppm1m**	-2.258898184	1.08E-15	1.23E-14	0.208931484
**Arhgap39**	-2.145660937	4.22E-14	3.79E-13	0.225991288	**Sesn1**	-2.258941831	2.03E-14	1.91E-13	0.208925163
**2610318N02Rik**	-2.147028042	0.000148885	0.000424382	0.225777239	**Eef1aknmt**	-2.259150619	1.79E-11	1.14E-10	0.20889493
**Cd244a**	-2.148490049	1.56E-06	5.49E-06	0.225548555	**Oxct1**	-2.260286537	1.78E-24	1.14E-22	0.208730519
**Fam3c**	-2.148674303	7.78E-20	1.84E-18	0.225519751	**Mansc1**	-2.260667663	6.77E-06	2.24E-05	0.208675385
**Tbc1d2**	-2.150709092	5.67E-20	1.37E-18	0.225201901	**Itga10**	-2.262979992	0.0013976	0.00357022	0.208341191
**Adrb2**	-2.154170957	1.08E-13	9.17E-13	0.224662158	**Arhgap15**	-2.263199046	9.43E-09	4.28E-08	0.20830956
**Tnfsfm13**	-2.156898824	5.12E-18	8.46E-17	0.224237765	**Cacnb1**	-2.267143528	9.28E-10	4.76E-09	0.207740797
**Tns2**	-2.157173364	0.0003573	0.00097274	0.224195098	**Gas6**	-2.267927363	3.95E-16	4.79E-15	0.207627959
**Pecr**	-2.157391346	9.27E-10	4.76E-09	0.224161226	**Cxcr3**	-2.268583183	1.83E-05	5.75E-05	0.207533597
**Gm2004**	-2.160770824	0.0003944	0.00106893	0.223636748	**Msrb1**	-2.270219671	1.31E-23	6.77E-22	0.20729832
**Susd1**	-2.162179466	8.59E-12	5.66E-11	0.223418496	**Lipt2**	-2.272418378	1.31E-08	5.83E-08	0.206982632
**Cenpv**	-2.16249396	4.27E-06	1.44E-05	0.223369799	**Enpp1**	-2.273086299	1.36E-11	8.73E-11	0.206886828
**Snx30**	-2.163866433	2.88E-20	7.38E-19	0.223157402	**Myoz1**	-2.275413172	1.87E-05	5.88E-05	0.206553416
**Man2a2**	-2.164467131	3.68E-17	5.34E-16	0.223064505	**Insr**	-2.275794597	3.98E-17	5.74E-16	0.206498814
**Gnpda1**	-2.167192928	2.80E-24	1.68E-22	0.22264345	**Hip1**	-2.277078008	1.50E-19	3.32E-18	0.206315196
**Pms2**	-2.169082218	0.0003915	0.00106131	0.222352076	**Pcdhga12**	-2.278795933	8.69E-05	0.000254118	0.206069667
**Cacnb4**	-2.169417866	9.82E-07	3.53E-06	0.222300351	**Tmem158**	-2.283082501	1.81E-07	7.04E-07	0.205458297
**Ift140**	-2.172388419	3.16E-14	2.90E-13	0.221843099	**Cenpi**	-2.283449134	0.0004676	0.001256078	0.205406091
**Caprin2**	-2.173818704	2.14E-06	7.45E-06	0.221623273	**Gcdh**	-2.284334544	5.83E-15	5.96E-14	0.205280068
**Ptgr2**	-2.175214418	8.15E-20	1.91E-18	0.221408971	**Xrcc6**	-2.284505273	1.32E-15	1.48E-14	0.205255776
**Inka2**	-2.17536345	0.000234454	0.000653847	0.2213861	**Mknk2**	-2.284574793	9.03E-24	4.86E-22	0.205245886
**Cep97**	-2.175648665	5.05E-08	2.10E-07	0.221342337	**Trp53inp1**	-2.287493881	1.13E-05	3.64E-05	0.20483102
**Tcp11l2**	-2.177536574	1.24E-07	4.92E-07	0.221052879	**Megf9**	-2.288010256	2.02E-11	1.27E-10	0.204757719
**Pygl**	-2.177599656	1.42E-21	4.69E-20	0.221043213	**Scfd2**	-2.288120475	2.40E-09	1.17E-08	0.204742076
**Gcat**	-2.177784548	8.70E-12	5.73E-11	0.221014887	**Mnt**	-2.2895896	8.35E-20	1.95E-18	0.20453369
**Uck2**	-2.180671914	6.01E-17	8.36E-16	0.220572997	**Brsk1**	-2.290056255	0.002032929	0.005091245	0.204467542
**Rnasel**	-2.181278273	9.00E-19	1.72E-17	0.22048031	**Arhgef6**	-2.290195127	3.08E-15	3.29E-14	0.204447861
**Mbnl1**	-2.183232675	1.56E-21	5.13E-20	0.22018183	**Poglut2**	-2.290669507	2.87E-12	2.01E-11	0.204380646
**Rtl8b**	-2.185702881	3.00E-14	2.76E-13	0.219805154	**Pde2a**	-2.291650275	0.003275854	0.007981833	0.204241752
**Ahrr**	-2.185708188	6.11E-11	3.63E-10	0.219804345	**Abca5**	-2.291990842	1.28E-06	4.56E-06	0.204193544
**Syp**	-2.188382384	2.76E-07	1.05E-06	0.219397291	**Bfsp1**	-2.292566958	9.41E-05	0.00027405	0.204112019
**Calhm2**	-2.1905959	4.08E-14	3.68E-13	0.219060929	**Ppp1r3e**	-2.293446947	1.08E-09	5.48E-09	0.203987556
**Wdr66**	-2.190948862	0.0037454	0.00905458	0.219007342	**Zfp398**	-2.294252943	5.55E-12	3.74E-11	0.203873626
**Srpk2**	-2.192130176	1.50E-15	1.67E-14	0.218828086	**Cars2**	-2.294871369	1.12E-07	4.48E-07	0.203786252
**Ttc28**	-2.192377638	3.90E-08	1.64E-07	0.218790555	**Ptprs**	-2.295227061	2.92E-15	3.13E-14	0.203736015
**Zfp931**	-2.192729084	1.04E-09	5.28E-09	0.218737263	**Rassf5**	-2.295658887	2.77E-17	4.11E-16	0.203675042
**Kdsr**	-2.194015556	2.47E-14	2.30E-13	0.218542299	**Dock5**	-2.297997895	4.41E-18	7.43E-17	0.203345096
**Mri1**	-2.194143722	2.14E-10	1.19E-09	0.218522885	**Fam169a**	-2.300136183	0.0001813	0.000511862	0.203043932
**Hibch**	-2.194607755	3.18E-12	2.21E-11	0.21845261	**Plxna2**	-2.301288781	1.15E-09	5.81E-09	0.202881781
**Haus5**	-2.199410932	9.07E-11	5.28E-10	0.217726523	**Bbs12**	-2.302800736	3.78E-08	1.59E-07	0.202669271
**Zfp354c**	-2.199574812	0.0036894	0.00892737	0.217701792	**Slc16a13**	-2.30352684	6.17E-14	5.39E-13	0.202567293
**Cep131**	-2.200992397	6.68E-07	2.44E-06	0.217487984	**Gpd1l**	-2.303762601	1.63E-18	2.98E-17	0.202534193
**Cfap298**	-2.203329427	2.64E-10	1.45E-09	0.217135959	**Tmem254b**	-2.304183275	4.87E-06	1.63E-05	0.202475145
**Prkdc**	-2.208887522	6.62E-14	5.77E-13	0.216301036	**Crb2**	-2.305113556	0.0005929	0.001574216	0.202344627
**Trim16**	-2.209930523	1.71E-10	9.61E-10	0.216144717	**Mrgpre**	-2.308582086	5.46E-12	3.68E-11	0.201858734
**Nqo2**	-2.212250768	4.70E-19	9.48E-18	0.215797377	**Ptges3l**	-2.311320447	6.64E-05	0.000196419	0.201475952
**Adk**	-2.212717976	1.16E-17	1.82E-16	0.215727503	**Apbb1**	-2.312205126	4.82E-05	0.00014467	0.201352442
**Chst12**	-2.212986349	4.31E-20	1.07E-18	0.215687377	**Klhl42**	-2.312791373	8.09E-10	4.18E-09	0.201270638
**Ckb**	-2.213879538	1.36E-17	2.12E-16	0.215553884	**Pole2**	-2.313225694	1.17E-09	5.92E-09	0.201210055
**Zfp493**	-2.216413189	7.23E-07	2.64E-06	0.215175662	**B4gat1**	-2.313259423	5.63E-17	7.88E-16	0.201205351
**Kbtbd3**	-2.216623842	3.49E-08	1.48E-07	0.215144246	**Btbd6**	-2.313413297	2.36E-09	1.15E-08	0.201183892
**Nbeal2**	-2.217625985	2.89E-16	3.58E-15	0.214994851	**Fam187b**	-2.316937221	0.0020372	0.005101234	0.20069308
**Rps6ka5**	-2.220524078	3.61E-07	1.36E-06	0.214563402	**Nav1**	-2.318219227	5.64E-19	1.11E-17	0.20051482

Gene	log2 (FC)	P-value	FDR	FC	Gene	log2 (FC)	P-value	FDR	FC
**Per3**	-2.322021288	5.83E-05	0.00017356	0.199987081	**Noxo1**	-2.446803335	0.0001603	0.000454871	0.183416669
**Pde3b**	-2.324209264	2.93E-17	4.33E-16	0.199684013	**Tbc1d32**	-2.448778782	5.49E-12	3.70E-11	0.183165693
**Lamtor4**	-2.326713302	2.26E-19	4.83E-18	0.199337728	**Tfap4**	-2.449615104	1.37E-08	6.11E-08	0.183059544
**Mis18bp1**	-2.32731474	1.95E-07	7.59E-07	0.199254645	**Rtl8a**	-2.450804738	1.39E-13	1.16E-12	0.182908657
**Ndufaf7**	-2.329585291	6.27E-14	5.48E-13	0.198941299	**Cd28**	-2.451892644	2.32E-08	1.00E-07	0.182770781
**Map4k1**	-2.332440264	2.31E-14	2.16E-13	0.198548	**Entpd6**	-2.452994387	9.17E-14	7.85E-13	0.182631258
**Exog**	-2.334039304	0.0004017	0.00108684	0.198328058	**Adamtsl4**	-2.453716208	1.42E-09	7.10E-09	0.182539905
**Krt7**	-2.33435952	0.0006320	0.00167182	0.198284042	**Rgs2**	-2.455079386	8.24E-20	1.93E-18	0.182367508
**Pigz**	-2.335165222	3.59E-09	1.71E-08	0.198173337	**Plpp7**	-2.455359816	3.27E-06	1.12E-05	0.182332063
**Wdpcp**	-2.335634187	6.66E-09	3.07E-08	0.198108929	**Tnfsf12**	-2.456886569	5.54E-22	1.97E-20	0.182139209
**Zfp808**	-2.335887128	8.03E-06	2.63E-05	0.198074199	**Ramp1**	-2.460986246	1.49E-08	6.59E-08	0.181622363
**Pcx**	-2.336413421	3.12E-11	1.92E-10	0.198001955	**Prkar1b**	-2.462472151	1.33E-09	6.65E-09	0.181435397
**Lhpp**	-2.337259279	1.49E-13	1.24E-12	0.1978859	**Cep128**	-2.463189172	4.08E-11	2.47E-10	0.181345246
**Dagla**	-2.339265761	4.00E-09	1.89E-08	0.197610874	**Vamp1**	-2.464636655	2.52E-12	1.77E-11	0.18116339
**5730409E04Rik**	-2.340375614	8.95E-15	8.92E-14	0.197458912	**Pter**	-2.46478284	1.44E-10	8.17E-10	0.181145034
**Gng7**	-2.3462165	0.0041365	0.00993921	0.196661097	**Angptl6**	-2.465708121	2.16E-12	1.54E-11	0.181028893
**Gfra2**	-2.346653112	0.0013420	0.00343433	0.196601589	**Cryl1**	-2.466636241	5.56E-15	5.71E-14	0.18091247
**Kcnab2**	-2.347192719	2.53E-23	1.19E-21	0.196528069	**Rragb**	-2.467790693	0.0008920	0.002326326	0.180767761
**Alg6**	-2.353252507	2.69E-13	2.17E-12	0.195704318	**Dusp9**	-2.467881992	5.27E-07	1.95E-06	0.180756322
**Fam120c**	-2.35799996	1.01E-06	3.63E-06	0.195061375	**AI661453**	-2.474033542	0.0040777	0.009808823	0.17998723
**Nudt7**	-2.358034418	1.96E-11	1.23E-10	0.195056717	**Arhgef4**	-2.475823203	7.55E-07	2.75E-06	0.179764095
**Elovl5**	-2.358527415	6.59E-22	2.30E-20	0.194990073	**Tmem14a**	-2.476962993	1.30E-05	4.15E-05	0.17962213
**Agl**	-2.359214678	3.61E-20	9.11E-19	0.194897207	**Fahd2a**	-2.479684185	0.0002114	0.000591872	0.179283648
**Appl2**	-2.360740283	4.12E-19	8.41E-18	0.194691218	**Hfe**	-2.480112101	9.58E-18	1.53E-16	0.179230479
**Nckap5l**	-2.362594387	3.03E-17	4.46E-16	0.194441168	**Slc16a7**	-2.480907028	1.06E-19	2.42E-18	0.17913175
**Acss2**	-2.363251428	2.88E-16	3.57E-15	0.194352635	**Mfsd6**	-2.485278275	3.75E-17	5.44E-16	0.178589817
**Pstk**	-2.363501813	3.22E-11	1.98E-10	0.194318907	**Car7**	-2.485936079	3.31E-07	1.25E-06	0.178508406
**Gsn**	-2.365107553	1.05E-23	5.59E-22	0.194102748	**Atp23**	-2.487660369	3.15E-10	1.71E-09	0.178295183
**Itgb5**	-2.36695849	1.81E-24	1.16E-22	0.193853879	**Hykk**	-2.488142992	0.0027527	0.006768021	0.178235548
**Tpra1**	-2.374736014	1.39E-21	4.62E-20	0.192811631	**Tmem254c**	-2.490045312	5.61E-06	1.87E-05	0.178000684
**Pxmp4**	-2.376509172	5.35E-12	3.61E-11	0.192574799	**Gngt2**	-2.490923248	1.17E-20	3.26E-19	0.177892396
**Rab6b**	-2.379008509	2.05E-11	1.29E-10	0.19224147	**Gucy1a1**	-2.49272609	0.0041146	0.009892116	0.177670235
**Klhl21**	-2.380172928	5.87E-19	1.15E-17	0.192086372	**Snta1**	-2.493207599	3.15E-10	1.72E-09	0.177610946
**Smpd2**	-2.381045885	5.11E-13	3.97E-12	0.191970178	**Kcnq1**	-2.494494168	2.63E-08	1.13E-07	0.177452626
**Slc5a6**	-2.381997009	9.94E-13	7.40E-12	0.19184366	**Mypop**	-2.495151701	0.0001687	0.000477575	0.177371768
**Cby1**	-2.382879854	4.06E-11	2.46E-10	0.191726299	**D2hgdh**	-2.497880336	5.01E-14	4.45E-13	0.177036613
**Porcn**	-2.383155542	4.74E-05	0.00014242	0.191689665	**Klhdc1**	-2.498748712	3.80E-08	1.60E-07	0.176930085
**Cxcr1**	-2.383980143	7.19E-05	0.00021177	0.191580132	**Tm7sf3**	-2.505180376	4.26E-21	1.28E-19	0.17614307
**Ypel2**	-2.385279048	7.40E-14	6.39E-13	0.191407724	**Sel1l3**	-2.508071322	1.04E-19	2.37E-18	0.175790459
**Lmln**	-2.385786208	4.21E-07	1.57E-06	0.191340449	**Npas1**	-2.510177632	0.0037858	0.009148272	0.175533995
**Tmem65**	-2.385958791	2.19E-22	8.45E-21	0.191317561	**Trpm2**	-2.510339443	0.0001688	0.000477936	0.175514309
**Acot2**	-2.387668181	2.29E-15	2.50E-14	0.191091011	**Radx**	-2.511274529	1.41E-13	1.18E-12	0.175400586
**2510002D24Rik**	-2.389119256	1.09E-08	4.93E-08	0.190898907	**Psen2**	-2.514733967	7.99E-23	3.44E-21	0.174980497
**Mtr**	-2.389562873	4.64E-16	5.58E-15	0.190840216	**Fzd4**	-2.515583371	6.85E-12	4.56E-11	0.174877505
**B230118H07Rik**	-2.389779644	3.56E-12	2.45E-11	0.190811543	**Samd1**	-2.516866279	2.44E-20	6.33E-19	0.174722065
**Clybl**	-2.390654504	8.71E-11	5.09E-10	0.190695869	**Tle5**	-2.517264012	1.16E-24	7.82E-23	0.174673903
**Gstm4**	-2.392359403	2.78E-16	3.46E-15	0.190470648	**Endog**	-2.518894787	2.98E-08	1.27E-07	0.174476569
**Scrn3**	-2.396470992	1.72E-11	1.09E-10	0.189928591	**Arl4d**	-2.519391093	0.0001029	0.000298503	0.174416557
**Fahd1**	-2.398661409	1.71E-11	1.09E-10	0.189640445	**Ganc**	-2.519933771	6.16E-09	2.85E-08	0.174350962
**Lyl1**	-2.400749389	9.18E-23	3.86E-21	0.189366181	**Ldhb**	-2.521913521	5.53E-15	5.69E-14	0.174111872
**Speg**	-2.400928663	2.37E-10	1.31E-09	0.189342652	**Bcl2l12**	-2.523789559	1.32E-09	6.60E-09	0.173885609
**Cnrip1**	-2.40228566	2.04E-07	7.89E-07	0.18916464	**Tubb1**	-2.526264154	0.001524277	0.003871488	0.173587606
**Rasgef1a**	-2.404304803	6.31E-07	2.31E-06	0.188900077	**Map4k2**	-2.529325603	8.15E-07	2.96E-06	0.173219637
**Padi3**	-2.406239264	7.59E-06	2.49E-05	0.188646957	**Fgd4**	-2.530029897	4.32E-19	8.80E-18	0.173135096
**Parpbp**	-2.407372033	0.0002943	0.00080941	0.188498894	**Gas1**	-2.530271117	1.95E-08	8.50E-08	0.17310615
**Tm7sf2**	-2.408322099	1.41E-07	5.57E-07	0.188374802	**Cfap410**	-2.532294618	2.01E-16	2.57E-15	0.172863524
**Castor2**	-2.41029282	6.91E-11	4.08E-10	0.188117658	**Car9**	-2.534683177	6.86E-11	4.05E-10	0.172577564
**C77080**	-2.411237693	1.65E-22	6.49E-21	0.187994493	**Triqk**	-2.537853763	0.0020984	0.005244836	0.17219871
**Jph3**	-2.413084752	0.0017723	0.00446807	0.187753961	**Mfsd3**	-2.538532465	1.47E-12	1.07E-11	0.172117719
**Acap3**	-2.41572884	3.21E-13	2.56E-12	0.187410171	**Frat1**	-2.539386029	4.11E-05	0.00012435	0.172015917
**Glul**	-2.416112141	9.60E-24	5.13E-22	0.187360386	**Cenpu**	-2.543691437	8.02E-05	0.000235523	0.171503338
**Mxd4**	-2.41699522	5.15E-15	5.31E-14	0.187245737	**Jade1**	-2.54413733	1.28E-14	1.25E-13	0.17145034
**Nxpe4**	-2.417183666	6.24E-08	2.57E-07	0.187221281	**Syne1**	-2.54775317	7.76E-16	9.03E-15	0.17102117
**Nars2**	-2.417596362	3.08E-13	2.46E-12	0.187167732	**Khk**	-2.549218419	1.09E-13	9.20E-13	0.170847564
**Anks6**	-2.421177853	0.0003487	0.00095051	0.186703664	**Gnb1l**	-2.551276531	7.44E-06	2.44E-05	0.170604011
**Zfp362**	-2.422309006	1.29E-15	1.45E-14	0.186557336	**Cenpk**	-2.555225897	0.0040008	0.009636586	0.170137622
**Ehhadh**	-2.42299723	1.13E-07	4.52E-07	0.186468361	**Zrsr1**	-2.555619699	1.93E-07	7.49E-07	0.170091187
**6720489N17Rik**	-2.42356068	4.21E-08	1.77E-07	0.18639555	**Tef**	-2.555823943	3.61E-16	4.40E-15	0.170067109
**Acot13**	-2.424222901	1.42E-16	1.85E-15	0.186310011	**Cbx6**	-2.556796988	1.30E-06	4.60E-06	0.169952444
**Vmac**	-2.424682185	3.04E-07	1.15E-06	0.186250708	**Hddc2**	-2.558048755	5.81E-09	2.70E-08	0.169805047
**Acad12**	-2.425508908	1.79E-14	1.70E-13	0.186144009	**Vars2**	-2.558309936	3.46E-07	1.31E-06	0.169774309
**Tgfbr2**	-2.426696373	3.09E-24	1.85E-22	0.185990859	**Hsd17b1**	-2.561565951	2.96E-05	9.11E-05	0.169391578
**Mrnip**	-2.42723268	0.0006340	0.00167676	0.185921732	**Clec2i**	-2.564130328	2.59E-16	3.24E-15	0.169090753
**Slc18b1**	-2.433251196	5.54E-06	1.85E-05	0.185147735	**Gcsh**	-2.565325795	3.25E-20	8.23E-19	0.168950697
**Tpmt**	-2.436394724	4.60E-10	2.46E-09	0.18474475	**Rnase6**	-2.565411402	1.17E-09	5.92E-09	0.168940672
**Tmem107**	-2.437794976	4.16E-06	1.41E-05	0.184565528	**Gmnn**	-2.569091631	4.97E-13	3.88E-12	0.168510263
**Lrrc56**	-2.43963617	5.16E-08	2.14E-07	0.184330132	**Relt**	-2.569848181	1.78E-15	1.97E-14	0.16842192
**Alox5ap**	-2.44395818	4.66E-23	2.08E-21	0.183778744	**Zfp532**	-2.573907739	3.71E-05	0.000112678	0.167948668
**Dusp13**	-2.444182204	1.21E-05	3.89E-05	0.183750209	**Adi1**	-2.576109534	8.14E-21	2.31E-19	0.167692546
**Ccdc85b**	-2.446082626	4.40E-15	4.58E-14	0.183508319	**Cbs**	-2.576352597	0.0011370	0.002934333	0.167664296
**Mapre3**	-2.446190076	1.56E-12	1.14E-11	0.183494652	**Nceh1**	-2.577551683	3.32E-26	3.27E-24	0.167525001

Gene	log2 (FC)	P-value	FDR	FC	Gene	log2 (FC)	P-value	FDR	FC
**Gdpd3**	-2.577903619	0.0010170	0.00263798	0.167484139	**Pla2g15**	-2.74526524	6.98E-25	4.96E-23	0.149139546
**Nme4**	-2.581606182	8.11E-08	3.29E-07	0.167054855	**Mapk14**	-2.748102736	5.69E-25	4.14E-23	0.148846506
**Ttc38**	-2.584434035	1.93E-14	1.83E-13	0.166727729	**Cryz**	-2.750746259	6.95E-14	6.03E-13	0.148574017
**Rnd2**	-2.584640417	6.29E-05	0.00018646	0.166703879	**B630019K06Rik**	-2.751336807	2.55E-06	8.79E-06	0.148513213
**Mcrip2**	-2.585178739	6.27E-11	3.72E-10	0.166641688	**Tnfaip8l1**	-2.753320604	5.58E-10	2.95E-09	0.148309138
**Galc**	-2.586274895	1.52E-23	7.63E-22	0.166515122	**Matn2**	-2.755504674	2.07E-16	2.63E-15	0.148084785
**Spef1**	-2.588372082	1.45E-11	9.31E-11	0.166273242	**Bcl7a**	-2.75923245	0.0003894	0.001056205	0.147702643
**Pcp4l1**	-2.593124213	0.0016698	0.00421930	0.165726451	**Myo1f**	-2.760262161	4.52E-28	8.24E-26	0.147597259
**Smim4**	-2.595282646	1.32E-09	6.63E-09	0.165478691	**Abhd8**	-2.761598476	1.68E-16	2.18E-15	0.147460609
**Poglut3**	-2.595495121	2.10E-16	2.66E-15	0.165454321	**Sgsm2**	-2.762809562	6.28E-13	4.81E-12	0.147336873
**S1pr1**	-2.598386141	1.52E-20	4.15E-19	0.165123099	**Sptssa**	-2.764564793	4.57E-23	2.05E-21	0.147157727
**Dbndd2**	-2.600653381	0.0005871	0.00155978	0.164863807	**Gtf2i**	-2.766632906	8.30E-21	2.35E-19	0.146946927
**Sfxn5**	-2.602042594	1.93E-14	1.83E-13	0.164705131	**Slc9a9**	-2.768111889	2.43E-11	1.51E-10	0.146796361
**Zfp30**	-2.606727672	0.0035924	0.00870897	0.164171127	**Selenoh**	-2.76885724	4.70E-13	3.68E-12	0.14672054
**Clec4b1**	-2.615762668	6.00E-05	0.00017831	0.163146205	**Gpr162**	-2.771028492	1.16E-07	4.63E-07	0.146499892
**Agap1**	-2.617940639	1.20E-19	2.70E-18	0.162900096	**Tspan32**	-2.773061705	1.89E-15	2.08E-14	0.146293573
**Zscan18**	-2.620144509	0.0016508	0.00417489	0.162651439	**Fli1**	-2.774205302	8.41E-20	1.96E-18	0.146177655
**Ttll1**	-2.621290288	3.47E-12	2.40E-11	0.162522314	**Smim1**	-2.776632583	0.0003592	0.000977378	0.145931923
**Fry**	-2.621403787	3.94E-17	5.70E-16	0.162509528	**Pik3r2**	-2.777460648	7.98E-19	1.53E-17	0.145848186
**Klhl6**	-2.621592307	7.28E-16	8.52E-15	0.162488294	**Pomgnt2**	-2.777608354	5.83E-06	1.94E-05	0.145833255
**Pcyox1l**	-2.622233499	2.94E-10	1.60E-09	0.162416094	**Rnf186**	-2.777703496	0.0021961	0.005475837	0.145823638
**Enthd1**	-2.622359715	0.0007805	0.00204819	0.162401885	**Fam234b**	-2.782605881	3.64E-15	3.84E-14	0.145328959
**Phka1**	-2.623128405	1.43E-08	6.33E-08	0.162315378	**Zfp114**	-2.79213779	0.0001569	0.000445682	0.144371934
**Eya4**	-2.624601635	9.50E-14	8.10E-13	0.162149712	**P2ry12**	-2.800187571	0.0001548	0.00044007	0.143568627
**Kif18a**	-2.625483543	3.90E-05	0.00011812	0.162050621	**Fam53b**	-2.80291223	1.74E-21	5.69E-20	0.143297741
**Cd59a**	-2.628074058	1.45E-05	4.61E-05	0.161759903	**Etfbkmt**	-2.803995348	1.11E-08	4.98E-08	0.143190199
**Tmem144**	-2.628107326	4.18E-12	2.86E-11	0.161756173	**Kcng2**	-2.807827724	7.43E-05	0.000218494	0.142810333
**Sh3d19**	-2.628743753	6.65E-15	6.74E-14	0.161684832	**Rab4a**	-2.810754287	3.31E-07	1.25E-06	0.142520931
**Camk1**	-2.629007028	2.76E-22	1.04E-20	0.161655329	**Dnajb13**	-2.817388915	4.73E-06	1.59E-05	0.141867014
**Kif5a**	-2.633390617	0.0002902	0.00079897	0.161164889	**Ajuba**	-2.81744585	3.35E-06	1.14E-05	0.141861415
**Clec4a2**	-2.637630614	3.32E-21	1.02E-19	0.16069193	**Gabbr2**	-2.817796043	0.0024009	0.005952408	0.141826985
**Msh2**	-2.640250464	9.51E-15	9.43E-14	0.160400388	**Nkain1**	-2.821792315	1.68E-08	7.39E-08	0.141434667
**Habp4**	-2.644055475	4.56E-18	7.64E-17	0.1599779	**Hdac11**	-2.822628822	0.0005641	0.001502105	0.141352684
**Phospho1**	-2.645666474	1.82E-18	3.28E-17	0.159799359	**Akr7a5**	-2.823683867	1.12E-15	1.27E-14	0.14124935
**Sh2d1b1**	-2.647495509	5.31E-10	2.81E-09	0.159596895	**Pccb**	-2.831656528	7.82E-19	1.50E-17	0.140470927
**Creg2**	-2.653628342	2.81E-17	4.17E-16	0.158919896	**Hnmt**	-2.832859425	1.83E-13	1.51E-12	0.140353853
**Dnajc28**	-2.665135805	2.74E-08	1.18E-07	0.157657335	**Fmo5**	-2.837261993	1.48E-11	9.45E-11	0.139926198
**Sptb**	-2.666197794	0.0017375	0.00438319	0.157541324	**Rps6kl1**	-2.837433569	0.0003107	0.000851656	0.139909558
**B3gnt8**	-2.666760455	2.82E-12	1.97E-11	0.157479893	**Fam186b**	-2.839583483	0.0027390	0.006738007	0.139701219
**Ier5l**	-2.668295636	8.90E-10	4.57E-09	0.157312407	**Mgmt**	-2.84178885	8.14E-10	4.21E-09	0.139487829
**Fam214a**	-2.668905576	9.04E-06	2.94E-05	0.157245913	**Gnb5**	-2.842222626	1.10E-07	4.40E-07	0.139445895
**Rtkn**	-2.672679567	0.0003247	0.00088764	0.156835106	**Atp2a3**	-2.842269808	3.01E-19	6.29E-18	0.139441335
**Serpinb12**	-2.67526083	0.0007643	0.00200699	0.156554748	**Gsta3**	-2.842950899	1.94E-11	1.22E-10	0.139375521
**B230217C12Rik**	-2.67814883	0.0001453	0.00041477	0.156241669	**Adcy3**	-2.85081723	2.51E-06	8.68E-06	0.13861764
**Tfdp2**	-2.678963145	1.12E-08	5.06E-08	0.156153505	**Dnph1**	-2.859759975	3.53E-09	1.69E-08	0.137761057
**Bphl**	-2.679534388	1.90E-11	1.20E-10	0.156091687	**Sbk1**	-2.859899657	6.82E-16	8.03E-15	0.13774772
**Vsir**	-2.680881237	2.70E-25	2.06E-23	0.155946033	**Sdk2**	-2.860705236	3.62E-06	1.23E-05	0.137670825
**Slc29a2**	-2.681534095	2.11E-08	9.16E-08	0.15587548	**Sult1a1**	-2.861100253	0.0036594	0.00885755	0.137633135
**Ip6k3**	-2.68541067	2.68E-05	8.28E-05	0.155457199	**Gm14308**	-2.866574885	5.91E-05	0.000175604	0.137111845
**C130050O18Rik**	-2.685812527	4.96E-10	2.64E-09	0.155413903	**Tbc1d16**	-2.867268093	3.92E-12	2.69E-11	0.137045979
**Arhgap33**	-2.686538052	0.0003871	0.00105028	0.155335765	**Gata5**	-2.874002473	0.0018828	0.004734945	0.136407751
**Mettl8**	-2.690316883	1.52E-09	7.56E-09	0.154929429	**Rnf227**	-2.87685272	2.21E-11	1.38E-10	0.136138524
**Lipa**	-2.693299562	6.63E-28	1.11E-25	0.154609453	**Klf15**	-2.880325137	0.0007457	0.001959758	0.135811247
**Gpr65**	-2.696019563	1.23E-19	2.76E-18	0.154318233	**N4bp3**	-2.882067677	4.18E-16	5.06E-15	0.135647308
**Dact3**	-2.697264899	0.0002991	0.00082183	0.154185083	**B3gnt7**	-2.884908132	2.52E-09	1.23E-08	0.135380501
**Prxl2b**	-2.702523818	1.38E-18	2.56E-17	0.15362407	**Zfp692**	-2.888433842	2.74E-10	1.50E-09	0.135050057
**Tmem198b**	-2.704309078	3.52E-11	2.15E-10	0.153434086	**Gm14399**	-2.889917435	1.17E-08	5.25E-08	0.13491125
**Nlrc3**	-2.706254639	9.72E-17	1.30E-15	0.15322731	**Adrb1**	-2.890340623	0.0019784	0.004960909	0.134871682
**Ccdc142**	-2.708570857	3.30E-05	0.00010074	0.152981504	**Scnn1a**	-2.893410574	0.0002981	0.000819343	0.13458499
**Manba**	-2.709646032	2.72E-22	1.02E-20	0.152867536	**Adam11**	-2.902231207	0.0036436	0.008823256	0.133764648
**Metrn**	-2.709762497	5.82E-14	5.10E-13	0.152855196	**Lpar6**	-2.903168124	2.55E-18	4.48E-17	0.133677807
**Clec4a3**	-2.709870257	2.48E-22	9.42E-21	0.152843779	**Npas2**	-2.907348854	0.0009514	0.002474212	0.133290988
**Gpr155**	-2.711477483	6.78E-09	3.12E-08	0.1526736	**Slc2a8**	-2.907719629	6.06E-15	6.19E-14	0.133256736
**Ap5s1**	-2.71199643	1.05E-17	1.66E-16	0.152618692	**Iqce**	-2.911257978	3.96E-12	2.72E-11	0.132930312
**Vsig10**	-2.714490512	5.66E-05	0.000168898	0.152355078	**Hoga1**	-2.91185393	0.000926476	0.002414516	0.132875412
**Add3**	-2.714651732	4.13E-21	1.24E-19	0.152338053	**Ppargc1b**	-2.915945312	4.25E-11	2.56E-10	0.13249912
**Sorbs3**	-2.716772858	2.34E-24	1.45E-22	0.152114242	**Fos**	-2.916712644	2.24E-19	4.79E-18	0.132428666
**Ccdc69**	-2.717962651	5.66E-10	2.99E-09	0.151988845	**Shpk**	-2.930422906	9.96E-08	4.01E-07	0.131176127
**Cr2**	-2.718946012	0.0018098	0.00455878	0.151885283	**Gamt**	-2.932517778	3.65E-10	1.98E-09	0.130985791
**Klhl38**	-2.719808252	3.57E-05	0.00010878	0.151794534	**Ak8**	-2.932557784	5.32E-09	2.49E-08	0.130982159
**Epb41l1**	-2.72614503	1.17E-22	4.78E-21	0.151129266	**Gpr160**	-2.933647995	3.69E-14	3.35E-13	0.130883216
**Osgepl1**	-2.727677229	0.0001419	0.00040555	0.150968846	**Zfp934**	-2.934266826	6.53E-08	2.68E-07	0.130827087
**Spry3**	-2.730097748	0.0004277	0.00115380	0.150715767	**Cdkn2c**	-2.934962184	2.19E-10	1.21E-09	0.130764045
**Rasgrp2**	-2.737169697	2.40E-10	1.32E-09	0.14997878	**Ankdd1a**	-2.934962453	1.25E-06	4.45E-06	0.130764021
**Acot1**	-2.738088369	0.000266966	0.00073901	0.149883308	**Aldh9a1**	-2.935817083	2.35E-24	1.45E-22	0.130686581
**Mtfp1**	-2.73897189	1.02E-07	4.10E-07	0.149791546	**Mrvi1**	-2.939150241	0.0013790	0.003526094	0.130384995
**Vps13c**	-2.739252169	8.19E-18	1.32E-16	0.149762448	**Smad6**	-2.940987023	4.88E-14	4.34E-13	0.1302191
**Brca2**	-2.7406689	9.06E-07	3.27E-06	0.149615453	**Abca2**	-2.942056082	6.02E-17	8.37E-16	0.130122641
**Bdh1**	-2.743066101	4.03E-07	1.51E-06	0.149367057	**Zfp647**	-2.943047864	1.21E-06	4.32E-06	0.130033219
**Tmem51**	-2.743120861	1.46E-18	2.70E-17	0.149361387	**Bmyc**	-2.946916508	4.65E-10	2.48E-09	0.129684997
**Tifab**	-2.74460511	4.73E-21	1.41E-19	0.149207803	**Fam78b**	-2.947967549	4.51E-11	2.71E-10	0.129590552

Gene	log2 (FC)	P-value	FDR	FC	Gene	log2 (FC)	P-value	FDR	FC
**Adamts15**	-2.94818851	0.0034845	0.00845892	0.129570706	**Epm2a**	-3.225265341	1.15E-08	5.15E-08	0.10692971
**Npl**	-2.949242664	7.01E-10	3.65E-09	0.129476065	**Tmem154**	-3.229117325	2.35E-23	1.12E-21	0.106644589
**Aamdc**	-2.94970397	2.05E-10	1.14E-09	0.129434672	**Smyd3**	-3.231335201	4.89E-13	3.82E-12	0.106480769
**Mical3**	-2.950330254	6.66E-10	3.48E-09	0.129378495	**Scamp5**	-3.238534669	6.45E-14	5.63E-13	0.105950723
**Coro2b**	-2.959859461	0.0015258	0.00387489	0.128526748	**Spaca9**	-3.245357375	6.56E-08	2.69E-07	0.10545085
**Stxbp4**	-2.959869507	4.82E-07	1.79E-06	0.128525853	**Slc46a1**	-3.245372288	4.91E-10	2.61E-09	0.10544976
**Impa2**	-2.965318711	1.23E-12	9.06E-12	0.128041314	**Hacd4**	-3.24647165	1.46E-23	7.43E-22	0.105369435
**Nfatc2**	-2.966415779	1.21E-07	4.82E-07	0.127943984	**Ceacam19**	-3.248274992	2.42E-17	3.63E-16	0.105237808
**Recql4**	-2.970723018	1.42E-08	6.30E-08	0.127562571	**Ccdc166**	-3.261589385	9.99E-10	5.10E-09	0.104271054
**Pgm2l1**	-2.987132717	6.60E-16	7.80E-15	0.126119852	**Patl2**	-3.265887622	4.24E-05	0.00012806	0.10396086
**Elovl6**	-2.988361545	4.38E-15	4.56E-14	0.126012474	**Pyroxd2**	-3.266242947	0.0019395	0.004868049	0.103935258
**Ttc7**	-2.990587635	9.93E-25	6.77E-23	0.125818185	**Nipal3**	-3.272273603	8.28E-17	1.13E-15	0.103501702
**Plekhh3**	-2.991431282	2.28E-14	2.14E-13	0.125744632	**Ttyh2**	-3.27373166	2.03E-25	1.63E-23	0.103397151
**Hdhd3**	-2.995143446	2.44E-08	1.05E-07	0.125421497	**Hebp1**	-3.277617586	2.89E-21	9.01E-20	0.103119024
**Dennd2c**	-2.997152677	4.10E-06	1.39E-05	0.125246945	**Rragd**	-3.288128207	2.16E-20	5.66E-19	0.10237049
**Fam83f**	-2.998362934	1.64E-15	1.83E-14	0.125141921	**Nlrp1a**	-3.290667838	1.14E-08	5.12E-08	0.102190441
**Adprhl1**	-3.005261212	0.0040097	0.00965513	0.124544981	**Pcyt1b**	-3.293824735	0.0002104	0.00058941	0.101967073
**Abca9**	-3.011525195	3.62E-14	3.29E-13	0.124005396	**Snx29**	-3.294866186	1.33E-16	1.75E-15	0.101893492
**Ints6l**	-3.013674627	2.21E-17	3.33E-16	0.123820781	**H2bc21**	-3.296427092	5.33E-17	7.50E-16	0.101783309
**Idh2**	-3.014306441	1.48E-22	5.87E-21	0.123766567	**Tmem71**	-3.298487631	2.63E-14	2.44E-13	0.10163804
**Haus4**	-3.015047348	4.20E-12	2.87E-11	0.123703022	**Tmem218**	-3.29967104	8.23E-12	5.43E-11	0.101554703
**Inpp5j**	-3.015669053	0.0005496	0.00146476	0.123649726	**Susd3**	-3.304113248	6.07E-16	7.21E-15	0.101242487
**Nipsnap1**	-3.018225554	3.63E-07	1.37E-06	0.123430809	**Mccc2**	-3.304603113	3.34E-14	3.05E-13	0.101208116
**Mcoln3**	-3.023038655	3.05E-23	1.41E-21	0.123019707	**Hoxa4**	-3.305681383	0.0025603	0.006322917	0.101132501
**Pnma1**	-3.02427255	0.0006954	0.00183170	0.122914536	**Unc13a**	-3.30837781	1.25E-11	8.10E-11	0.100943659
**D630023F18Rik**	-3.026446408	0.0019171	0.00481508	0.122729468	**Tfeb**	-3.309836474	5.60E-24	3.20E-22	0.100841649
**Aqp11**	-3.026880362	0.0001037	0.00030068	0.122692557	**Clmn**	-3.314231823	1.17E-05	3.75E-05	0.10053489
**Naip1**	-3.028402903	5.43E-13	4.20E-12	0.122563142	**Scd2**	-3.3165788	4.71E-22	1.71E-20	0.100371473
**Paqr7**	-3.03347299	1.94E-21	6.27E-20	0.122133173	**Pnpla7**	-3.32065196	7.85E-23	3.41E-21	0.100088494
**Zdhhc14**	-3.036910002	8.12E-12	5.36E-11	0.121842554	**Tacc2**	-3.324867042	2.93E-11	1.81E-10	0.099796495
**Ldlrad3**	-3.037555774	4.92E-19	9.83E-18	0.121788028	**Lyplal1**	-3.331003902	8.79E-08	3.56E-07	0.099372888
**Dtwd2**	-3.039579222	6.45E-07	2.36E-06	0.121617334	**Bsn**	-3.335952625	3.09E-15	3.30E-14	0.099032603
**Phlda3**	-3.041153635	5.99E-13	4.60E-12	0.121484686	**Mr1**	-3.34476304	9.80E-12	6.40E-11	0.098429663
**Ankrd55**	-3.041519623	7.44E-13	5.64E-12	0.121453871	**Gm14403**	-3.345557724	4.28E-08	1.79E-07	0.098375459
**Brd3os**	-3.042356241	4.02E-07	1.50E-06	0.12138346	**Kifc3**	-3.350386692	2.69E-16	3.36E-15	0.098046729
**Nynrin**	-3.043897303	1.31E-07	5.16E-07	0.12125387	**Cped1**	-3.353977147	2.35E-14	2.20E-13	0.097803022
**Arvcf**	-3.051113103	1.19E-07	4.72E-07	0.120648919	**Dlg2**	-3.354182643	0.0001297	0.000372063	0.097789092
**Abhd14b**	-3.051577115	3.83E-15	4.03E-14	0.120610121	**Angpt2**	-3.357727534	7.41E-08	3.02E-07	0.097549106
**Aspa**	-3.05288286	4.36E-05	0.00013141	0.12050101	**Lair1**	-3.358855687	1.25E-22	5.08E-21	0.097472855
**Ctnnbip1**	-3.057932095	7.03E-14	6.09E-13	0.12008001	**Fads1**	-3.363344242	2.26E-21	7.20E-20	0.097170066
**Kctd12b**	-3.067525855	1.57E-14	1.51E-13	0.11928414	**Nkapl**	-3.367772972	0.0006597	0.001741766	0.096872234
**Kazald1**	-3.069618866	3.17E-05	9.72E-05	0.119111213	**Snx32**	-3.380894168	7.97E-10	4.13E-09	0.095995184
**Il6ra**	-3.069954813	9.67E-23	4.06E-21	0.11908348	**Pkn3**	-3.388732996	7.21E-08	2.95E-07	0.095475012
**Pank1**	-3.073767993	4.43E-09	2.08E-08	0.118769146	**Dmrta2**	-3.39938789	0.0006104	0.001617321	0.094772487
**Rab3a**	-3.076626695	1.19E-06	4.26E-06	0.118534038	**Tle2**	-3.401944503	1.85E-05	5.82E-05	0.094604689
**Fblim1**	-3.076802677	7.93E-25	5.53E-23	0.11851958	**Zc4h2**	-3.405994326	2.23E-12	1.58E-11	0.094339494
**Nt5dc2**	-3.077549494	2.15E-18	3.83E-17	0.118458244	**Mlxipl**	-3.410338259	0.0026079	0.00643161	0.094055866
**L3hypdh**	-3.07818884	0.0003446	0.00093972	0.118405759	**Cela1**	-3.411674328	6.18E-08	2.55E-07	0.093968802
**Hacd1**	-3.079029436	1.07E-12	7.93E-12	0.118336789	**Mixl1**	-3.415905212	0.001027291	0.002664444	0.093693631
**E2f2**	-3.083817005	1.72E-12	1.24E-11	0.117944741	**Cd300a**	-3.421035289	4.87E-27	6.13E-25	0.093361057
**Calml4**	-3.09063954	0.002486728	0.006152409	0.117388294	**Ypel3**	-3.422059005	1.19E-20	3.29E-19	0.093294833
**Gga2**	-3.10536403	4.41E-08	1.84E-07	0.116196295	**Selenbp1**	-3.425675011	1.52E-13	1.27E-12	0.09306129
**Fbxo31**	-3.105778452	8.58E-15	8.59E-14	0.116162922	**Fhdc1**	-3.432024652	1.19E-10	6.85E-10	0.092652605
**Zfp2**	-3.108655682	5.73E-05	0.00017081	0.115931484	**Arsg**	-3.457155414	2.42E-23	1.15E-21	0.091052635
**Cxcr4**	-3.110905251	1.31E-24	8.69E-23	0.115750855	**Gab3**	-3.457939415	1.25E-08	5.62E-08	0.091003168
**Tarsl2**	-3.112605924	5.94E-12	3.99E-11	0.115614486	**1810010H24Rik**	-3.458673982	0.001660237	0.004197147	0.090956845
**Pacc1**	-3.115175196	5.50E-22	1.96E-20	0.115408773	**Atp13a2**	-3.461321386	1.38E-24	9.13E-23	0.090790088
**Cox6b2**	-3.125162852	0.0006687	0.00176483	0.114612567	**Adamts10**	-3.462555994	1.87E-09	9.23E-09	0.090712426
**Ulk4**	-3.12618398	0.0003990	0.00108060	0.114531474	**Fam117a**	-3.467260663	3.80E-15	4.00E-14	0.090417093
**Arl11**	-3.142196189	8.44E-24	4.60E-22	0.113267338	**Trim29**	-3.46854288	1.55E-13	1.29E-12	0.090336769
**Tns4**	-3.143226001	1.20E-10	6.86E-10	0.113186516	**Polr3g**	-3.470072091	5.89E-10	3.10E-09	0.090241065
**Zfand4**	-3.14545074	0.0012327	0.00316869	0.113012108	**Prune2**	-3.478908969	2.71E-13	2.18E-12	0.089690005
**Arhgef39**	-3.14587539	0.0027344	0.00672815	0.112978849	**P2ry1**	-3.485818506	4.83E-18	8.03E-17	0.089261477
**Rgs18**	-3.150191975	4.51E-15	4.69E-14	0.112641318	**Daglb**	-3.491703601	7.05E-24	3.90E-22	0.0888981
**N4bp2l1**	-3.151258871	4.51E-17	6.41E-16	0.112558049	**Fam161a**	-3.494774153	2.19E-06	7.62E-06	0.088709096
**Gcnt1**	-3.156253923	4.85E-19	9.73E-18	0.112169012	**Nfia**	-3.496777937	5.87E-09	2.72E-08	0.088585972
**Acp6**	-3.158495496	1.09E-15	1.24E-14	0.111994866	**Fut7**	-3.502413725	5.43E-11	3.24E-10	0.088240592
**Bmf**	-3.164398896	9.26E-12	6.08E-11	0.111537528	**Aldoc**	-3.507119821	3.13E-08	1.33E-07	0.087953218
**Dzank1**	-3.16541589	0.0034799	0.00844853	0.11145893	**Fcor**	-3.511986221	1.01E-05	3.27E-05	0.08765704
**Nuak1**	-3.166685346	1.52E-13	1.26E-12	0.111360898	**Nicn1**	-3.526735036	3.69E-14	3.35E-13	0.086765479
**Rcbtb2**	-3.170841719	5.73E-18	9.42E-17	0.111040531	**Tbc1d4**	-3.529796511	6.38E-09	2.95E-08	0.086581553
**Naprt**	-3.180140228	8.11E-07	2.94E-06	0.11032715	**Smarca2**	-3.538664954	7.10E-12	4.72E-11	0.086050957
**Haghl**	-3.184640535	6.49E-14	5.66E-13	0.109983535	**9930012K11Rik**	-3.538875472	1.51E-05	4.78E-05	0.086038401
**Tlr5**	-3.189028859	0.0001140	0.00032905	0.1096495	**Trnp1**	-3.53932024	1.67E-07	6.52E-07	0.086011881
**Wfikkn1**	-3.197822798	0.0003545	0.00096548	0.108983165	**Tsc22d3**	-3.540335946	2.01E-24	1.28E-22	0.085951347
**Rab3il1**	-3.201644551	2.94E-17	4.33E-16	0.108694847	**Myl6b**	-3.540603723	4.79E-07	1.78E-06	0.085935395
**Fads6**	-3.207501894	2.76E-05	8.53E-05	0.10825444	**Cdh1**	-3.54691248	1.79E-08	7.87E-08	0.085560429
**Paox**	-3.208365502	2.18E-17	3.29E-16	0.108189658	**Sort1**	-3.548458154	3.06E-24	1.84E-22	0.08546881
**Dync2li1**	-3.210406854	3.52E-09	1.68E-08	0.108036682	**Peli2**	-3.549656857	5.81E-16	6.92E-15	0.085397825
**Rnf150**	-3.211817474	7.05E-10	3.67E-09	0.107931099	**Fastkd3**	-3.552152849	3.53E-06	1.20E-05	0.085250207
**Slc9a3r2**	-3.215249884	6.55E-10	3.42E-09	0.107674618	**Gpr157**	-3.555585927	3.82E-20	9.62E-19	0.085047585

Gene	log2 (FC)	P-value	FDR	FC	Gene	log2 (FC)	P-value	FDR	FC
**Epas1**	-3.555719331	9.02E-24	4.86E-22	0.085039721	**Akap5**	-4.062655182	0.0039604	0.009545795	0.059843768
**Foxred2**	-3.557109384	2.19E-19	4.71E-18	0.084957823	**Adssl1**	-4.075896857	3.84E-24	2.26E-22	0.059297009
**Pianp**	-3.57213996	2.49E-20	6.45E-19	0.084077294	**Idh1**	-4.079130182	1.43E-27	2.12E-25	0.059164263
**Egfl8**	-3.573237978	0.0015488	0.00392954	0.084013328	**Vash2**	-4.089185398	5.69E-10	3.00E-09	0.058753337
**Tpk1**	-3.574669836	7.34E-09	3.37E-08	0.083929987	**Clec1b**	-4.094006889	5.47E-06	1.82E-05	0.058557311
**Chn2**	-3.578677275	1.84E-09	9.07E-09	0.083697174	**Rnf128**	-4.100511745	2.04E-30	1.08E-27	0.058293881
**Acot6**	-3.581550812	5.83E-13	4.49E-12	0.083530633	**Aph1c**	-4.102919985	1.58E-25	1.32E-23	0.058196654
**Cnnm2**	-3.583223601	1.19E-12	8.79E-12	0.083433837	**Sbk2**	-4.111727905	3.31E-15	3.51E-14	0.057842435
**Ydjc**	-3.589286445	4.86E-11	2.92E-10	0.083083946	**Pkd1l2**	-4.130249272	1.29E-07	5.09E-07	0.057104598
**Marveld1**	-3.596070546	1.19E-15	1.34E-14	0.082694171	**Pcdhga6**	-4.131377957	0.0036065	0.008740406	0.05705994
**Rnft2**	-3.628023906	7.87E-05	0.00023111	0.080882763	**Slc37a2**	-4.13722224	1.29E-26	1.42E-24	0.056829261
**Fndc10**	-3.633611004	1.08E-06	3.88E-06	0.080570136	**Rab42**	-4.156005479	0.0016285	0.004122728	0.056094165
**Etl4**	-3.637613904	0.0001161	0.00033492	0.080346896	**Slc46a3**	-4.166876009	2.92E-17	4.32E-16	0.055673091
**Dchs1**	-3.639767688	2.28E-07	8.79E-07	0.080227036	**Prkag2**	-4.175754753	2.35E-19	5.01E-18	0.055331515
**Hadh**	-3.666107173	4.94E-20	1.21E-18	0.07877561	**Trim2**	-4.194207575	4.97E-11	2.98E-10	0.054628303
**Ccsap**	-3.666431522	2.45E-08	1.06E-07	0.078757901	**Zfp791**	-4.198751881	6.89E-05	0.000203218	0.054456502
**Dglucy**	-3.674947649	7.61E-19	1.46E-17	0.078294368	**Krt80**	-4.199549476	2.75E-17	4.08E-16	0.054426404
**Tmod1**	-3.677525706	7.56E-08	3.08E-07	0.078154583	**Pparg**	-4.210321975	2.02E-18	3.60E-17	0.054021519
**Lrmp**	-3.684593498	4.69E-18	7.83E-17	0.077772639	**Cx3cr1**	-4.215838421	1.74E-11	1.10E-10	0.053815351
**Grin3b**	-3.68552731	0.0002575	0.00071415	0.077722315	**Cnr2**	-4.218923092	1.96E-19	4.25E-18	0.05370041
**Stard9**	-3.712783411	3.58E-09	1.71E-08	0.076267731	**Plxdc1**	-4.224910406	5.50E-19	1.09E-17	0.05347801
**Tmem37**	-3.714373406	2.03E-17	3.09E-16	0.076183723	**Tle6**	-4.27053478	1.80E-05	5.67E-05	0.051813262
**Hyal1**	-3.717085848	1.15E-14	1.13E-13	0.076040622	**6430548M08Rik**	-4.288865408	1.86E-23	9.15E-22	0.051159096
**Zc2hc1c**	-3.71711043	6.42E-05	0.00019019	0.076039327	**Tmem273**	-4.298583871	1.24E-13	1.04E-12	0.05081563
**Fbxo32**	-3.738823079	4.92E-19	9.83E-18	0.074903499	**Tmem114**	-4.300455205	1.35E-05	4.33E-05	0.050749759
**Plxna3**	-3.739872171	1.24E-06	4.41E-06	0.074849051	**Gmpr**	-4.310440222	2.77E-08	1.18E-07	0.050399729
**Nrcam**	-3.743449172	0.0022808	0.00567257	0.074663701	**Snx24**	-4.310736961	1.73E-20	4.67E-19	0.050389363
**Tmem150b**	-3.749404538	1.68E-08	7.41E-08	0.074356128	**Hal**	-4.319629634	7.07E-13	5.38E-12	0.050079722
**Abi3**	-3.749877875	3.57E-18	6.14E-17	0.074331737	**Aatk**	-4.327969262	9.60E-20	2.20E-18	0.049791067
**Spns2**	-3.773208904	0.0006092	0.00161503	0.073139324	**Gstm7**	-4.343693274	0.001109977	0.002867584	0.049251338
**Prrt3**	-3.780813474	0.0003107	0.00085166	0.072754814	**Tmem82**	-4.344064862	3.42E-09	1.64E-08	0.049238654
**Hdac5**	-3.782543511	2.83E-14	2.61E-13	0.072667621	**Fam78a**	-4.344343131	3.10E-18	5.39E-17	0.049229158
**Cd93**	-3.782728458	8.63E-24	4.69E-22	0.072658306	**Slc13a2**	-4.345302953	0.0014507	0.003694943	0.049196417
**Neurl2**	-3.789901437	9.33E-12	6.12E-11	0.072297951	**Eef2k**	-4.354346427	1.95E-17	2.96E-16	0.048888995
**Fam229b**	-3.793255767	1.77E-06	6.19E-06	0.07213005	**Mafb**	-4.354687315	7.53E-28	1.22E-25	0.048877445
**Tpcn1**	-3.799100637	1.13E-17	1.77E-16	0.071838417	**Aloxe3**	-4.361944158	0.0007641	0.002006804	0.048632206
**Tst**	-3.79930783	2.42E-20	6.30E-19	0.0718281	**Plscr4**	-4.368564028	1.47E-13	1.23E-12	0.048409566
**Oit3**	-3.802136735	8.18E-20	1.92E-18	0.071687394	**Pcdhac2**	-4.385791022	1.27E-06	4.51E-06	0.047834953
**Cbx2**	-3.804100693	2.46E-09	1.20E-08	0.071589872	**Tspyl4**	-4.404543979	7.99E-13	6.03E-12	0.047217191
**Havcr2**	-3.809004329	3.57E-19	7.36E-18	0.071346955	**Syt3**	-4.407541519	0.0016484	0.004169586	0.047119188
**Cgnl1**	-3.812720575	1.22E-19	2.75E-18	0.071163409	**Cngb1**	-4.4108467	0.0008018	0.002101134	0.047011362
**Tnnt1**	-3.81387538	4.81E-05	0.00014431	0.071106469	**Galnt9**	-4.428737485	2.58E-13	2.09E-12	0.046431977
**Zfp286**	-3.83786427	7.81E-05	0.00022945	0.069933898	**Rab7b**	-4.430746686	1.69E-26	1.82E-24	0.046367357
**Neil2**	-3.838094529	5.81E-09	2.70E-08	0.069922737	**Kcnk13**	-4.433766286	4.74E-23	2.11E-21	0.046270411
**Zfp395**	-3.842408044	4.27E-16	5.15E-15	0.069713987	**Palm3**	-4.444368002	9.37E-06	3.05E-05	0.045931637
**Cebpa**	-3.843851028	3.00E-28	5.82E-26	0.069644294	**Rab36**	-4.454268483	2.41E-05	7.49E-05	0.04561751
**Arhgap18**	-3.84472718	8.28E-19	1.59E-17	0.069602012	**Thap8**	-4.457970145	0.0015320	0.0038894	0.045500615
**Dock3**	-3.845089677	8.34E-09	3.82E-08	0.069584526	**Tgfbi**	-4.46040961	2.95E-26	2.98E-24	0.045423743
**Gask1b**	-3.862366417	2.19E-21	6.99E-20	0.068756198	**Dhcr7**	-4.471309373	1.48E-23	7.52E-22	0.045081853
**Eya1**	-3.862700316	7.08E-13	5.38E-12	0.068740287	**Rnase2b**	-4.495751353	0.0019343	0.004855447	0.044324515
**Abcd2**	-3.884117543	3.70E-18	6.35E-17	0.067727355	**Lgals4**	-4.509255777	8.00E-06	2.62E-05	0.043911549
**Abcc3**	-3.894381291	4.42E-16	5.32E-15	0.067247233	**Slc43a2**	-4.518269703	6.49E-28	1.11E-25	0.043638046
**Kcnj2**	-3.904293261	3.62E-19	7.45E-18	0.066786797	**D930048N14Rik**	-4.518381691	3.31E-14	3.02E-13	0.043634658
**Eepd1**	-3.906171046	2.23E-21	7.10E-20	0.066699925	**Lefty1**	-4.523782822	2.73E-06	9.39E-06	0.043471605
**Gpr27**	-3.910470756	6.14E-05	0.00018211	0.066501433	**Zkscan4**	-4.530841445	2.23E-06	7.74E-06	0.043259433
**Slc7a4**	-3.912237875	8.07E-05	0.00023678	0.066420267	**Gpr137c**	-4.538077609	3.03E-05	9.30E-05	0.043042998
**Slc27a6**	-3.917061241	1.16E-08	5.20E-08	0.066198336	**Cbx8**	-4.540239721	2.17E-12	1.54E-11	0.04297854
**D3Ertd751e**	-3.936233216	1.93E-11	1.22E-10	0.065324445	**Kif19a**	-4.554858495	3.65E-09	1.74E-08	0.042545239
**Cntrob**	-3.93817125	2.05E-09	1.01E-08	0.065236751	**Lurap1l**	-4.561914626	0.0017559	0.004428764	0.042337661
**Svip**	-3.950541736	5.96E-14	5.22E-13	0.064679766	**Tchh**	-4.563913896	1.62E-11	1.03E-10	0.04227903
**Pdxk**	-3.950623571	3.00E-26	3.01E-24	0.064676097	**Mapk12**	-4.576736978	0.0013176	0.003375746	0.041904908
**Ccdc92b**	-3.953754572	0.0003413	0.00093097	0.064535886	**Arrb1**	-4.58128592	2.06E-24	1.30E-22	0.041772986
**Kank3**	-3.967139086	9.61E-10	4.91E-09	0.063939927	**Nlrp1b**	-4.587197857	2.54E-09	1.23E-08	0.041602157
**H2bu2**	-3.973186962	7.30E-05	0.00021497	0.063672448	**Gm5127**	-4.59594144	3.43E-06	1.17E-05	0.041350786
**Bora**	-3.97581203	2.72E-07	1.04E-06	0.063556697	**Shc4**	-4.597771126	0.0003218	0.000880353	0.041298376
**Rarb**	-3.975985005	1.01E-05	3.26E-05	0.063549078	**Zfp385c**	-4.619932655	0.0015112	0.00384097	0.040668831
**Shcbp1l**	-3.979246159	0.0003899	0.00105719	0.06340559	**Dkk2**	-4.633667465	5.98E-06	1.98E-05	0.040283491
**Otulinl**	-3.979755643	1.20E-22	4.92E-21	0.063383202	**Chdh**	-4.647959493	4.58E-18	7.65E-17	0.039886394
**Sgsh**	-3.983873698	7.28E-20	1.73E-18	0.063202538	**Cdc25b**	-4.655937774	2.16E-17	3.27E-16	0.039666426
**Nthl1**	-3.990072689	3.31E-10	1.80E-09	0.062931551	**Klc4**	-4.662398211	2.21E-23	1.06E-21	0.039489196
**Trim7**	-4.004371577	3.41E-07	1.29E-06	0.062310902	**Rasl2-9**	-4.683411095	0.0005578	0.001486239	0.038918203
**Matk**	-4.013270895	2.01E-14	1.90E-13	0.061927718	**Il17re**	-4.689302168	0.0013737	0.003513432	0.038759609
**0610040J01Rik**	-4.016532264	6.06E-11	3.60E-10	0.061787882	**Steap3**	-4.69115114	1.43E-24	9.45E-23	0.038709966
**Espl1**	-4.019982936	1.30E-15	1.47E-14	0.061640273	**Cmbl**	-4.715802596	1.32E-06	4.68E-06	0.038054145
**Tmem191c**	-4.025451814	0.0002304	0.00064259	0.061407053	**Gm3854**	-4.718931856	0.0004940	0.001323279	0.037971693
**Mettl7a1**	-4.027708596	4.33E-21	1.30E-19	0.06131107	**Ccr10**	-4.738997373	0.0005933	0.001574881	0.037447225
**Nav2**	-4.027919257	5.66E-16	6.75E-15	0.061302119	**Tmem86a**	-4.739763205	8.69E-27	1.01E-24	0.037427352
**Neurl1b**	-4.028649389	7.47E-08	3.05E-07	0.061271102	**Ypel1**	-4.786536617	0.0012346	0.003173404	0.036233385
**Smpd5**	-4.044017185	7.90E-06	2.59E-05	0.060621897	**Cracr2b**	-4.813785438	1.21E-12	8.91E-12	0.035555451
**H1f2**	-4.048368448	9.72E-24	5.18E-22	0.060439333	**Troap**	-4.828532739	0.0006121	0.001621227	0.035193853
**Bbs9**	-4.048653291	4.67E-12	3.17E-11	0.060427401	**Syt8**	-4.846336094	0.001895963	0.004764188	0.034762217

Gene	log2 (FC)	P-value	FDR	FC	Gene	log2 (FC)	P-value	FDR	FC
**Gm4131**	-4.862735042	0.0006373	0.00168532	0.034369316	**Thbd**	-5.38761922	1.64E-22	6.45E-21	0.023887187
**1190005I06 Rik**	-4.863360731	1.10E-06	3.92E-06	0.034354414	**Dbp**	-5.412570188	3.18E-12	2.21E-11	0.023477617
**Gzmm**	-4.865402801	6.14E-07	2.25E-06	0.034305821	**Treml1**	-5.419880025	3.30E-05	0.000100743	0.023358962
**Efhc1**	-4.86920501	0.0001245	0.00035809	0.034215527	**Btbd17**	-5.425767559	0.0004697	0.001261378	0.02326383
**Hacl1**	-4.881395009	2.46E-11	1.53E-10	0.033927642	**Pcdhgc4**	-5.455839403	0.0005653	0.001504067	0.022783933
**Cdca7l**	-4.888299214	3.95E-20	9.90E-19	0.033765665	**Deptor**	-5.470314229	8.66E-17	1.17E-15	0.02255648
**Ica1**	-4.88917294	0.0012126	0.00311830	0.033745222	**Plxnb3**	-5.606932055	0.0002956	0.000812533	0.020518484
**Mtss1**	-4.890558297	3.29E-24	1.96E-22	0.033712834	**Chek2**	-5.796086369	6.07E-14	5.31E-13	0.017997167
**Fam189b**	-4.893293627	1.88E-11	1.19E-10	0.033648975	**Reps2**	-5.845069833	9.40E-09	4.27E-08	0.017396371
**Misp3**	-4.906730997	5.63E-07	2.07E-06	0.033337021	**Nxpe5**	-5.845676412	7.37E-12	4.89E-11	0.017389058
**Sit1**	-4.9232189	0.0006102	0.00161702	0.032958196	**Armc2**	-5.861705193	5.16E-08	2.15E-07	0.01719693
**Dgkg**	-4.963820706	9.01E-13	6.74E-12	0.032043583	**Wdr90**	-5.886048429	6.21E-07	2.28E-06	0.016909192
**St6gal1**	-4.967711125	6.06E-26	5.62E-24	0.03195729	**Atg9b**	-6.188051737	2.42E-06	8.37E-06	0.013715474
**Cavin2**	-4.978167574	0.0002703	0.00074756	0.031726505	**Map2k6**	-6.218241451	1.40E-06	4.97E-06	0.013431447
**Slc25a35**	-4.978710064	3.45E-12	2.39E-11	0.031714578	**Dhtkd1**	-6.232315174	3.30E-06	1.13E-05	0.013301058
**Nat8l**	-4.996220372	1.13E-09	5.70E-09	0.031331977	**Arhgap19**	-6.275655985	5.23E-24	3.00E-22	0.012907416
**Slc25a27**	-5.015947846	0.0002504	0.00069509	0.030906458	**Cd7**	-6.328196875	2.40E-06	8.33E-06	0.012445803
**Mroh6**	-5.030495433	0.0003625	0.00098593	0.030596375	**Lpar5**	-6.473274634	3.52E-15	3.72E-14	0.011255121
**Pdk2**	-5.040850124	1.09E-15	1.25E-14	0.030377562	**Gm49391**	-6.528952938	0.0013877	0.003547144	0.010829024
**Skint3**	-5.121698492	0.0001009	0.00029296	0.02872203	**Abcg3**	-6.600806414	2.90E-17	4.29E-16	0.010302895
**Mblac2**	-5.127455233	2.55E-14	2.37E-13	0.028607649	**Pram1**	-6.688359337	0.0004684	0.001257933	0.009696237
**Rtn4rl1**	-5.149151474	0.0003394	0.00092639	0.028180647	**Olfr1506**	-6.85360778	1.45E-05	4.61E-05	0.008646861
**Hey1**	-5.15073473	7.23E-10	3.76E-09	0.028149737	**Osbp2**	-6.857314828	8.92E-08	3.61E-07	0.008624671
**Adam23**	-5.187717957	0.0003996	0.00108187	0.027437295	**Echdc3**	-7.21041514	1.38E-08	6.13E-08	0.006752254
**Cd300lb**	-5.272839773	1.56E-28	3.34E-26	0.025865273	**Bmx**	-7.534139353	2.71E-09	1.32E-08	0.005395082
**Rell2**	-5.274671998	0.0006187	0.00163792	0.025832445	**Gm20388**	-7.759007676	0.0029237	0.007164768	0.004616427
**Lpin1**	-5.349088754	2.08E-20	5.49E-19	0.024533744	**Dppa3**	-7.859371146	3.61E-09	1.72E-08	0.004306193
**Tns1**	-5.385004577	1.06E-23	5.63E-22	0.023930518	**Engase**	-9.586671441	9.52E-14	8.12E-13	0.001300542

FC: Fold change; FDR: False discovery rate
